# Fungus above us: exploring the atmospheric mycobiome by aircraft

**DOI:** 10.7717/peerj.21746

**Published:** 2026-09-30

**Authors:** Kimberly L. Métris, Jérémy Métris

**Affiliations:** 1 Department of Genetics and Biochemistry, Clemson University, Clemson, SC, United States of America; 2 School of Health Research, Clemson University, Clemson, SC, United States of America; 3 Airborne Science LLC, Clemson, SC, United States of America

**Keywords:** Aerobiology, Air mycobiota, Airborne DNA, Atmospheric microbiome, Environmental DNA, Fungal aerobiome

## Abstract

Fungi play a key role in ecosystems, influencing the global bioaerosol budget and pollution dynamics even when metabolically inactive. Despite this, diversity and transport of fungi in the atmosphere are not well explored. This study shows that the atmosphere contains diverse fungi with varied ecological classifications and recruitment patterns reflecting underlying terrestrial habitats. Abundant genera include *Neoascochyta*, *Cladosporium*, *Alternaria*, *Coniothyrium*, *Penicillium*, *Hymenochaetopsis*, *Lachnum*, and *Fonsecazyma*. The atmospheric mycobiome is dominated by decomposers and pathogens; over 40% of the sampled airborne fungi are putative plant or animal pathogens. Using aircraft surveys from 2022 to 2023 in distinct seasons alongside comprehensive environmental datasets, airborne fungal diversity was found to decline with altitude and increasing vegetation productivity. Patterns of diversity in atmospheric fungal communities are shaped by vegetation productivity and meteorology. Remote sensing and meteorological data provide valuable insight into this diversity and reinforce the role of local or regional phenology as an important determinant of atmospheric biodiversity. Additionally, the proportional composition of functional guilds in the atmosphere shifts with vegetation productivity and surface winds, suggesting that seasonal transport dynamics may contribute to shaping assembly of the atmospheric mycobiome. This work demonstrates how integrated genomic and environmental datasets acquired by aircraft and satellite data could be leveraged in future to clarify aerobiology questions and contribute to a unified view of fungal ecology.

## Introduction

Fungi are among the most important biota on the planet, mediating ecosystem processes and contributing to the global bioaerosol budget and pollution even when metabolically inactive (Janssen et al., 2021; Moelling & Broecker, 2020; Šantl-Temkiv et al., 2022). The number of fungal species has been estimated to be near 3.8 million (Hawksworth & Lücking, 2017) but many are still unknown (Peay, Kennedy & Talbot, 2016). Fungi mediate processes as decomposers, pathogens, and mutualists in terrestrial systems and play important roles in meteorology and atmospheric chemistry, including cloud nucleation and precipitation formation, biogeochemical cycling, and climate alteration (Amato et al., 2005; Behzad, Gojobori & Mineta, 2015; Fröhlich-Nowoisky et al., 2009; Morris et al., 2011). Recent evidence suggests that exposure to a diverse or specific atmospheric mycobiome could be necessary to be healthy (Robinson & Breed, 2023). Many airborne fungi have consequences for plant, animal, and human health, and have been linked to short and long distance spread of disease (Dillon & Dillon, 2021; Smith et al., 2012). Fungi are responsible for numerous plant diseases, including blights, rusts, and rots leading to considerable losses of economically important crops and pose a threat to food security (Rizzo et al., 2021). Airborne fungal infections can also lead to poor health outcomes in immunocompromised people, including allergies, asthma, cardiovascular and respiratory disease, cancer, pathological changes in intestinal microbiota, and reduced life expectancy (Moelling & Broecker, 2020). Despite their ecological and economic importance, the diversity and distribution of fungi comprising the atmospheric mycobiome (aeromycobiome) remain largely unknown.

Molecular ecological data of fungi allow tracking of biodiversity, climate change, and disease, and while such datasets are expanding for ecosystems such as soil, water, humans, and other animals (Fierer et al., 2012; La Cono et al., 2011; The Human Microbiome Project Consortium, 2012), the atmosphere has been understudied in comparison (Behzad, Gojobori & Mineta, 2015). However, recent global scale ground-level air sampling provides strong evidence that the composition of airborne fungal communities is closely linked to host phenology, with significant ecological impacts driven by dispersal strategy (Abrego et al., 2024). Lack of understanding surrounding fungal deoxyribonucleic acid (DNA) vertical transport, resuspension, and dispersal dynamics is due largely to challenges associated with sampling. While many airborne DNA studies focus on ground-level or stationary sampling within habitats with restricted airflow, *i.e.,* not in open spaces (Abrego et al., 2024; Dietzel et al., 2019; Ovaskainen et al., 2024; Prospero et al., 2005; Straumfors et al., 2021; Wagner et al., 2022), such methods can limit substrate differentiation from the surface, resulting in incongruent or overly similar taxonomic profiles (Drautz-Moses et al., 2022). In contrast, tropospheric aircraft surveys, while inherently resource-intensive and more limited in number than ground-based surveys, can provide high vertical resolution. Atmospheric aircraft surveys from 2014, for instance, provided evidence of long-distance transport of fungi including potential human pathogens (Rodó et al., 2024). Recently, there has been growing interest in studying bioaerosols in extreme environments, particularly in the upper atmosphere where biodiversity is present but biomass is often low (Mainelis, 2020). Advances in high-integrity active air filtration for nucleic acids, benchmarked against existing methods (Métris & Métris, 2023) now offer a breakthrough opportunity to uncover terrestrial-atmospheric patterns through bioaerosol sampling (Johnson & Barnes, 2024). Aircraft present an effective platform for aerial survey studies, allowing precise control of altitude and flight paths over large areas to monitor bioaerosols efficiently in a scalable manner (Meier & Lindbergh, 1935; Aguilera et al., 2021; Métris & Métris, 2023).

The Southeast United States (SEUS) contains a mixture of natural and agricultural land with areas of strong biogenic emissions and frequent convective weather (Ziemba et al., 2016). The SEUS is a substantial source of bioaerosols emitted from different land types and convection permitting their vertical transport (Ziemba et al., 2016). Recent models of global drivers of the fungal spore bioaerosol budget point to high fungal spore and mass concentration emissions with a hotspot or peak in the SEUS (Janssen et al., 2021). It remains unclear whether fungal communities are more similar within particular air masses than among them (Womack, Bohannan & Green, 2010). It is believed that well mixed profiles of fungal spores characterize the atmospheric boundary layer, but airborne collection and sequencing confirming or refuting this assumption are lacking particularly above aquatic and terrestrial sources, including terrain such as ground, tree canopies, and manmade structures. A need exists to connect microbial diversity at defined altitudes above the ground with atmospheric conditions and biological traits from sites that can be affected by local emissions from different land types (Amato et al., 2023). Temporal variation (both daily and seasonal) and spatial variation (due to local sources and air masses) of fungal aerobiota can provide insight into transport mechanisms, as biological traits of airborne microorganisms may influence large-scale dispersal (Xue et al., 2022), and examining vertical patterns at stratified heights can provide insight into atmospheric microbial movement (Větrovský et al., 2019). Moreover, it is possible to identify an alternative hypothesis, the possibility of sampling local microorganisms, given the highly specific biological and ecological associations of certain fungi (Smith et al., 2012).

The aim of this study was to investigate the biodiversity and spatiotemporal variation (daily and seasonal as well as horizonal and vertical) distribution of atmospheric fungi due to local sources and air masses to gain a more complete view of airborne fungal diversity toward building solutions for monitoring. Fungal DNA isolated from air filtered during dedicated research flights hundreds to thousands of meters above the ground in the SEUS was used to address the following research questions: What are the community profiles of fungi in the atmosphere at specific altitudes, and does composition reflect terrestrial habitats in the area (RQ1)? Are fungal communities more similar within particular flights or air masses than among them (RQ2)? Are there ecological differences in atmospheric fungal diversity and distribution (RQ3)? In this study, targeted sequencing of the fungi nuclear ribosomal internal transcribed spacer (ITS) region, an effective marker for most but not all fungal species (Abrego et al., 2024; Schoch et al., 2012) is integrated with environmental (meteorological data and air mass transport models), remote sensing (*i.e.,* satellite data of normalized difference vegetation index of plant health, NDVI), and trait information (FUNGuild and FungalTraits) in a combined analysis of biodiversity and ecological patterns to characterize the atmospheric mycobiome and to better understand biogeography patterns in airborne fungal composition and identify possible ecological drivers. Air samples were expected to contain fungi commonly found in the area overflown, with greater fungal diversity closer to productive vegetation (their presumed growth substrate) (Lymperopoulou, Adams & Lindow, 2016; Sindt, Besancenot & Thibaudon, 2016). Clear differentiation was expected between seasons due to short-term seasonal variation, with less pronounced differences vertically between air masses because bioaerosols should circulate, homogenize, and assort stochastically in the atmosphere.

## Materials and Methods

### Sampling, sequencing, and bioinformatics

For complete information on study design, sample collection, DNA extraction and sequencing, bioinformatics workflows, and technical data validation, see elsewhere (Métris & Métris, 2023). The steps are outlined here (Fig. 1).

**Figure 1:**
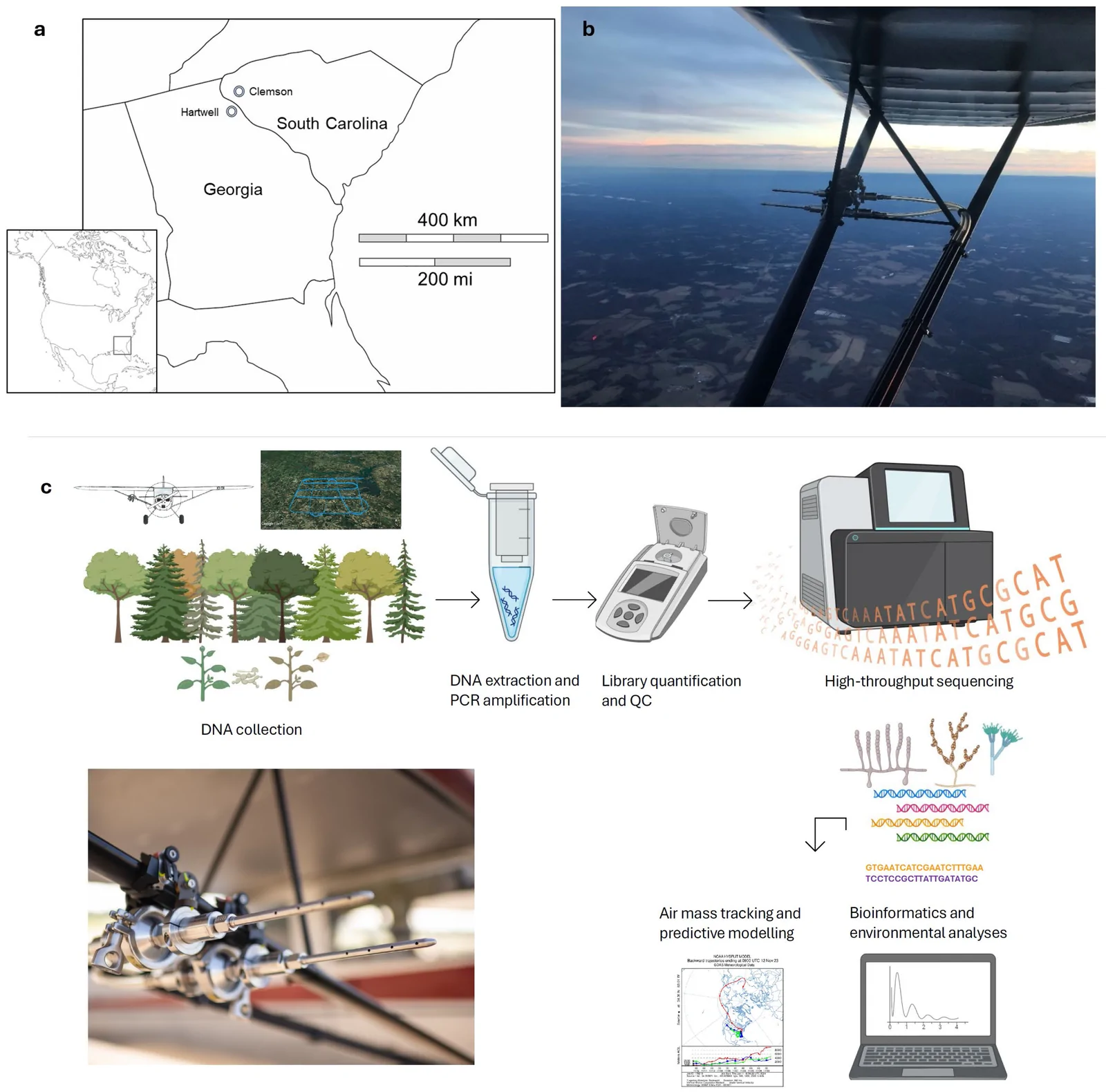
Geographic location, aircraft instrumentation, and experimental design. (A) Map of study location in the southeast US with state and nearest city names was adapted from database record no. 1683 at http://www.d-maps.com. (B) High-integrity capture system featuring duplicate sampling probes mounted on aircraft wing strut during the research flights reported in this study. Image of atmospheric DNA survey flight with aerosol haze in background (B) taken by author, K. Métris during sampling FLT10 conducted 12-November-2023. (C) Experimental design of methods collecting fungi from the atmosphere for this study, where airborne DNA was filtered on aircraft surveys over different land areas and seasons, and amplicon sequence data were combined with statistical analyses (PCA) of environmental data in addition to backward (possible source) and forward (predictive) air mass transport models. Inset photo was taken by Clemson University and laboratory graphics were created in BioRender (Created in BioRender. Metris, K. (2024) BioRender.com/b70d892).

Air sampling flights were conducted in the vicinity of Hartwell, Georgia (GA) and nearby Clemson, South Carolina (SC) in the temperate SEUS (Fig. 1). Sentinel-2 satellite ArcGIS data were obtained from https://livingatlas.arcgis.com/. Samples were collected at vertical heights above the ground of 300 m, 1200 m, and 2500 m, representing altitudes above and within the atmospheric boundary layer, the part of the troposphere that is in direct contact with the surface (Fig. 2). The weekly mean boundary layer height over the SE climate region in the continental US has been found to remain below 2,500 m (mean 1,684 m) (Ayazpour et al., 2023). This study focused on the fate and transport of fungi specifically in the atmosphere and cannot directly address fungal diversity on the ground. The methods used in this study cannot assess viability and as such we neglect the fraction of the total atmospheric mycobiome that is transcriptionally (metabolically) active because nonviable bioaerosols can contribute to the global bioaerosol budget, influence atmospheric and surface conditions, and impact health.

**Figure 2:**
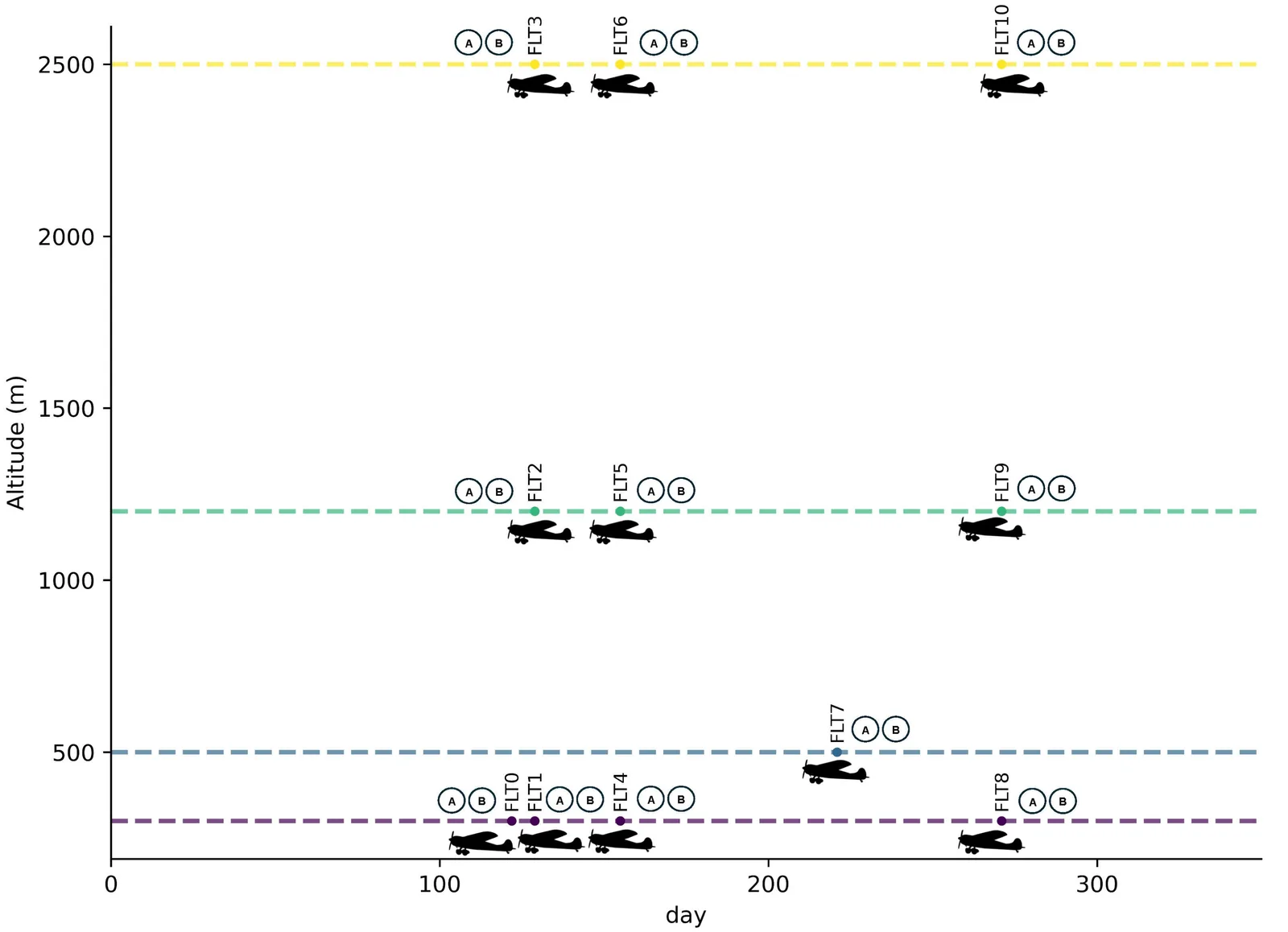
Sampling framework across seasons and altitudes in this study. Dashed lines represent altitude above ground level (300 m, 500 m, 1,200 m, 2,500 m). Dots on the altitude lines indicate the Julian day of the year of that flight. Duplicate (A and B) filter samples were collected for every flight to ensure comprehensive and robust sampling and increase yield of DNA captured. All flights occurred in the southeastern United States at one site in GA, except FLT7 in nearby SC. We report time as Julian days because it provides a continuous, unambiguous scale for seasonal analyses and avoids inconsistencies introduced by varying month lengths and calendar formats to allow for comparison across ecological and phenological studies. Created in JupyterLab with Python.

Flight times totaled 13 h 6 min of sampling (averaging 71.4 min per flight). All flights were conducted during the day to avoid diurnal effects. Data on flights, flows, and times were recorded (Tables S1.1 and S1.2). A grid flight pattern (Métris & Métris, 2023) was used with the exception of the flight over SC added to capture increased human activity in the same region within a 10 NM box centered on the university stadium at 500 m remaining outside the restricted area (Fig. S1). Flight data were recorded with ForeFlight^^®^^, and representative tracks are provided (Fig. S1). A total of 7 flights (FLT0-FLT6) were conducted in spring following the equinox and prior to the summer solstice. The remaining flights in autumn (FLT7-10) were conducted on the equinox and prior to the winter solstice (Fig. 2).

Instrumentation sterilization, air sampling, and DNA extraction followed previous work (Métris & Métris, 2023). In brief, a high integrity sampler (Fig. 1) was fitted with a sterile cellulose nitrate filter with a diameter of 47 mm and pore size of 0.22 µm (0.45 µm for FLT7) (MDI Membrane Technologies, Inc., Harrisburg, PA, USA) housed in the isolation chamber. The sampling instrumentation filters air with a vacuum pump to capture intracellular or extracellular DNA. Each sterilized sampling probe was used for one flight and altitude collection. Sampling commenced upon reaching the target altitude and the first Global Positioning System (GPS) coordinate of the transect grid during straight-and-level flight by turning on the vacuum pump and closing the release valve (thus opening the check valve) and terminated prior to vacating that altitude by reversing this procedure (Métris & Métris, 2023). To maximize the amount of air sampled, duplicate filters were collected (1,350–4,410 L of air per sample). DNA was extracted from the 22 filters using the E.Z.N.A.^^®^^ Soil DNA Kit (Omega Bio-Tek, Norcross, GA, USA) following the manufacturer’s protocol and modifications detailed elsewhere (Métris & Métris, 2023). All samples were processed with the soil kit with the exception of FLT7, where the E.Z.N.A. pathogen kit was substituted due to availability. DNA extractions of biological replicates from each flight (duplicates) were combined to increase DNA yield.

One sample (FLT7) was collected using a different pore size filter and different DNA extraction protocol than all other samples. Analyses were conducted both with and without FLT7 because the sample provided valuable information but differed in filter pore size, DNA extraction kit, and site, limiting attribution of observed variation in taxon richness or functional guild composition to a single methodological or biological factor. Air volume and duration of sampling were recorded for all samples, and our filters were enclosed in an isolation chamber to guard against loss, reducing a major source of methodological variation because differences in richness can otherwise arise from unequal sampling effort or DNA capture opportunity.

### Library preparation and DNA sequencing

A subset of the airborne DNA samples was originally collected for bacterial, plant, and vertebrate detection in a previous study demonstrating technical operability of the sampling probe (Métris & Métris, 2023) but did not include fungal-specific DNA markers. In this study, amplicon libraries were constructed using universal primers for fungi appended with 5′ Illumina^^®^^ adapters: (F) 5′ -ACACTCTTTCCCTACACGACGCTCTTCCGATCT-3′ and (R) 5′ -GACTGGAGTTCAGACGTGTGCTCTTCCGATCT-3′: Fungi: ITS86 (F): 5′ - GTGAATCATCGAATCTTTGAA-3′, ITS4 (R): 5′ -TCCTCCGCTTATTGATATGC-3′ for the fungal-specific ITS2 gene region (Sánchez-Parra et al., 2021). Purified fungal ITS2 amplicon replicates representing each flight and altitude were pooled by flight for high-throughput amplicon sequencing targeting ∼50,000 reads per sample on the Illumina^^®^^ MiSeq platform (2 × 250 bp paired end reads) in two sets (2022 and 2023) at Azenta Life Sciences (South Plainfield, NJ).

Negative control processing details are outlined elsewhere (Métris & Métris, 2023). Briefly, sets of negative controls were incorporated for QA/QC of storage, cleaning and sterilization, field campaigns, molecular lab use, and bioinformatic processing. Negative controls from equipment, flights, and lab (extraction blanks and no template polymerase chain reaction (PCR)) were pooled undiluted in equal volume (Verkuil et al., 2022) and included in both MiSeq runs.

### Sequence read assembly, filtering, and processing

Raw sequencing data were deposited in the NCBI Sequence Read Archive (SRA) database under BioProject PRJNA1169604. FASTQ files for flights and pooled negative controls were processed using LotuS version 2.21 (Hildebrand et al., 2014; Özkurt et al., 2022) on the EU Galaxy server with default parameters for Illumina MiSeq ITS2 amplicon data unless noted. LotuS2 employs stringent probabilistic and Poisson binomial quality filtering (Puente-Sánchez, Aguirre & Parro, 2016) and seed extension to maximize reads in taxon abundance estimates and Operational Taxonomic Unit (OTU) sequence lengths for taxonomic classification (Özkurt et al., 2022). Reads were dereplicated using 8:1, 4:2, and 3:3 filtering thresholds. The LotuS2 dereplication filters (meeting any of the following criteria: appearing at least eight times in total across all samples and in at least one sample (8:1), *or* appearing at least four times in total and in at least two different samples (4:2), *or* appearing at least three times in total and in at least 3 different samples (3:3)) implemented here are default parameters for the program optimized for the chosen clustering method to exclude low abundance artefacts and sequencing/PCR errors and retain biologically real sequences across multiple samples. OTUs were clustered with VSEARCH version 2.21.2 (Rognes et al., 2016) and chimeras were detected with UCHIME2 version 11 (Edgar, 2016) against the UNITE ITS chimera database (Nilsson et al., 2015). Reads were screened against the UNITE ITS taxonomic reference database (Kõljalg et al., 2013) to identify fungal ITS2 matches. Because ASVs are unsuitable proxies for species in ITS-based fungal analyses (Kauserud, 2023), we used OTU clustering for this biodiversity survey, as it preserves broad-scale ecological patterns when processing metabarcoding data (Abrego et al., 2024; Rzehak et al., 2024).

Filtered taxonomic tables were generated following removal of the number of reads in the pooled extraction negative and no-template PCR controls from the OTUs. Relative to the study samples, these controls contained few fungal reads typically of the more common OTUs (Tables S1.3 and S1.4). Thus, any OTU represented exclusively in a negative control (not appearing in any samples) was excluded from analysis. Fungal OTUs were quantified based on read count, with those exceeding 1,000 reads considered dominant and those below classified as minor (rare) components, following Rosa et al. (2020). Read counts can be influenced by DNA extraction, PCR efficiency, and primer bias (Medinger et al., 2010), potentially leading to misinterpretation of abundance (Weber & Pawlowski, 2013). However, Giner et al. (2016) and Hering et al. (2018) found that these biases do not distort the proportionality between reads and cell abundance, supporting the use of read counts as a proxy for mean relative abundance. Therefore, this threshold was used to distinguish dominant from minor OTUs in air samples. OTU relative abundances (or OTU presence/absence) were used for comparisons between dominant and minor groups.

### Airborne fungal diversity analyses

Dereplicated, chimera-filtered OTU tables were imported into Microbiome Explorer Namco version 1.1 (Dietrich et al., 2022) with default normalization settings including total sum scaling to 10,000 reads and filtering abundance of OTUs at greater than 0.25% abundance prior to analysis. Area proportional Venn diagrams were constructed to compare relationships between shared and unique OTUs with BioInfoRX (https://demo.biolims.org/apps/venn.php).

In an exploratory attempt to parse airborne fungal OTUs by ecological guild (trophic mode), Python-based FUNGuild (Nguyen et al., 2016) was run in Anaconda version 2.5.0 (https://www.anaconda.com/products/individual). Functional annotations of ecological roles and habitat preferences were cross-checked using FungalTraits version 0.0.3 (Põlme et al., 2020).

### Atmospheric transport and environmental analyses

Atmospheric circulation can greatly influence aerobiome diversity (Cáliz et al., 2018; Elmassry et al., 2021). Thus, we asked whether airborne fungi assemblages were more similar between or within specific air masses and whether there is a link between taxa occurrence and the origin of the air mass moving through the area and altitude where each flight was conducted. Hybrid Single-Particle Lagrangian Integrated Trajectory (HYSPLIT) modelling (https://www.arl.noaa.gov/) was used to create kinematic backward trajectories of air parcels moving through the air over both 3 and 10 day periods from the coordinates of the collection site to infer air mass transport history and possible origins, and forward trajectories to examine long range transport potential. To assess transport of local air masses, a standard trajectory analysis with increased simulations was used to derive more quantitative inferences, but aerosol processes were not introduced because deposition models primarily account for inorganics (*e.g.*, dust, pollutants) rather than biological particles and the objective was air parcel general movement. HYSPLIT trajectories were modelled over 10 days since transcontinental sources have been shown to take 9 days to travel to/from North America (Smith et al., 2012).

Meteorological conditions, including wind speed, gust, and direction, daily mean surface air temperature and temperature at altitude, relative humidity, dewpoint, cloud cover, sea level pressure, density altitude (pressure altitude corrected for non-standard temperature or air density above mean sea level), solar radiation, solar energy, and UV index were recorded for each flight (Table S1.1). Data for each flight and the 12 h prior to sampling were retrieved using the weather query builder at https://www.visualcrossing.com/weather/weather-data-services. The two readings closest to sampling were averaged if the flight fell outside the hour interval.

To investigate potential seasonal patterns in fungal composition relative to plant productivity (vegetation greenness) where flights were conducted, Moderate Resolution Imaging Spectroradiometer data of the Normalized Difference Vegetation Index (NDVI) were extracted at 1 km resolution from the Terra satellite as a proxy for dry matter productivity or greenness. Higher NDVI values generally indicate greener, more lush croplands, forests, or other vegetation, and lower values correspond to low photosynthetic activity (*e.g.,* unhealthy vegetation or exposed surfaces). These data are archived by the Application for Extracting and Exploring Analysis Ready Samples at https://appeears.earthdatacloud.nasa.gov/.

### Principal components analysis of environmental data and PC regression

Principal components analysis (PCA) was used to reduce dimensionality of the variables in the dataset and generate orthogonal axes of composite environmental gradients represented by the principal components (PCs). Qualitative descriptors were first transformed using the PRINQUAL procedure with optimal scaling in SAS (SAS Institute Inc., Cary, NC, USA) before inclusion in the PCA. PCA with maximum total variance was performed on the combined quantitative and transformed qualitative descriptor correlation matrices for all flights using R and visualized in JMP Pro version 18 (SAS Institute Inc.).

Major environmental gradients represented by scores from the first three PCs (PC1, PC2, and PC3) were used as predictors in a multiple regression model in R across all flights with fungal OTU richness as the response variable: OTU Richness ∼ PC1 + PC2 + PC3. Because many variables in the environmental dataset are correlated and may have combined effects (*e.g.*, rainfall can both trigger release of bioaerosols from vegetation and remove bioaerosols through deposition), this approach minimizes predictor multicollinearity while preserving major gradients of environmental variation.

### Selection of candidate focal environmental predictors

Ranked loadings of the variables contributing to the first 3 PCs were used to select focal candidate environmental predictors (explanatory variables) contributing to each gradient with low multicollinearity for the remaining ordination and regression analyses. When the highest-loading variable was less practical to measure (density altitude) or less informative for future studies, the variable with the next highest loading was selected (Fig. S3A). Selected focal predictors were PC1-Temperature, PC2-Windspeed, and PC3-Altitude. Additionally, NDVI, the highest-loading variable associated with seasonality within the significant environmental gradient (PC2), was selected as a temporal covariate. The four candidate predictors had variance inflation factor values well below accepted thresholds for problematic multicollinearity, VIFs < 5.

### Ordination analyses of airborne fungi community composition

Community analyses of beta diversity were used to explore compositional variation in airborne fungal communities. Bray–Curtis dissimilarity matrices were calculated from OTU abundance tables using the *vegan* package in R. Nonmetric Multidimensional Scaling (NMDS) ordination was performed on dissimilarity matrices in two dimensions with up to 100 random starts. NMDS stress values were used to evaluate ordination fit. Environmental vector fitting was conducted *post-hoc* using the *envfit* function with 999 permutations to project environmental gradients onto NMDS ordination space using the selected focal predictors to assess relationships between fungal community composition and environmental gradients. Mantel tests were used to evaluate correlations between Bray–Curtis dissimilarities in OTU relative abundance and Euclidean distance matrices for environmental variables. Mantel correlations were computed with missing values removed and using Spearman rank correlations with 999 permutations. Analyses were conducted including and excluding FLT7.

### Multiple regression of airborne fungi taxon and guild richness

To evaluate relationships between atmospheric fungal diversity and environmental predictors, we fitted a series of multiple linear regression models using *lm* in R separately for two response variables: log(taxa richness + 1) and log(trophic guild richness + 1), where guild richness was defined as the number of taxa assigned to each guild. Analyses were performed with and without FLT7: For each dataset, three models were evaluated:

1. *Null model*: log(taxa or guild richness+ 1) ∼1
2. *Primary model*: log(taxa or guild richness + 1) ∼Altitude + Temperature + Windspeed This model assumes meteorology dependence where the response variable varies systematically with altitude, temperature, and wind.
3. *Seasonal model*: log(taxa or guild richness + 1) ∼Altitude + Temperature + Windspeed + NDVI.

This model assumes that the response variable additionally shows variation that systematically depends on temporal vegetation productivity.

The null model included only an intercept. The primary model incorporated altitude, mean daily temperature, and windspeed as fixed effects to assess whether these environmental predictors improved model fit relative to the null model. The seasonal sensitivity model further included NDVI as an additional predictor to evaluate whether temporal vegetation productivity improved explanatory power beyond the primary model. Model selection among these variants was performed using Akaike’s Information Criterion (AIC) and regression parameter estimates. All models were compared using analysis of variance (ANOVA). Variance inflation factors (VIFs) were calculated using the *car* package in R. To test for temporal autocorrelation, residuals from each fitted linear model were ordered by day of year and evaluated using Ljung–Box tests (lag = 1) at α = 0.05 (Burns, 2003).

Finally, to evaluate potential non-independence among samples collected on the same date, linear mixed-effects models with the original fixed effect predictor structure and adding sampling date as a random intercept were fitted using the *lme4* and *lmerTest* packages in R (R Core Team, 2024) as a sensitivity analysis:

*Mixed-effects model with date*: log(taxa or guild richness + 1)∼fixed effects + (1 — Date)

## Results

### Sequence data characteristics and taxonomic composition

A total of 431,104 quality filtered fungi ITS2 reads were obtained from 24,413 L of air across all flights after dereplication and excluding reads from negative controls and nonfungal eukaryotes (Tables S2.1–S2.3). From these reads, 166 fungal OTUs were recovered (Tables S2.2–S2.3). The number of reads per flight ranged from 5,168 to 95,573 (mean = 39,191) and per OTU ranged from 3 to 137,330 (mean = 2,597) (Fig. S2 and Tables S2.1–S2.3). A total of 3,751 fungal reads could not be assigned to a phylum. On days when multiple heights were sampled, more fungi reads were recovered at higher altitude (Table S2.1). The number of sequences recovered varied by flight and some accumulation curves did not reach saturation. As such, increased sampling depth may detect additional OTUs and FLT1/FLT2 in particular could potentially artificially deflate diversity metrics (Fig. S2). As the sequencing depth (number of sequences obtained for each sample) of dereplicated samples ranged from 5,168 to 95,573 as noted above), default normalization (total sum scaling to 10,000), relative abundance and ordination methods (*e.g.,* NMDS/Bray–Curtis), plus threshold limits (total read counts as a proxy for mean relative abundance) and log-transformation to control for variation in sequencing depth were chosen to retain the majority of high depth data and rare OTUs as well as quality-controlled, filtered, normalized data from the two flights with lower sequencing depths.

### Fungal diversity and distribution among air masses

Air samples contained a broad range of molds, yeasts, cup, and sac fungi. Ascomycota (AMC) was the most common phylum, followed by Basidiomycota (BMC). Mortierellomycota, Mucoromycota, and one unidentified phylum were each detected in a single OTU. Similar OTU proportions were represented between AMC (82 OTUs, 49.4% of total OTUs) and BMC (81 OTUs, 48.8% of total OTUs) (Table S2.3). Higher proportions of reads were recovered from AMC compared to BMC (Tables S2.1–S2.3). Sequencing of ITS2 revealed 92 genera and 83 unique species (AMC = 38 species; BMC = 45 species). Samples were enriched with *Neoascochyta desmazieri, Cladosporium cladosporioides, Coniothyrium* spp., *Alternaria alternata*, and *Penicillium coffeae* (Fig. 3). Enriched taxa were frequently related at the taxonomic rank of order, *e.g., N. desmazieri*, *Curvularia americana*, *C. sorghina*, and *A. alternata*, or *C. cladosporioides, Ramularia eucalypti*, and *R. vizellae* (Table S1.5).

**Figure 3:**
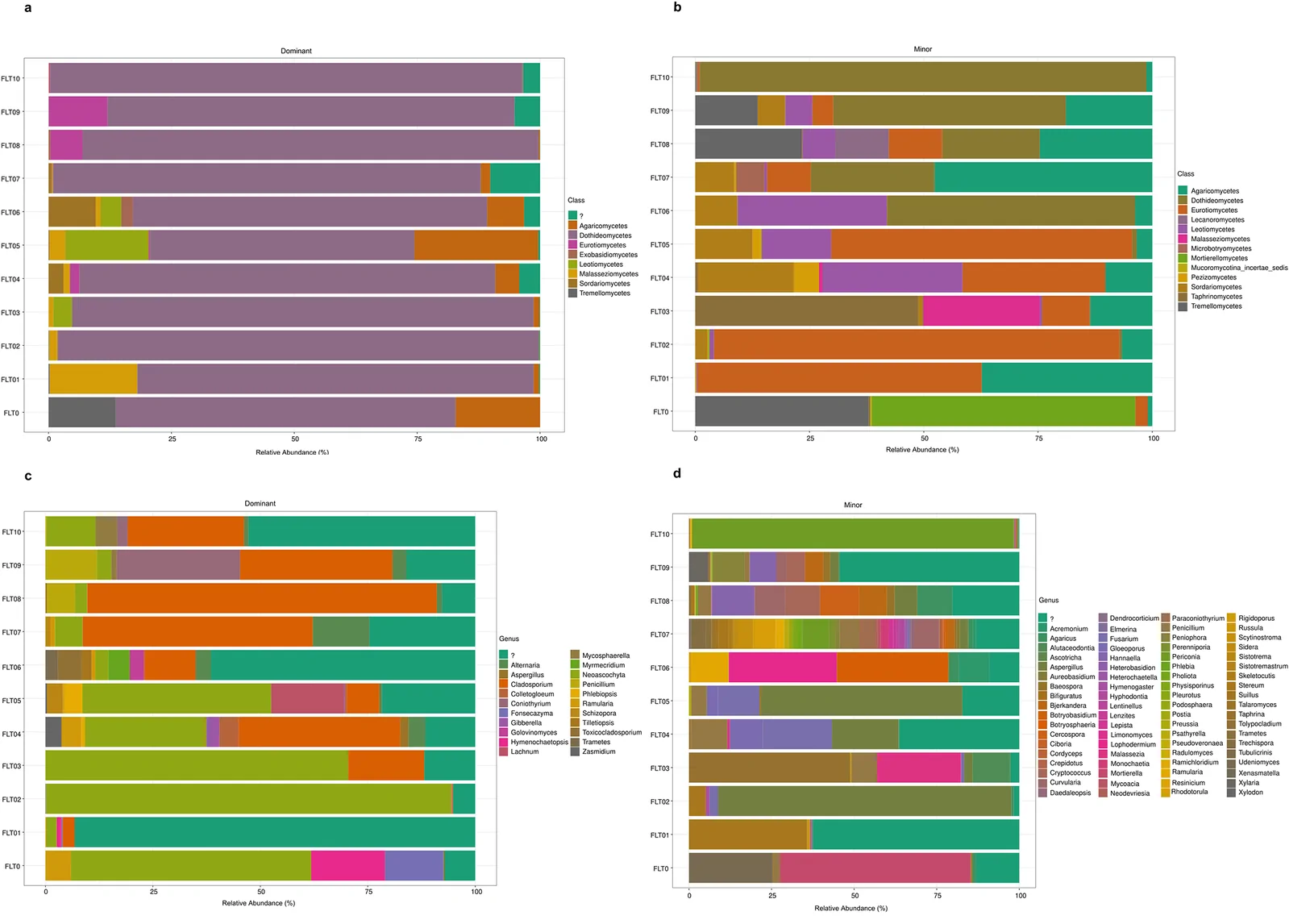
Taxonomic composition (% relative abundance) of airborne fungal DNA across 11 aircraft survey flights. Taxonomy shown at the class level for dominant (A) and minor OTUs (B) and at the genus level for dominant (C) and minor OTUs (D).

A total of 38 OTUs were dominant taxa in the air samples. Additionally, 128 minor OTUs were detected, likely representing the rare or transient portion of the air mycobiome. The number of dominant OTUs ranged from 15 to 27 (mean = 20.27), the most collected on FLT6 at 2,500 m (Figs. 4A, 4B). The number of minor OTUs ranged from 8 to 92 (mean = 25.09) (Fig. 4B). Increased levels of heterogeneity were noted when more minor taxa were present compared to dominant taxa (Fig. 4C and Fig. S5). Among fungi OTUs above 5% prevalence (152 OTUs), only 33 OTUs were detected in spring alone (Fig. 4D). Half (76 OTUs) were detected at both sampling sites (Fig. 4E). Many taxa were detected at all heights, although the total number of OTUs decreased with altitude (Fig. 4F). Fungal diversity decreased with altitude, and more unique OTUs were often detected closer to the ground (Figs. 4F, 4G). Atmospheric fungal OTU and species richness also generally decreased with altitude even on flights at the same place and day, but this effect was not statistically significant (Figs. 4G, 4H and Fig. S4). In some cases, fungal diversity peaked in air masses with modelled trajectories that sank from higher altitudes or contacted the ground where it was possible to contact biota at the surface (Fig. 5). For example, FLT8 captured the highest number of dominant and minor OTUs that day (Fig. 4), including many unique taxa—Lecanoromycetes (observed only then) and *Cercospora canescens*, *Curvularia americana*, and *Penicillium glabrum* (not observed on other flights that day). Backward trajectory analyses inferred that this air mass was close to Earth’s surface days prior to sampling (Fig. 5).

**Figure 4:**
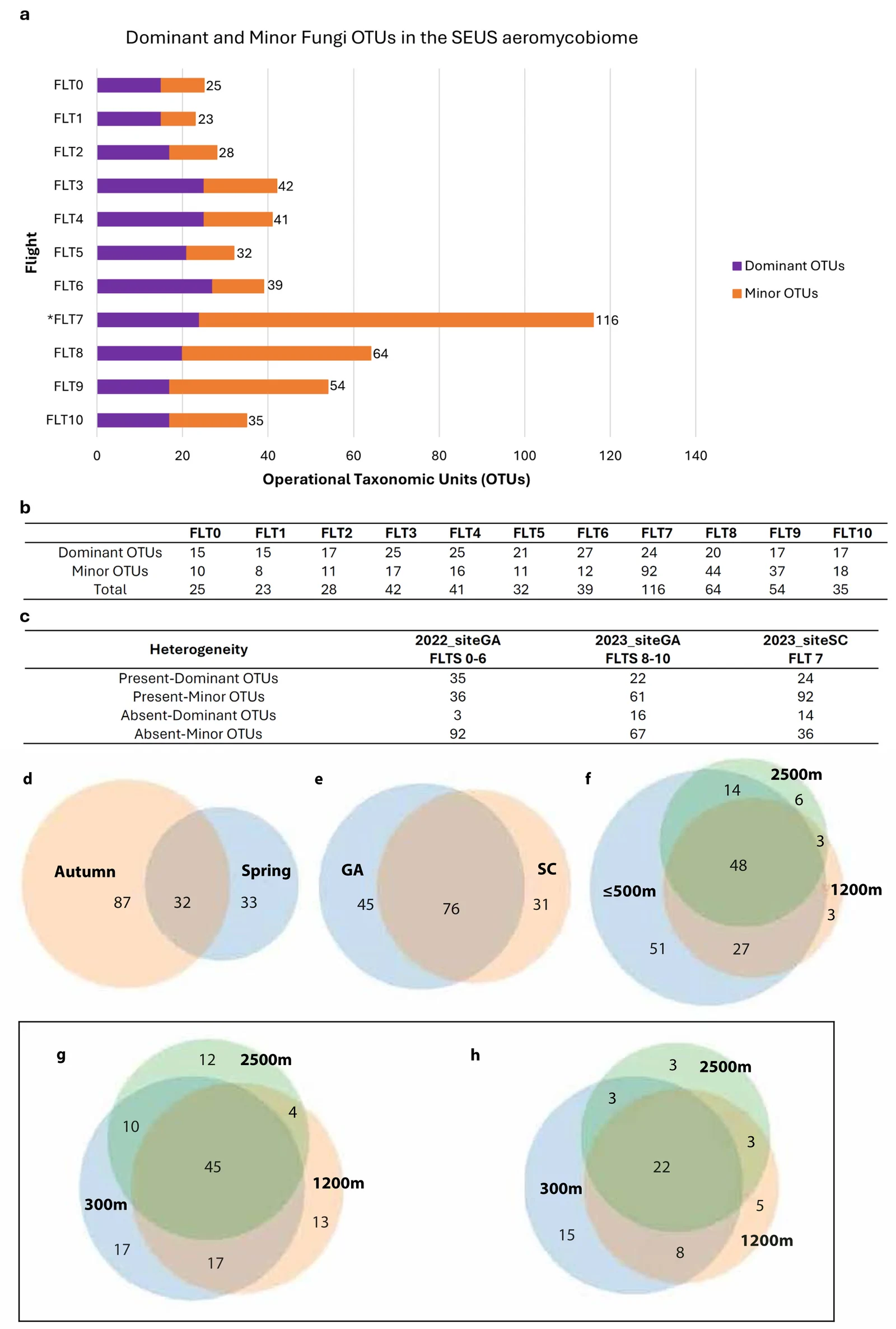
Compositional heterogeneity of atmospheric fungal OTUs. (A) Number of operational taxonomic units (OTUs) recovered in this study. Dominant OTU richness is shown in purple, and minor OTU richness is shown in orange. *FLT7 corresponds to Clemson (SC) sampling; all others are Georgia (GA). Altitudes above ground level: FLT0 (300 m), FLT1 (300 m), FLT2 (1,200 m), FLT3 (2,500 m), FLT4 (300 m), FLT5 (1,200 m), FLT6 (2,500 m), FLT7 (500 m), FLT8 (300 m), FLT9 (1,200 m), and FLT10 (2,500 m). (B) Counts of OTUs recovered on each flight. Heterogeneity of dominant and minor OTUs present or absent at each year_site (C). (D–H) Area proportional Venn diagrams of number of shared and unique OTUs above 5% prevalence (152 OTUs). Groups compared include autumn 2023 and spring 2022 seasons (D), GA and SC sites (E), and altitudes (F) ≤ 500 m, 1,200 m, and 2,500 m above ground level. Inset: Number of OTUs (G) and species (H) detected on the same day at the same site at three altitudes (300 m, 1,200 m, and 2,500 m agl).

Probable transport histories of high diversity samples (more OTUs) typically originated from descending air masses, often from the north (*e.g.*, FLT3, FLT4, FLT8) (Fig. 5). Similarly, high altitude air masses associated with greater diversity (more OTUs) descended from greater heights over time until reaching the sampling location. Predictive forward air parcel trajectories from flights at the same location indicate that the short-term transport potential for fungi in these air masses is primarily local or regional (SEUS) but may extend beyond these areas (Figs. S5–S7). Most animal endophytes, dung saprobes, ectomycorrhizal fungi, and soil saprobes were detected at lower altitudes. Although leaf litter saprotrophs and parasites represent a minor contribution, they were largely present in high-altitude samples (Fig. 6).

**Figure 5:**
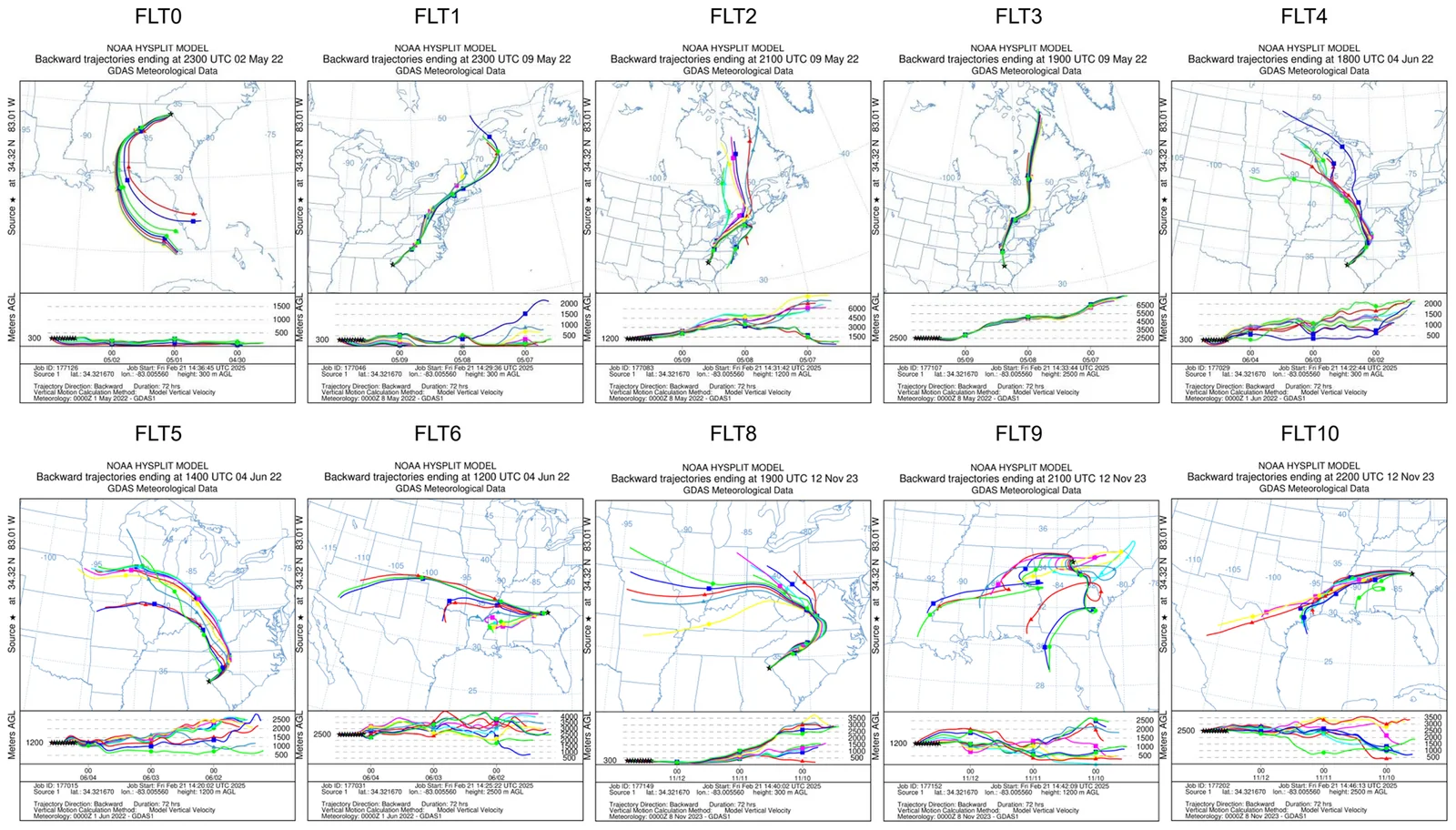
Atmospheric short term (72 h) travel trajectories of air masses carrying fungi sampled on each flight in Georgia. Air mass origins for each altitude were based on modelling 10-day backward trajectories using the NOAA Hybrid Single-Particle Lagrangian Integrated Trajectory (HYSPLIT) model developed by the NOAA Air Resources Laboratory. Beginning from the nearest hour to the start of sampling flight, a new trajectory was initiated every hour (maximum 10 trajectories) to derive a quantitative view of transport history. Label interval: 24 h.

**Figure 6:**
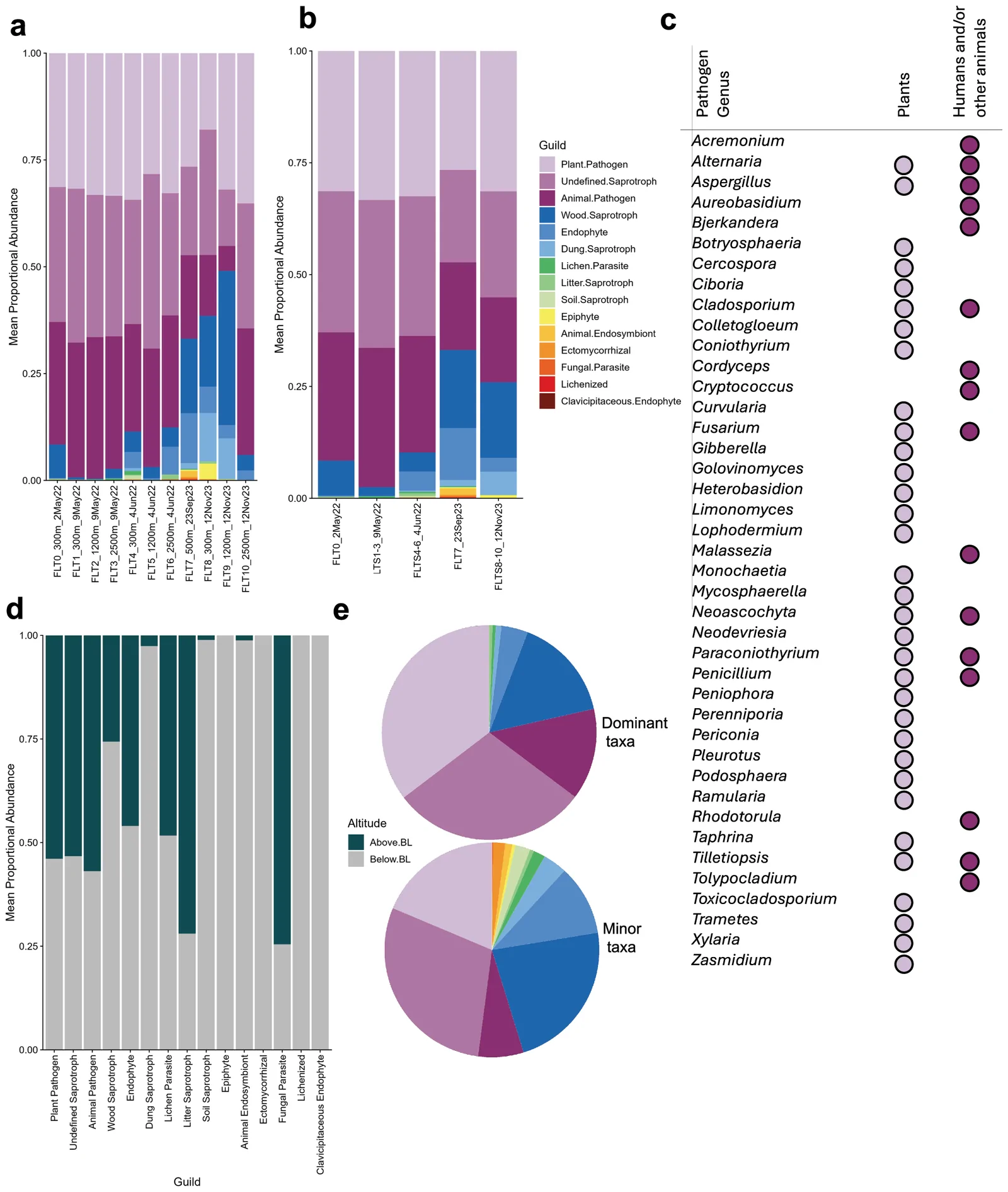
Distribution of functional trophic guilds of atmospheric fungi recovered by aircraft. Trophic guild assignments (A–E) and mean proportional abundance for airborne fungi recovered on all flights and altitudes (A), on flights grouped by date (B), by genus (C), above or below the atmospheric boundary layer (BL) at 2,500 m or ≤1,200 m AGL, respectively (D), and weighted prevalence of OTU richness for dominant (*n* = 23) and minor (*n* = 98) OTUs assigned to fungal functional guilds (E). (E) Weighted prevalence of all taxa can be found in Fig. S12. Guilds were annotated by FUNGuild (Nguyen et al., 2016), 121 of 166 OTUs were assigned to guilds, and all assignments with confidence levels of “probable” or “highly probable”, including those to multiple functional guilds, were retained in the analysis (Methods) (Abrego et al., 2024; Wagner et al., 2022). Putative pathogenic genera of plants and humans or other animals, including opportunistic pathogens and zoophilic (entomopathogenic) fungi, are shown as a circle in (C), and pathogenic taxa unidentifiable to genus level are unlisted. Putative pathogenic genera were determined through FUNGuild and references (Afshan et al., 2020; Chowdhary et al., 2014; Garcia-Aroca et al., 2021; Godfrey et al., 2018; Liu, Zhang & Chen, 2023; Martino et al., 2024). Animal refers to eukaryotic species, including vertebrates, birds, insects, arthropods, nematodes, and other fungi.

### Environmental and climatic influences on atmospheric mycobiome dynamics

A total of 121 of 166 OTUs were assigned to an ecological role according to FUNGuild and FungalTraits, revealing filamentous fungi, yeasts, and molds in the dataset with clavarioid, corticioid, gasteroid, microfungus, pezizoid, polyporoid, and xylarioid morphologies (Tables S2.7–S2.8). The weighted prevalence of all taxa assigned to guilds in the atmospheric mycobiome is shown in Fig. S10. Atmospheric fungal assemblages may be modulated at the genus and species levels by season, and higher level taxonomies (phylum and class) are less affected but still shift (Fig. 3, Figs. S11–S15; Tables S2.4–S2.6). Dothideomycetes, the most ecologically diverse class of AMC fungi including economically important plant pathogens, were enriched in spring and autumn (Fig. 6). BMC fungi were enriched in spring.

PCA reduced the environmental dataset into three major orthogonal gradients explaining 89.3% of total environmental variance (Fig. S3). PC1 (47.2%) represents a thermodynamic gradient loading strongly on temperature, dewpoint, atmospheric density, pressure, and cloud cover. PC2 (31.0%) represents an atmospheric transport/vegetation productivity gradient loading strongly on wind, NDVI, and other season-associated variables. PC3 (11.1%) primarily represents a vertical gradient loading on altitude and atmospheric boundary layer variables. Principal components regression on environmental gradients summarized by the first three PC axes collectively explained 86% of the variation in OTU richness in the full (all flights) dataset, suggesting sufficient explanatory power despite modest sample size (*R*^2^ = 0.860, adjusted *R*^2^ = 0.800; *F* = 14.32, *p* = 0.0023) (Fig. S3). Only PC2 was significantly associated with OTU richness (β = −10.48, *p* = 0.00036), indicating that fungal OTU richness declined along the environmental gradient represented by PC2 (wind and seasonal productivity). Airborne fungal diversity declines along an environmental gradient represented largely by increased seasonal vegetation productivity (NDVI) and atmospheric transport (wind) dynamics (and/or correlated factors). PC1 and PC3 were not significant gradients of OTU richness (Fig. S3).

Fungal community assemblages are seasonally distinct (Fig. 7, Figs. S8–S9). When the subset of environmental variables identified by PCA as contributing most strongly to differences among flights was used to investigate airborne fungal community composition, NMDS based on Bray–Curtis dissimilarities produced a reliable ordination of community relationships for all flights (stress = 0.083) (Fig. 7) and when FLT7 was excluded (stress = 0.074) (Fig. S8). Airborne fungal community composition was strongly associated with NDVI, which was the only significant environmental vector fitted to the ordination explaining 60.9% of the dissimilarity in the full dataset (*envfit*: *R*^2^ = 0.609, *p* = 0.027) (Fig. 7). A Mantel test showed a positive but non-significant association between NDVI and Bray–Curtis community dissimilarity (Mantel *r* = 0.2457, *p* = 0.073). Temperature (*R*^2^ = 0.210, *p* = 0.400), windspeed (*R*^2^ = 0.217, *p* = 0.393), and altitude (*R*^2^ = 0.055, *p* = 0.795) were not significantly associated with community composition. When FLT7 was excluded, NDVI explained 65.7% of the variation in ordination space (*envfit*: *R*^2^ = 0.6575, *p* = 0.024) with a slightly stronger association between NDVI and community dissimilarity (Mantel *r* = 0.3014, *p* = 0.050) (Fig. S8). No other focal predictors were significant (including or excluding FLT7). Collectively, these results suggest that seasonal vegetation productivity (*i.e.,* NDVI) can strongly influence airborne fungal community turnover, whereas temperature, wind, and altitude have comparatively weaker independent effects and additional unmeasured factors likely contribute to the remaining variation.

**Figure 7:**
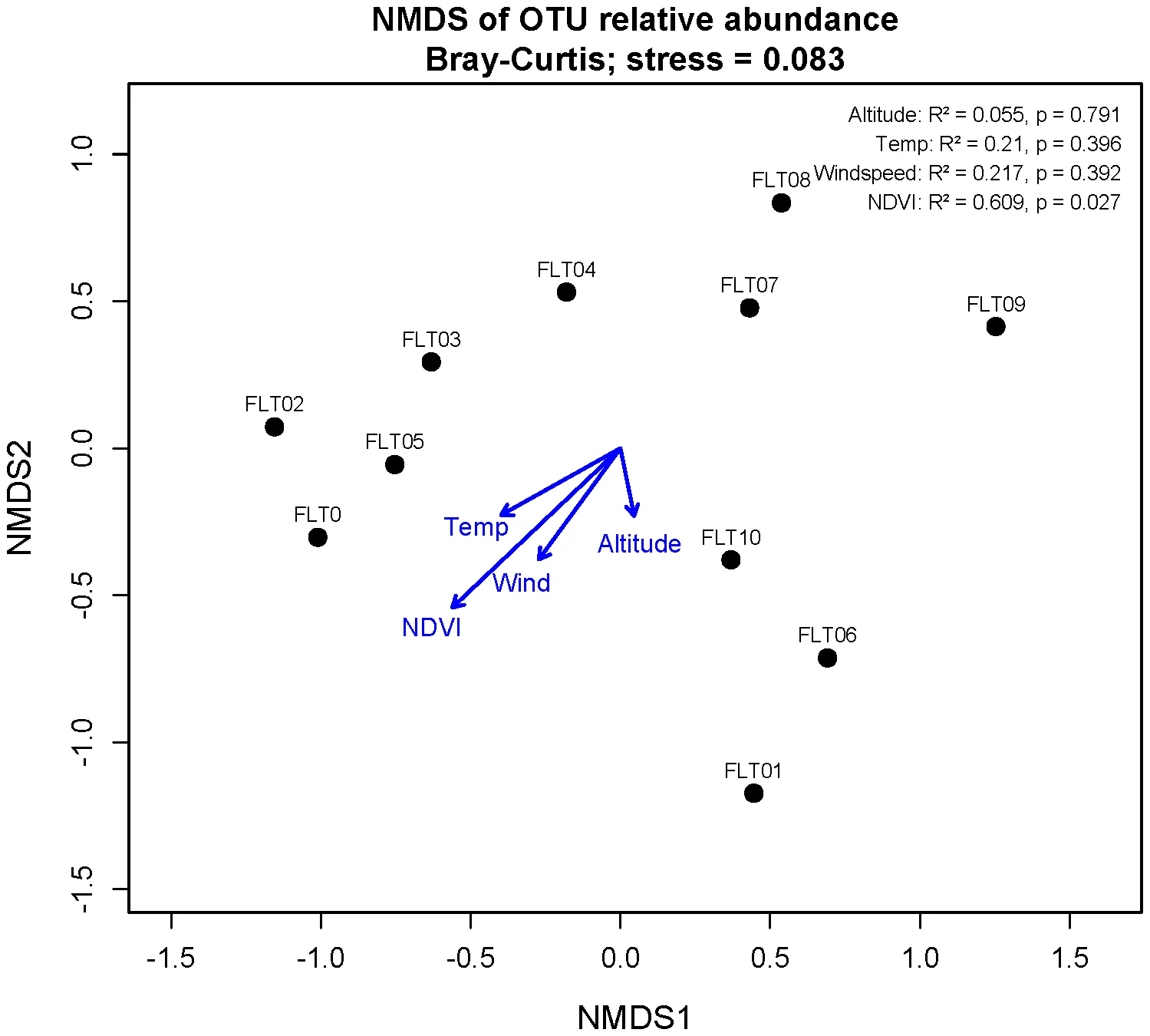
Ordination analysis of fungi community composition in the atmospheric mycobiome. Bray–Curtis Nonmetric Multidimensional Scaling (NMDS) ordination analysis on OTU community composition of airborne fungi on 11 aircraft survey flights. Environmental vector fitting of altitude, vegetation index NDVI, temperature, and windspeed was conducted *post hoc* using *EnvFit* in R. R^2^ values show the proportion of deviance in the ordination space explained by the focal environmental covariate.

Simple models, considered more appropriate for this limited dataset than more complex mixed models, were used to examine atmospheric mycodiversity (taxon richness and trophic guild richness) based on the candidate focal environmental predictors. Guild OTU richness for dominant and minor OTUs separately was also screened against these environmental variables.

The strongest overall multivariate regression model for taxon richness was the seasonal model (incorporating NDVI) for all flights, explaining 74.3% of the variance in airborne fungi OTU richness (*R*^2^ = 0.743; adjusted *R*^2^ = 0.572; *F* = 4.346, *p* = 0.0546); no other least-squares regression model combinations approached significance whether sample FLT7 was included or not (Tables S1.6–S1.7). NDVI was the strongest predictor of taxon richness in the seasonal model for all flights, indicating that taxon richness declined with increasing NDVI (β = −3.069, *p* = 0.0719); after accounting for date in the mixed-effect sensitivity analysis, the effect became significant (*p* = 0.013). Temperature became marginally significant (*p* = 0.0995) when date was included as a random effect.

When overall trophic guild richness was analyzed, significant seasonal models were obtained for undefined saprotrophs ((*R*^2^ = 0.755, adjusted *R*^2^ = 0.592, *F* = 4.630, *p* = 0.048), wood saprotrophs (*R*^2^ = 0.800, adjusted *R*^2^ = 0.667, *F* = 6.002, *p* = 0.027), and endophytes (*R*^2^ = 0.759, adjusted *R*^2^ = 0.598, *F* = 4.726, *p* = 0.046) (Table S1.8). Significant predictor effects were restricted to negative associations between NDVI and guild richness for undefined saprotrophs (β = −3.945, *p* = 0.048) and wood saprotrophs (β = −5.063, *p* = 0.038). Although no individual predictors were significant in the endophytes model, NDVI approached significance and was negatively associated with guild richness (β = −4.027, *p* = 0.078).

For guild richness of dominant OTUs collected on all flights, the best-fit statistically supported models were seasonal for animal pathogens (*R*^2^ = 0.764, adjusted *R*^2^ = 0.607, *F* = 4.866, *p* = 0.043) and dung saprotrophs (*R*^2^ = 0.988, adjusted *R*^2^ = 0.980, *F* = 121.9, *p* < 0.0001), and endophytes approached significance (*R*^2^ = 0.742, adjusted *R*^2^ = 0.571, *F* = 4.324, *p* = 0.055) (Table S1.8). Animal pathogen richness was negatively associated with NDVI (β = −1.509, *p* = 0.009) and positively associated with windspeed (β = 0.088, *p* = 0.044), whereas dung saprotroph richness was negatively associated with NDVI (β = −2.439, *p* < 0.0001) and temperature (β = −0.0156, *p* = 0.006). Dominant OTU richness for classified endophytes was negatively associated with NDVI (β = −5.315, *p* = 0.0108) and positively associated with windspeed (β = 0.352, *p* = 0.0324), while the seasonal animal pathogen model remained the best-supported model but was no longer significant (*p* = 0.074). The only significant primary model for dominant OTU richness was for dung saprotrophs (*R*^2^ = 0.770, adjusted *R*^2^ = 0.0.671, *F* = 7.799, *p* = 0.012), where windspeed was significantly negatively associated (β = −0.149, *p* = 0.014) and negative temperature effects approached significance (β = −0.033, *p* = 0.051).

For trophic guild richness of minor (rare) OTUs, the best statistically supported models were seasonal models for plant pathogens (*R*^2^ = 0.790, adjusted *R*^2^ = 0.650, *F* = 5.644, *p* = 0.031), undefined saprotrophs (*R*^2^ = 0.854, adjusted *R*^2^ = 0.756, *F* = 8.745, *p* = 0.011), wood saprotrophs (*R*^2^ = 0.845, adjusted *R*^2^ = 0.741, *F* = 8.171, *p* = 0.013), and endophytes (*R*^2^ = 0.809, adjusted *R*^2^ = 0.681, *F* = 6.333, *p* = 0.024) (Table S1.8). Significant predictor effects within these models were limited to negative NDVI relationships for undefined saprotrophs (β = −4.686, *p* = 0.045) and wood saprotrophs (β = −6.220, *p* = 0.021), whereas no individual predictors were significant in the seasonal model for plant pathogens or endophytes despite significant overall models (Table S1.8). Overall primary models for all flights were also significant for plant pathogens (*R*^2^ = 0.659, adjusted *R*^2^ = 0.512, *F* = 4.503, *p* = 0.046) and undefined saprotrophs (*R*^2^ = 0.698, adjusted *R*^2^ = 0.569, *F* = 5.394, *p* = 0.031) with weak associations with windspeed (β = −0.425, *p* = 0.016 and β = −0.252, *p* = 0.041, respectively).

When FLT7 was excluded, none of the total OTU (all) guild richness models remained significant. The overall primary model provided the best fit for dominant OTUs classified as plant pathogens (*R*^2^ = 0.733, adjusted *R*^2^ = 0.599, *F* = 5.486, *p* = 0.037), with a significant negative windspeed association (β = −0.251, *p* = 0.0097) and weak negative temperature relationship (β = −0.025, *p* = 0.088) (Table S1.9). The primary model was also the best-supported model for minor OTUs classified as endophytes (*R*^2^ = 0.717, adjusted *R*^2^ = 0.576, *F* = 5.078, *p* = 0.044), although no individual predictor was significant. The seasonal model provided the best fit for dominant OTUs classified as dung saprotrophs (*R*^2^ = 1.000, adjusted *R*^2^ = 1.000, *p* < 0.0001) and the corresponding primary model was also significant (*R*^2^ = 0.722, adjusted *R*^2^ = 0.583, *p* = 0.042) although dung saprotrophs were sparsely represented in the dataset and showed evidence of autocorrelation (Ljung–Box test, *p* = 0.0196) (Table S1.9). Interestingly, guilds composed of dominant OTUs were more often best explained by the primary model (Altitude + Temperature + Windspeed) rather than the seasonal model (+ NDVI), which suggests that dominant taxa, more abundant members of the airborne fungal community, may be more widespread, continuously available fungi that are less constrained by vegetation biomass and respond more strongly to aerosolization and dispersal conditions.

Random intercept variance for date collapsed to zero in mixed-effects seasonal sensitivity analyses for both taxon and trophic guild richness (Tables S1.6–S1.9), indicating negligible effects of sampling date. No evidence of temporal autocorrelation was detected in significant seasonal models or significant primary models apart from dominant OTUs classified as dung saprotrophs (Tables S1.6–S1.9).

In this study, airborne fungal diversity and community turnover are inversely related to the vegetative index NDVI (Fig. 8). Vegetative decay and turnover coincided with elevated diversity in airborne fungi. Overall fungal richness was higher on days with lower UV, less green vegetation (more decay), later in the year, and in GA on cloudier days. Overall fungal richness was lower on windy, gusty days, and in GA when solar radiation and vegetation productivity were higher. Conversely, relative abundance increased on warmer, humid, windy days. Less common leaf litter saprotrophs were in higher altitude samples, as were parasites (Fig. 6, Figs. S11, S13–S15). Plant pathogens were more diverse in warmer conditions (Fig. 8). As samples become more dissimilar in vegetation productivity or decay, they also become more dissimilar in terms of fungal community composition. These associations were particularly strong for a few dominant OTUs (OTU33, *Alternaria alternata*, OTU2, *Ascomycota*, and OTU7, *Cladosporium* spp.) and among many minor (rare) or transient OTUs (Fig. S12), suggesting that the minor or transient component of the airborne fungal community may be sensitive to environmental filtering. These findings remained robust whether FLT7 was included or excluded.

**Figure 8:**
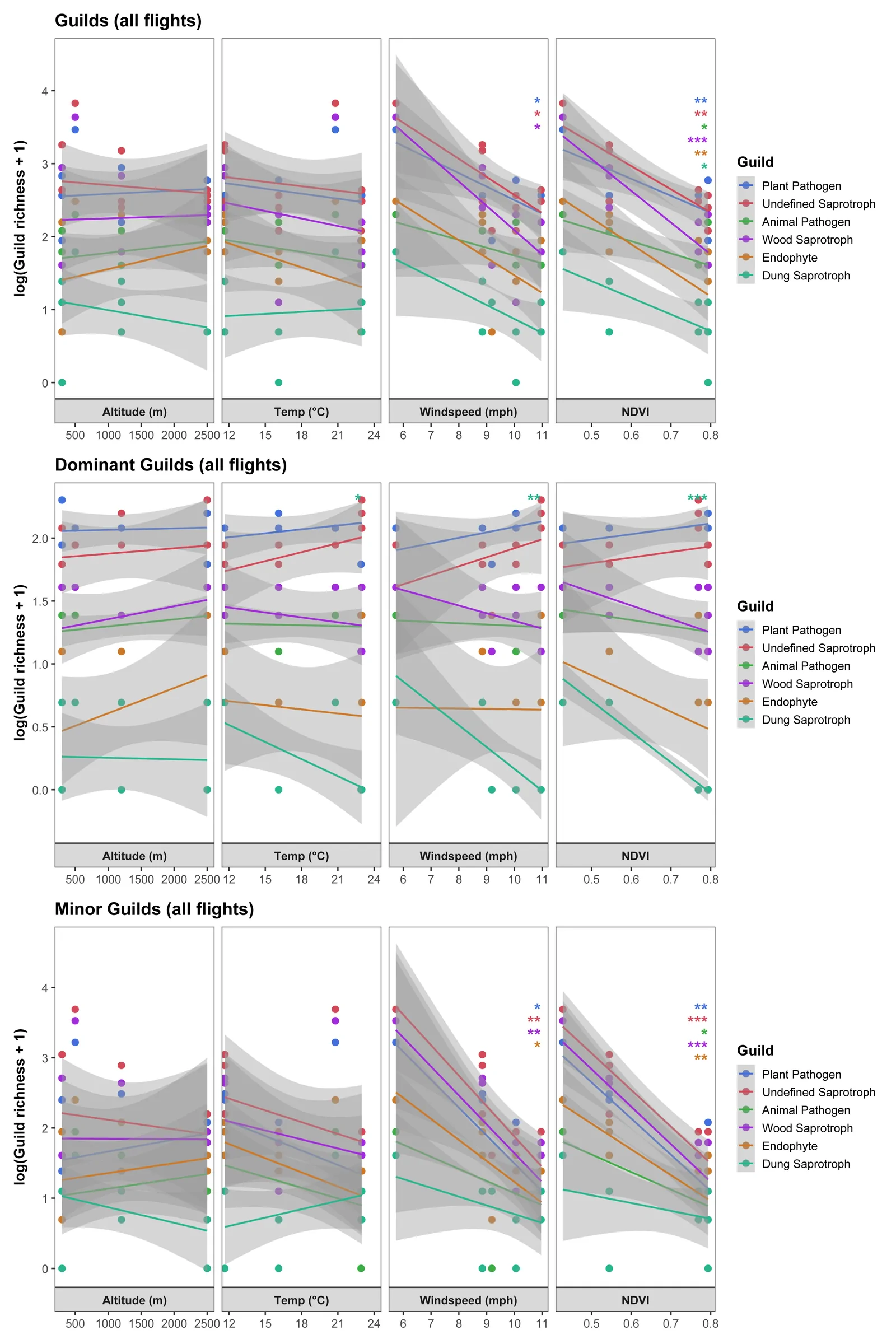
Environmental and seasonal variation in atmospheric fungal diversity on 11 aircraft survey flights. Airborne fungi trophic guild richness response to environmental predictors for dominant, minor, and all taxa (OTUs). Guilds with ≤3 nonzero flights containing total, dominant, and minor OTUs are shown. Panels show bivariate relationships between guild richness [log(guild richness + 1)] and focal environmental predictors (Altitude, Temperature, Windspeed, and normalized difference vegetation indices, NDVI). Points represent observed log-transformed guild richness values (number of OTUs assigned to each guild per sample); solid lines represent ordinary least-squares linear regressions with 95% confidence intervals. Significance annotations indicate results of separate univariate regressions fitted independently for each guild-predictor combination (* *p* < 0.05, ** *p* < 0.01, *** *p* < 0.001). Regression of fungal guild-assigned dominant and minor OTU richness across all flights. Lower NDVI values reflect lower photosynthetic activity. The same analyses excluding sample FLT7 in SC (thus, for GA only) can be found in the Supporting Information.

## Discussion

Advances in environmental air monitoring technology and aeronautical capabilities can be leveraged to strengthen ground-based surveillance. A multifaceted view of the atmospheric mycobiome was achieved analyzing DNA filtered with a standardized decontaminated high-integrity vacuum sampling system on aircraft. Plant pathogens and general saprobes, including wood decomposers, dominate all heights of the sampled atmospheric mycobiome. Rich in free-living filamentous and host-dependent fungi, the samples also include fungi that contribute to ecosystem health. Fungal DNA was detected in air masses predicted to disperse across distances exceeding previous estimates by more than fivefold (Rodó et al., 2024). Total airborne fungal DNA is relevant for ecosystem monitoring and bioaerosol dynamics, highlighting critical implications for ecosystem health, food security, and biodiversity, emphasizing the importance of biological monitoring of the atmosphere.

Airborne fungi are widely distributed vertically in the atmosphere, implying effective circulation or resuspension. Overall, the number of dominant OTUs remained relatively stable across flights, with a core set of fungi observed throughout the sampling period, consistent with previous findings (Tipton et al., 2019). However, minor OTU richness was more variable, with a larger proportion of minor OTUs at lower altitudes. Patterns of sinking and ascending air reveal potential dispersal paths. Together, these results suggest that atmospheric circulation dynamics, not altitude alone, contribute to mycobiome variability.

Many of the fungi show associations with close origins in local atmospheric conditions, but transport over long distances cannot be ruled out. In general, atmospheric fungi reflect recruitment from underlying habitats, including conifer and hardwood stands and agricultural biota. *Lophodermium australe* is associated with healthy foliage of pines in this region (Oono et al., 2014) and presumably of local origin, but did not appear in samples below the boundary layer, suggesting spatial heterogeneity, selective sorting driven by physical characteristics, or stochastic variation in detection. *Baeospora myosura* or “conifer cone cap” was recovered on all autumn flights, and produces fruit bodies on pine and spruce cones that grow higher in the tree canopy (Hobbie et al., 2014). *Heterobasidion irregulare,* a plant pathogen that can cause conifer root rot, an economically important disease with devastating consequences for pine tree plantations in the US, was also detected (Ohno et al., 2018). Other pine-associated fungi recovered were *Alutaceodontia alucea* and *Rescinium friable*. Of the unique taxa detected at 1,200 m, *Xylaria liquidambaris* may be recruited locally given its exclusive association with the fruit of American sweetgum, a deciduous hardwood common in the region. Other fungal species recovered associated with local deciduous hardwoods include *Ramularia vizellae, Trametes cubensis*, *Monochaetia dimorphospora*, *Fonsecazyma* (*Cryptococcus*) *mujuensis*, *Elmerina caryae*, *Lentinellus ursinus*, *Mycoacia uda*, *Gloeoporus dichrous*, *Pholiota polychroa, Trametes* (*Lenzites*) *betulina*, and *Daedaleopsis confragosa*, some with anticancer, antihypertension, or antimicrobial properties. Several yeast species recovered, including *Rhodotorula glutinis, A. pullulans*, and *C.* (*Papiliotrema*) *laurentii,* have been isolated from tree exudate (Buzzini, Lachance & Yurkov, 2017). Fungi enriched on the same sampling flights as *Cupriavidus*, *Methylobacterium*, and *Pseudomonas* (9-May) and *Bradyrhizobium*, *Kineosporia*, *Methylobacterium* and *Streptococcus* (4-June) include *Cladosporium* and *Neoascochyta*, suggesting possible interactions among *Cladosporium* and/or *Neoascochyta* with these co-occurring airborne bacteria (Métris & Métris, 2023).

A considerable proportion of the fungi detected in this study are pathogens (44.57%, 41/92 genera). At least 34.78% are phytopathogens, including several of the top 10 fungal pathogens in molecular plant pathology (Dean et al., 2012) or listed as fungal priority pathogens for systematic surveillance (WHO, 2022), and over 10% are known pathogens of animals including humans. Genera responsible for economically destructive diseases such as powdery mildew were present in the air samples. *Alternaria alternata*, which causes leaf spot on plants and respiratory infections in immunocompromised humans, was found in most samples. Several yeast-like microfungi recovered occur in animals, including *Aureobasidium*, *Tilletiopsis*, *Malassezia*, *Rhodotorula*, and *Cryptococcus*. *Malassezia sympodialis* is part of the healthy human microbiome and linked to allergies (Vitte et al., 2022). Along with *Aspergillus versicolor*, *M. sympodialis* can survive in the chicken microbiome (Robinson et al., 2022), and *Hannaella sinensis* has been reported in ceca of commercial poultry (Byrd, Caldwell & Nisbet, 2017). *Rhodotorula glutinis* can cause fungemia and dermatitis. While unconfirmed, some airborne fungi detected in this study may be associated with human, livestock, or poultry DNA recovered from the same filters (Métris & Métris, 2023). *Acremonium* includes saprobes and pathogens of plants, insects, and infrequently humans (Perdomo et al., 2011). *Cryptococcus laurentii*, linked to bird feces in the eastern US (Filion, Kidd & Aguirre, 2006) and human disease (Molina-Leyva et al., 2013), and *Cordyceps polyarthra*, often found on winged insects, were also recovered from the air samples.

This study explored niche-driven assembly in airborne fungi, expanding our understanding of microbial community processes that have primarily focused on bacteria (Liao et al., 2016). Detection of leaf-litter associated fungi more commonly at higher altitudes above the ground suggests that aerosolization of leaf material may facilitate vertical transport and (re)suspension, highlighting that community composition is influenced by dispersal ability and seasonal or climate dynamics (Tipton et al., 2019). Local meteorological conditions, shape, density, and desiccation have been shown in model simulations to influence aerial dispersal capabilities in airborne microorganisms (Galbán et al., 2021). Beyond spatiotemporal variation and dynamic changes in the abundance of airborne microorganisms, as well as their relationships with atmospheric conditions, characteristics of the biological particles themselves, such as variation in size, density, and desiccation capability, may also influence dispersal. In particular, non-spherical microorganisms with very small equivalent spherical diameters, reduced density, and increased dehydration can remain airborne for more than 20 h and be transported over long distances (Galbán et al., 2021). Roles of particulate matter characteristics—such as aerodynamic diameter and buoyancy—in airborne fungal transport and their impact on biogeographical patterns were beyond the scope of this study but merit further investigation for their potential roles in zonal separation.

Seasonal shifts influence fungal proliferation, sporulation, dispersal, and heterogeneity (Bowers et al., 2013; Cáliz et al., 2018; Nageen, Wang & Pecoraro, 2023). In spring, BMC fungi were enriched, particularly Agaricomycetes, soil- and wood-inhabiting fungi often reported in the air (Egidi et al., 2019). *Lophodermium australe*, a pine-associated “needle cast” endophyte, was detected in spring but not in autumn. This fungus produces fruiting bodies in spring, leading to the premature shedding of pine needles. Autumn samples were enriched with Eurotiomycetes and mold-type fungi linked to ice-cloud nucleation. In this study, *Heterobasidion irregulare* was detected exclusively in autumn, when spores germinate and infection typically begins.

In this study, aircraft-captured fungi varied seasonally with local habitat productivity, which may represent an important driver of airborne fungi diversity and community turnover. The plant pathogen guild in particular increased during periods with lush host vegetation (Lymperopoulou, Adams & Lindow, 2016). Wood and leaf decomposers peaked when productivity declined, aligning with increased spore concentrations post-harvest as crops decay (Grinn-Gofroń & Bosiacka, 2015). Increased fungal DNA and diversity in autumn has been reported elsewhere (Abrego et al., 2024; Banchi et al., 2020; Fröhlich-Nowoisky et al., 2009; Maki et al., 2023; Núñez et al., 2021; Tignat-Perrier et al., 2020). Putative phytopathogens were more abundant during periods of increased temperature, solar energy, UV, and winds, when vegetation was greener in spring, suggesting increased surface warming and convection may lift fungi from productive vegetation into the atmosphere. Leaf litter saprotrophs were proportionally more abundant above the boundary layer than below it, fungal types that may be more easily transported and resuspended into the atmosphere—or less easily deposited therefrom. Foliar fungi from tree leaves contribute to atmospheric mycodiversity, which may present a creative opportunity to assess forest management if old growth forests contain higher mycodiversity compared to trees in nurseries (Ganley & Newcombe, 2006). It is possible that wind influences patterns of certain trophic guilds. Although the aircraft flight patterns minimized wind effects at altitude, surface wind speed presumably impacts fungal aerosolization, increasing detection of some guilds (*e.g.*, dominant animal pathogens and endophytes) while reducing detection of others.

Marked increases in dung saprobes and wood-rotting fungi coincide with lower vegetation productivity (*i.e.,* more decay) in autumn, suggesting that periods of reduced photosynthetic activity foster greater fungal diversity. This pattern may be driven by increased vegetative decay and turnover, as well as seasonal soil and leaf litter cycling. Both anthropogenic (*e.g.*, agricultural) and natural disturbances seem to influence airborne fungal communities. The fungi closely associated with tree species overflown in this study point to ecological mechanisms driving airborne DNA from dominant mycoflora (Smith et al., 2012). Vertical profiles of airborne fungi suggest effective mixing, presumably because these flights were conducted through an abundance of boundary layer air enriched with local sources compared to global scale tropospheric air. Accordingly, the data support the findings of others who have shown that short term variability in airborne microbial community composition (Bertolini et al., 2013; Gusareva et al., 2019) can be quickly homogenized and redistributed among areas (Núñez et al., 2021).

Primer efficiencies, database limitations, and sequencing depth can affect diversity estimates, OTU variation, and guild distribution across flights which may reflect a combination of stochastic effects, mixing of air masses, and enrichment of traits enhancing survival in the atmosphere (Starmer & Lachance, 2011). Filter pore size, for example, could influence which biological particles are retained and therefore which fungal taxa are detected, potentially impacting microbial profiles recovered. While others have found little or no evidence that 0.22 and 0.45 µm filters differentially affect eukaryotic community composition recovered by metabarcoding (Clare et al., 2022), conclusions drawn from aquatic studies or studies targeting different taxa may not be directly applicable to airborne fungal communities, highlighting the need for future fungal aerobiome studies that directly compare multiple possible sources of methodological bias. It should also be considered that the DNA metabarcoding methods used here sequence DNA that is/was associated with living organisms but cannot confirm whether it was from live, metabolically active, or infectious cells at the time of collection. Nevertheless, airborne microbial particles can influence atmospheric processes whether they are live or dead biomass (Bhargavi & Savitha, 2014). Further, this study filtered samples (*i.e.,* using dereplication filters and abundance as well as read count thresholds) to reduce false negatives across samples which would inflate diversity estimates, although it is noted that this can potentially lead to underreported diversity (underestimating rare taxa) or compositional biases. Controlling for variation in sequencing depth through data transformation techniques (*e.g.*, log-transformation and re-scaling) allows more biologically meaningful information contained in samples sequenced at lower depth, particularly in highly resource-intensive studies, to inform broad patterns robust to downsampling and can serve as a roadmap for future atmospheric biodiversity studies facing similar constraints.

We found that the fungal content of air samples collected is primarily derived from local or regional sources with inferred short-term transport impacts of similar scale. Several environmental variables were identified as potential drivers of fungal richness and community composition in this study, including vegetation productivity, temperature, and wind speed. Together, these findings highlight the importance of environmental conditions in structuring airborne fungal communities, though there are certainly other variables not the focus of this study that may also contribute to observed patterns. Further aircraft sampling across broader spatial and temporal scales with increased and balanced representation will be necessary to evaluate the robustness and generality of these influences.

Airborne fungal diversity in this study exceeds some reports from the western US (Smith et al., 2012) and Europe (Sánchez-Parra et al., 2021), though the terrestrial origins remain consistent. An important consideration between studies is that sampling methods and site selection impact comparisons across studies. The instrumentation used here and benchmarked elsewhere (Métris & Métris, 2023) captures fungi of various sizes independent of settling velocities, in contrast to traditional spore traps (Woo et al., 2018), making it valuable for future large-scale studies in different regions. The dataset used in this study, derived from 11 flights sampling multiple altitudes with duplicate filters per flight (22 atmospheric samples) is consistent with typical aircraft survey datasets which often prioritize broad spatial coverage and high information content over volume *e.g.*, 2–4 multi-altitude flights (16 atmospheric samples) (Jaing et al., 2020; Smith et al., 2018), 5 flights (up to 48 samples) (Drautz-Moses et al., 2022), 9 flights (with duplicate samples) (DeLeon-Rodriguez et al., 2013), and 10 flights (22 samples) (Rodó et al., 2024). However, long-term, continuous, and worldwide sampling adding to this foundation is encouraged based on these findings and will be necessary to understand global drivers modulating fungi in the atmosphere. High-throughput targeted amplicon and shotgun sequencing are widely applied and affordable culture-free approaches for characterizing microbiomes, their putative pathogenicity and ecological roles in low-biomass atmospheric environments, although potential for air monitoring by nanopore sequencing (Reska et al., 2024) may soon provide the ability to confirm and characterize pathogenic variants.

## Conclusions

This study introduces a multidisciplinary approach toward future advances in aerobiology (Amato et al., 2023). Expanding ground-based monitoring to higher altitudes with broader coverage can enable early detection of bioaerosols of interest and facilitate rapid response. Such monitoring supports proactive measures to protect crops, livestock, and ecosystems. This work also demonstrates how environmental data could be applied in future for broader aerobiological insights to aid in predicting changes driven by human activities, natural events, and emerging pathogens, strengthening our capacity for future biological surveillance.

## Supplemental Information

10.7717/peerj.21746/supp-1 Supplemental Information 1 Supporting Information

10.7717/peerj.21746/supp-2 Supplemental Information 2 R code

10.7717/peerj.21746/supp-3 Supplemental Information 3 Scripts for SAS/JMP plots

## Additional Information and Declarations

### Data Availability

The following information was supplied regarding data availability: The code and scripts are available at FigShare: - https://doi.org/10.6084/m9.figshare.30037891
Analyses conducted in R (R Core Team, 2024) used the packages ggplot2, reshape2, dplyr, hrbrthemes, vegan, svglite, ecodist, and geosphere, as outlined in and available at GitHub: - https://jkzorz.github.io. The raw sequence data are available through NCBI SRA under BioProject PRJNA1169604: - http://www.ncbi.nlm.nih.gov/bioproject/1169604.
