# Supplementary material for: Fungus above us: exploring the atmospheric mycobiome by aircraft
Source: PeerJ. 2026 Sep 30;14:e21746. doi: 10.7717/peerj.21746 (PMC13633693; doi:10.7717/peerj.21746)
Supplement: Supplementary file 1 [file peerj-14-21746-s001.docx]

*PeerJ*

Supporting Information for

**Fungus above us: exploring the atmospheric mycobiome by aircraft**

K. L. Métris^1,2,3^ and J. M. Métris^3^

^1^Department of Genetics and Biochemistry, Clemson University, Clemson, SC 29634 USA.

^2^School of Health Research, Clemson University, Clemson, SC 29634 USA.

^3^Airborne Science LLC, Clemson, SC 29631 USA.

**Contents of this file**

Figures S1 to S18

Tables S1.1 to S1.9

**Additional Supporting Information (Files uploaded separately)**

Dataset S2

R code.R

Scripts.SAS

as DOI: 10.6084/m9.figshare.30037891

**Introduction**

This file includes the primary supporting information for the ITS2 metabarcoding, meteorological, and environmental datasets described in the paper.

**Figure S1.** Representative flight track paths for **a,** FLT0-6 GA in 2022; **b,** FLT8-10 GA in 2023 and **c,** FLT7 in SC in 2023. **a-c**, Map credit: © Google Landsat/Copernicus. **d,** flight perimeter around the Clemson University stadium with temporary flight restriction in red. Map credit: © Jeppesen ForeFlight.

**2-May-2022**

**a**

**d**

**12-Nov-2023**

**b**

**c**

**23-Sep-2023**

**Figure S2.** Rarefaction accumulation curve of sequences sampled (in thousands) and number of OTUs recovered by flight (FLT) before total sum scaling normalization to 10,000 reads. Rarefaction slopes highlighted in red (FLT1,FLT2) in particular suggest that for these two samples at shallow sequencing depths, additional OTUs (rare taxa) may be missed. The dataset was re-scaled to 10,000 reads.

**Figure S3. Multivariate principal components analysis (PCA) loadings, selection of focal predictors for analysis in the study, and regression of airborne fungi OTU richness on retained PC axes.** JMP was used to visualize all environmental variables with variation. All scripts used are provided in the Scripts.SAS file.
(**a**) Ranked environmental variable loadings for the first three principal components (PC1-PC3), with the relative contribution of each environmental predictor to the corresponding axis. Variables are ordered by the absolute magnitude of their loadings within each principal component (PC). (**b**) PCA summary biplots of PCs including all 17 environmental variables with variation separating flights. The length and direction of each vector indicate the strength and type (positive or negative) of the correlation between specific variables and the PCs. DA, density altitude; SLP, atmospheric sea level pressure; ABL, atmospheric boundary layer. (**c**) Overall model fit for the multiple regression of OTU richness against retained PCA axes. (**d**) Regression coefficient estimates (β), standard errors (SE), t-statistics, and p-values for individual PC predictors included in the PCA regression model. Positive coefficients indicate positive associations between the corresponding PC and OTU richness, whereas negative coefficients indicate inverse associations. Statistical significance is indicated: † *p* < 0.10; * *p* < 0.05; ** *p* < 0.01; *** *p* < 0.001.

(**a**) **PCA Eigenvector values**

| **Rank** | **PC1** **(47.23%)** | **Loading** | **PC2 (31.03%)** | **Loading** | **PC3 (11.11%)** | **Loading** |
| --- | --- | --- | --- | --- | --- | --- |
| 1 | DA | 0.3398 | Windspeed | 0.4093 | ABL | 0.6291 |
| 2 | Temp_DailyMean | 0.3359 | Windgust | 0.4061 | Altitude | 0.5986 |
| 3 | Dewpoint | 0.3296 | NDVI | 0.3556 | Temp_Altitude | 0.2733 |
| 4 | SLP | 0.3269 | Season | 0.2963 | SLP | 0.1626 |
| 5 | CloudCover | 0.2988 | UVIndex | 0.2963 | Solar_Energy | 0.1614 |
| 6 | DayofYear | 0.2652 | Solar_Energy | 0.2950 | Solar_Radiation | 0.1593 |
| 7 | Temp_Altitude | 0.2530 | Solar_Radiation | 0.2891 | CloudCover | 0.1330 |
| 8 | Humidity | 0.2487 | DayofYear | 0.2358 | Dewpoint | 0.1234 |
| 9 | UVIndex | 0.2443 | CloudCover | 0.1929 | Temp_DailyMean | 0.1200 |
| 10 | Season | 0.2443 | Altitude | 0.1714 | Humidity | 0.1188 |
| 11 | Solar_Radiation | 0.2143 | Temp_Altitude | 0.1712 | Windgust | 0.0998 |
| 12 | Solar_Energy | 0.2112 | ABL | 0.1557 | DA | 0.0928 |
| 13 | NDVI | 0.1820 | Humidity | 0.1080 | NDVI | 0.0794 |
| 14 | Altitude | 0.1123 | DA | 0.0785 | Windspeed | 0.0673 |
| 15 | ABL | 0.0948 | SLP | 0.0300 | Season | 0.0310 |
| 16 | Windspeed | 0.0706 | Temp_DailyMean | 0.0236 | UVIndex | 0.0310 |
| 17 | Windgust | 0.0343 | Dewpoint | 0.0008 | DayofYear | 0.0240 |

***The four candidate focal predictors selected in this study are underlined*** *above.* Variance inflation factors (VIFs) calculated for each focal environmental predictor were all < 5: 1.156 (altitude), 1.401 (temperature), 3.878 (windspeed), and 4.318 (NDVI).

(**b**) **PCA summary plots**

(**c**) **Overall model performance statistics for the fitted PCA regression model *OTU Richness ~ PC1 + PC2 + PC3***

| **Statistic** | **Value** |  |
| --- | --- | --- |
| R² | 0.860 |  |
| Adjusted R² | 0.800 |  |
| Residual SE | 11.83 |  |
| F-statistic | 14.32 |  |
| F-stat df | 3, 7 |  |
| Model p | **0.0023** | ** |

(**d**) **Predictor coefficient estimates for PCA regression model**

| **Predictor** | **β Estimate** | **SE** | **t** | **p** |  |
| --- | --- | --- | --- | --- | --- |
| PC1 | -0.267 | 1.320 | -0.202 | 0.845 |  |
| PC2 | -10.481 | 1.628 | -6.437 | **<0.001** | *** |
| PC3 | 3.306 | 2.721 | 1.215 | 0.264 |  |

**Figure S4.** Atmospheric mycobiome fungal diversity metrics. Analyses performed in Namco Microbiome Explorer. **a**, Alpha diversity of fungal communities in this study. Shannon entropy and Simpson indices are non-linear; a Shannon index of x is not twice as diverse as a Shannon index of 2x. To account for this, Namco calculates the effective index as well, which corresponds to the number of equally abundant species that would yield the same index value. **b**, Beta diversity of fungal communities grouped by season. Principal Coordinates Analysis using Bray-Curtis Dissimilarity. Permutational multivariate analysis of variance (PERMANOVA) (p=0.015).

| **Flight** | **Shannon Entropy** | **Effective* Shannon Entropy** | **Simpson Index** | **Effective* Simpson Index** | **Richness** |
| --- | --- | --- | --- | --- | --- |
| FLT00 | 1.31 | 3.72 | 0.36 | 2.75 | 25 |
| FLT01 | 1.35 | 3.85 | 0.38 | 2.61 | 23 |
| FLT02 | 0.6 | 1.82 | 0.76 | 1.32 | 28 |
| FLT03 | 1.09 | 2.97 | 0.52 | 1.94 | 42 |
| FLT04 | 2.21 | 9.11 | 0.2 | 5.03 | 41 |
| FLT05 | 1.77 | 5.86 | 0.26 | 3.9 | 32 |
| FLT06 | 2.25 | 9.51 | 0.22 | 4.49 | 39 |
| FLT07 | 3.04 | 20.94 | 0.14 | 7.1 | 116 |
| FLT08 | 1.87 | 6.51 | 0.35 | 2.83 | 64 |
| FLT09 | 2.12 | 8.35 | 0.17 | 5.92 | 54 |
| FLT10 | 1.82 | 6.18 | 0.25 | 3.97 | 35 |

**Figure S5.** Future transport potential (short-term 72 hours) of air masses sampled on each flight at the same location in Georgia, with implications for airborne fungi. Predicted transport potential was based on modelling HYSPLIT 10-day forward trajectories. Beginning from the nearest hour to the start of the sampling flight, a new trajectory was initiated every hour (maximum 10 trajectories) in the NOAA Hybrid Single-Particle Lagrangian Integrated Trajectory (HYSPLIT) model developed by the NOAA Air Resources Laboratory. Label interval: 24 hours.

Three day trajectories:

**Figure S6.** Future transport potential (long-term 240 hours) of air masses sampled each date at the same location in Georgia, with implications for airborne fungi. Predicted transport potential was based on modelling HYSPLIT 10-day forward trajectories. Beginning from the nearest hour to the start of the sampling flight, a new trajectory was initiated every hour (maximum 10 trajectories) in the NOAA Hybrid Single-Particle Lagrangian Integrated Trajectory (HYSPLIT) model developed by the NOAA Air Resources Laboratory. Label interval: 24 hours.

10-day trajectories:

**Figure S7.** HYSPLIT trajectories of backward and forward transport potential for the air mass sampled in SC (FLT7). Predicted transport potential was based on modelling HYSPLIT 10-day forward or backward trajectories. Beginning from the nearest hour to the start of the sampling flight, a new trajectory was initiated every hour (maximum 10 trajectories) in the NOAA Hybrid Single-Particle Lagrangian Integrated Trajectory (HYSPLIT) model developed by the NOAA Air Resources Laboratory. Label interval: 24 hours.

10-day trajectories:

**Figure S8.** Environmental vector fitting (envfit) and Mantel test results associated with NMDS ordinations of fungal community composition based on Bray-Curtis dissimilarity matrices for all flights and excluding FLT7. NMDS vector coordinates (NMDS1 and NMDS2), coefficients of determination (R²), percentage of explained variation, Mantel correlation coefficients (r), and permutation-based p-values. Significance was assessed at α = 0.05. † p < 0.10; * p < 0.05; ** p < 0.01; *** p < 0.001.

(**a**) **NMDS environmental vector fitting (envfit) (All Flights)**

| **Environmental Variable** | **NMDS1** | **NMDS2** | **R²** | **% Explained** | **p** |  |
| --- | --- | --- | --- | --- | --- | --- |
| Altitude | 0.0462 | -0.2298 | 0.0549 | 5.4949 | 0.791 |  |
| Temperature | -0.3978 | -0.2269 | 0.2097 | 20.9734 | 0.396 |  |
| Windspeed | -0.2723 | -0.3781 | 0.2171 | 21.7148 | 0.392 |  |
| NDVI | -0.5611 | -0.5420 | 0.6086 | 60.8570 | **0.027** | * |

(**b**) **Mantel tests between Bray Curtis community dissimilarity and environmental distances (All Flights)**

| **Environmental Variable** | **Mantel r** | **p** |  |  |
| --- | --- | --- | --- | --- |
| Altitude | -0.2137 | 0.952 | |  |
| Temperature | -0.0381 | 0.567 |  |  |
| Windspeed | -0.0732 | 0.664 |  |  |
| NDVI | 0.2457 | 0.073 | † |  |

(**c**) **NMDS environmental vector fitting (envfit) for Georgia flights (Excluding FLT7)**

| **Environmental Variable** | **NMDS1** | **NMDS2** | **R²** | **% Explained** | **p** |  |
| --- | --- | --- | --- | --- | --- | --- |
| Altitude | 0.1051 | -0.2350 | 0.0663 | 6.6261 | 0.775 |  |
| Temperature | -0.4126 | -0.3238 | 0.2751 | 27.5061 | 0.334 |  |
| Windspeed | -0.2638 | -0.2623 | 0.1384 | 13.8350 | 0.578 |  |
| NDVI | -0.5239 | -0.6188 | 0.6575 | 65.7464 | **0.024** | * |

(**d**) **Mantel tests between Bray Curtis community dissimilarity and environmental distances (Excluding FLT7)**

| **Environmental Variable** | **Mantel r** | **p** |  |
| --- | --- | --- | --- |
| Altitude | -0.2450 | 0.972 |  |
| Temperature | -0.0233 | 0.466 |  |
| Windspeed | -0.0394 | 0.565 |  |
| NDVI | 0.3014 | 0.050 | ***** |

**Figure S8, continued**. Environmental vector fitting (envfit) and Mantel test results associated with NMDS ordinations of fungal community composition based on Bray-Curtis dissimilarity matrices for all flights and excluding FLT7. NMDS vector coordinates (NMDS1 and NMDS2), coefficients of determination (R²) and permutation-based p-values.

(**e**) **Bray-Curtis NMDS ordination analysis on OTU community composition excluding FLT7.** R^2^ values show the proportion of deviance in the ordination space explained by the focal environmental covariate.

**Figure S9.** NMDS with ANOSIM of environmental variables for all flights colored by season. Note that variables within this ordination are likely correlated. Refer to **Figure S8** for uncorrelated predictor variable ordination.

**Figure S10.** Weighted prevalence of fungi richness for all OTUs (dominant + minor) assigned to trophic guilds. A total of 121 of 166 OTUs were assigned to guilds.

**Figure S11.** Significant relationships between airborne fungi OTU abundance and environmental factors in Georgia.

Significant values of environmental variables associated with relative abundance:

| **variable** | **pvalue** |
| --- | --- |
| Sea level pressure | 0.001096 |
| Wind gust | 0.001174 |
| Temperature_daily mean | 0.001467 |
| Dewpoint | 0.001628 |
| Windspeed | 0.00183 |
| Cloud cover | 0.005357 |
| NDVI | 0.011501 |
| Humidity | 0.012987 |
| Density Altitude | 0.018081 |

**Figure S12.** Top fungal taxa significantly associated with NDVI, where negative coefficients indicate a negative or inverse relationship. *p*-values are provided.

| **Taxon** | **OTU** | **Coefficient** | **P_value** |
| --- | --- | --- | --- |
| **OTU33.NDVI_1km_monthly** | **OTU33** | **-13.1903** | **0.034677** |
| **OTU2.NDVI_1km_monthly** | **OTU2** | **-10.8728** | **0.009236** |
| OTU35.NDVI_1km_monthly | OTU35 | -3.39466 | 0.00683 |
| OTU32.NDVI_1km_monthly | OTU32 | -2.30004 | 0.042614 |
| **OTU7.NDVI_1km_monthly** | **OTU7** | **-2.14012** | **0.002099** |
| OTU130.NDVI_1km_monthly | OTU130 | -2.07702 | 0.045695 |
| OTU30.NDVI_1km_monthly | OTU30 | -1.82713 | 0.041788 |
| OTU158.NDVI_1km_monthly | OTU158 | -1.76765 | 0.03918 |
| OTU142.NDVI_1km_monthly | OTU142 | -1.66019 | 0.041613 |
| OTU153.NDVI_1km_monthly | OTU153 | -1.49576 | 0.042928 |
| OTU69.NDVI_1km_monthly | OTU69 | -1.24292 | 0.043414 |
| OTU147.NDVI_1km_monthly | OTU147 | -1.11835 | 0.041157 |
| OTU46.NDVI_1km_monthly | OTU46 | -1.03519 | 0.044916 |
| OTU79.NDVI_1km_monthly | OTU79 | -0.86337 | 0.042083 |
| OTU111.NDVI_1km_monthly | OTU111 | -0.74621 | 0.03955 |
| OTU126.NDVI_1km_monthly | OTU126 | -0.69128 | 0.044068 |
| OTU93.NDVI_1km_monthly | OTU93 | -0.61655 | 0.034305 |
| OTU121.NDVI_1km_monthly | OTU121 | -0.61048 | 0.042744 |
| OTU48.NDVI_1km_monthly | OTU48 | -0.5517 | 0.042574 |
| OTU159.NDVI_1km_monthly | OTU159 | -0.54234 | 0.03948 |

Top Taxa: **Bold** indicates the taxon is dominant. Non bolded text indicates taxa that are minor (rare or transient).

**Figure S13.** **Environmental and seasonal variation in atmospheric fungal diversity on aircraft survey flights. Regression analyses for atmospheric mycobiome richness.** **a)** Airborne fungi trophic guild richness response to environmental predictors for dominant, minor, and all taxa (OTUs) for flights excluding sample FLT7 in SC (thus, for GA only). Guilds with ≤3 nonzero flights containing total, dominant, and minor OTUs are shown. Panels show bivariate relationships between guild richness [log(guild richness + 1)] and focal environmental predictors (Altitude, Temperature, Windspeed, and normalized difference vegetation indices, NDVI). Points represent observed log-transformed guild richness values (number of OTUs assigned to each guild per sample solid lines represent ordinary least-squares linear regressions with 95% confidence intervals. Significance annotations indicate results of separate univariate regressions fitted independently for each guild–predictor combination (* p < 0.05, ** p < 0.01, *** p < 0.001). The same analysis with all flights is shown in the main article. **b)** Regression of fungal guild-assigned dominant and minor OTU richness across all flights. Lower NDVI values reflect lower photosynthetic activity. Graph Builder in SAS JMP was used to graph log-transformed Guild OTU richness for dominant and minor OTUs for b against environmental variables vegetative index NDVI and windspeed. Scripts used are in the Rcode.R and Scripts.SAS files.

**a)** Airborne fungi trophic guild richness response to environmental predictors for dominant, minor, and all taxa (OTUs) for flights excluding sample FLT7 in SC (thus, for GA only).

**b)** Regression of fungal guild-assigned dominant and minor OTU richness across all flights.

1. [All] OTU richness v. NDVI:

2. Minor OTU richness v. NDVI:

3. Dominant OTU richness v. NDVI:

4. Dominant OTU richness v. NDVI (GA samples):

5. Minor OTU richness v. NDVI (GA samples):

6. [All] OTU richness v. Wind speed:

7. Dominant OTU richness v. Wind speed:

8. Minor OTU richness v. Wind speed:

**Figure S14.** Univariate relationships between OTU richness of airborne fungi and environmental variables in Georgia. OTU richness regressed against day of year (**a**), vegetation index of aboveground biomass productivity and greenness (**b**), solar radiation (**c**), solar energy (**d**), UV index (**e**), density altitude (**f**), and cloud cover (**h**). Only significant (*p*<0.05) results or those approaching significance are displayed. Note that multicollinearity exists within some of these variables. Scripts used are in the Scripts.SAS file.

**a	b	c**

**d	e	f**

**g**

**Figure S15.** Univariate relationships between OTU richness of airborne fungi and environmental variables in the SEUS. All samples are included. OTU richness regressed against wind gust (**a**), wind speed (**b**), vegetation index of aboveground biomass productivity and greenness, NDVI (**c**), UV index (**d**), solar energy (**e**), solar radiation (**f**), and day of year (**g**). Only significant ( *p*<0.05) results or those approaching significance are displayed. Note that multicollinearity exists within some of these variables. Scripts used are in the Scripts.SAS file.

**a	b	c**

**d	e	f**

**g**

**Table S1.1a.** Abiotic environmental data for research flights conducted in this study. FLT = flight; ave = average/mean; Weather descriptors and units are mean values: Temperature (temp, °C); dewpoint (dew, °C); relative humidity (humidity, %), precipitation (precip, mm), wind gust and speed (mph), wind direction (winddir, degrees), cloud cover (%), visibility (vis, km), sea level pressure (SLP, mb), solar radiation (solar_rad, W/m2), and mean solar energy (solar_e, MJ/m2). Temperature at altitude and density altitude were also included in this dataset and can be found in **Table S1.1b**. Density altitude values were averaged if they changed during the course of the flight.

| **FLT** | **Location** | **Date** | **temp** | **dew** | **humidity** | **precip** | **windgust** | **windspeed** | **winddir** | **SLP** | **cloudcover** | **vis** | **solar_rad** | **solar_e** | **UVindex** |
| --- | --- | --- | --- | --- | --- | --- | --- | --- | --- | --- | --- | --- | --- | --- | --- |
| 0 | Hartwell, GA | 2-May-22 | 22.91667 | 16.15833 | 69.26458 | 0 | 20.725 | 9.1875 | 225.125 | 1016.979 | 10.3875 | 15.7583 | 76.875 | 0.270833 | 0.75 |
| 1 | Hartwell, GA | 9-May-22 | 16.10417 | 7.570833 | 58.61375 | 0 | 19.675 | 10.05833 | 68.08333 | 1020.708 | 43.97083 | 16 | 90.58333 | 0.316667 | 0.95833 |
| 2 | Hartwell, GA | 9-May-22 | 16.10417 | 7.570833 | 58.61375 | 0 | 19.675 | 10.05833 | 68.08333 | 1020.708 | 43.97083 | 16 | 90.58333 | 0.316667 | 0.95833 |
| 3 | Hartwell, GA | 9-May-22 | 16.10417 | 7.570833 | 58.61375 | 0 | 19.675 | 10.05833 | 68.08333 | 1020.708 | 43.97083 | 16 | 90.58333 | 0.316667 | 0.95833 |
| 4 | Hartwell, GA | 4-Jun-22 | 22.97083 | 15.4375 | 65.88333 | 0 | 21.77917 | 10.9625 | 82.16667 | 1011.996 | 30.15417 | 15.9333 | 78.375 | 0.279167 | 0.79167 |
| 5 | Hartwell, GA | 4-Jun-22 | 22.97083 | 15.4375 | 65.88333 | 0 | 21.77917 | 10.9625 | 82.16667 | 1011.996 | 30.15417 | 15.9333 | 78.375 | 0.279167 | 0.79167 |
| 6 | Hartwell, GA | 4-Jun-22 | 22.97083 | 15.4375 | 65.88333 | 0 | 21.77917 | 10.9625 | 82.16667 | 1011.996 | 30.15417 | 15.9333 | 78.375 | 0.279167 | 0.79167 |
| 7 | Clemson SC | 23-Sep-23 | 20.79583 | 13.79167 | 66.80917 | 0 | 12.23333 | 5.733333 | 237.9583 | 1012.079 | 0.141667 | 15.9916 | 251.7917 | 0.908333 | 2.5 |
| 8 | Hartwell, GA | 12-Nov-23 | 11.65417 | 3.8125 | 60.22958 | 0 | 19.125 | 8.845833 | 43.5 | 1026.908 | 57.57083 | 16 | 20.125 | 0.075 | 0.25 |
| 9 | Hartwell, GA | 12-Nov-23 | 11.65417 | 3.8125 | 60.22958 | 0 | 19.125 | 8.845833 | 43.5 | 1026.908 | 57.57083 | 16 | 20.125 | 0.075 | 0.25 |
| 10 | Hartwell, GA | 12-Nov-23 | 11.65417 | 3.8125 | 60.22958 | 0 | 19.125 | 8.845833 | 43.5 | 1026.908 | 57.57083 | 16 | 20.125 | 0.075 | 0.25 |

**Table S1.1b.** Aviation meteorological data for research flights conducted in this study. SM: statute miles, MSL: mean sea level, AGL: above ground level, Ave: average, NM: nautical miles, WTA: winds and temperatures aloft; VAR: variable (wind direction varies by 180° or more); G: gusting. The wind direction given indicates that the wind comes in front of the aircraft when its heading equals the wind direction. When the wind is gusty with variations from the mean wind speed (“G”, gusting) exceeding 5 m·s^-1^ or 10 kts, speed variations are indicated after the mean wind direction and speed. OVC=overcast ceiling.

| **Flight #** | **Date** | **Sampling time**  **local** (hhmm) | **METAR Zulu** (UTC) | **Wind Direction** (°) | **Wind Velocity** (kts) | **Visibility** (SM) | **Sky Cdn/Ceiling** | **Air Temp** (°C) | **Dewpoint** (°C) | **Altimeter** (in-Hg) | **Density Altitude** (ft) | **Temp at Altitude** (°C) |
| --- | --- | --- | --- | --- | --- | --- | --- | --- | --- | --- | --- | --- |
| 0 | 2-May-22 | 1901-2016 | 2254 | 240 | 08 | 10 | CLR | 29 | 16 | 3000 | 2500 | 13 |
| 1 | 9-May-22 | 1837-1953 | 2225 | 080 | 05 | 10 | CLR | 23 | 08 | 3012 | 1700 | 14 |
| 2 | 9-May-22 | 1638-1754 | 2028 | 100 | 03 | 10 | CLR | 22 | 08 | 3013 | 1600 | 17 |
| 3 | 9-May-22 | 1429-1546 | 1811 | VAR | 03 | 10 | CLR | 20 | 07 | 3017 | 1300 | 11 |
| 4 | 4-Jun-22 | 1311-1426 | 1659 | 080 | 11G19 | 10 | CLR | 27 | 14 | 2992 | 2400 | 20 |
| 5 | 4-Jun-22 | 0944-1101 | 1328 | 040 | 10 | 10 | CLR | 23 | 14 | 2993 | 1900 | 15 |
| 6 | 4-Jun-22 | 0735-0850 | 1111 | 030 | 05 | 10 | CLR | 19 | 16 | 2992 | 1500 | 10 |
| 7 | 23-Sep-23 | 1327-1457 | 1708Z | 350 | 07 | 10 | CLR | 28 | 12 | 2989 | 2400 | 20 |
| 8 | 12-Nov-23 | 1323-1433 | 1810Z | 090 | 07 | 10 | OVC50 | 16 | 02 | 3033 | 600 | 11 |
|  |  |  | 1948Z | 120 | 06 | 10 | CLR | 16 | 02 | 3030 | 700 | 11 |
| 9 | 12-Nov-23 | 1524-1624 | 2011Z | 070 | 09 | 10 | CLR | 16 | 02 | 3029 | 700 | 10 |
|  |  |  | 2124Z | 070 | 03 | 10 | CLR | 16 | 02 | 3030 | 600 | 10 |
| 10 | 12-Nov-23 | 1709-1750 | 2149Z | 080 | 03 | 10 | CLR | 15 | 02 | 3030 | 600 | 08 |
|  |  |  | 2250Z | calm | 03 | 10 | CLR | 12 | 04 | 3031 | 200 | 06 |

| **Flight #** | **Date** | **Altitude** (m AGL) | **Altitude** (ft MSL) | **Sampling duration**  (min) | **Total distance** (NM) |
| --- | --- | --- | --- | --- | --- |
| 0 | 2-May-22 | 300 | 1800 | 75 | 107 |
| 1 | 9-May-22 | 300 | 1800 | 75 | 108 |
| 2 | 9-May-22 | 1200 | 4500 | 75 | 104 |
| 3 | 9-May-22 | 2500 | 8500 | 75 | 100 |
| 4 | 4-Jun-22 | 300 | 1800 | 75 | 111 |
| 5 | 4-Jun-22 | 1200 | 4500 | 75 | 106 |
| 6 | 4-Jun-22 | 2500 | 8500 | 75 | 105 |
| 7 | 23-Sep-23 | 500 | 2500 | 90 | 126 |
| 8 | 12-Nov-23 | 300 | 1800 | 70 | 106 |
| 9 | 12-Nov-23 | 1200 | 4500 | 60 | 95 |
| 10 | 12-Nov-23 | 2500 | 8500 | 41 | 62 |

| **Flight #** | **Date** | **Altitude** (m AGL) | **Flow, D1** (L·min^-1^) | **Flow, D2** (L·min^-1^) | **Pressure, D1** (-kPa) | **Pressure, D2** (-kPa) | **Total Air Volume^‡^** (L) | **Total Air Volume^‡^** (m^3^) |
| --- | --- | --- | --- | --- | --- | --- | --- | --- |
| FLT0^†^ | 2-May-22 | 300 | 15 | 14 | 52 | 48 | 2,175 | 2.175 |
| FLT1 | 9-May-22 | 300 | 10 | 10 | 42 | 42 | 1,500 | 1.500 |
| FLT2 | 9-May-22 | 1200 | 10 | 13 | 51 | 55 | 1,725 | 1.725 |
| FLT3 | 9-May-22 | 2500 | 11 | 7 | 55 | 48 | 1,350 | 1.350 |
| FLT4 | 4-Jun-22 | 300 | 15 | 14 | 40 | 46 | 2,175 | 2.175 |
| FLT5 | 4-Jun-22 | 1200 | 14 | 14 | 50 | 50 | 2,100 | 2.100 |
| FLT6 | 4-Jun-22 | 2500 | 14 | 12 | 58 | 56 | 1,950 | 1.950 |
| FLT7 | 23-Sep-23 | 500 | 24 | 25 | 50 | 55 | 4,410 | 4.410 |
| FLT8 | 12-Nov-23 | 300 | 20 | 23 | 65 | 60 | 3,010 | 3.010 |
| FLT9 | 12-Nov-23 | 1200 | 20 | 21 | 55 | 60 | 2,460 | 2.460 |
| FLT10 | 12-Nov-23 | 2500 | 19 | 20 | 48 | 50 | 1,558 | 1.599 |

**Table S1.2.** Sampler performance data for research flights conducted in the SEUS. MSL: mean sea level, AGL: above ground level, D1/D2: duplicate 1/duplicate 2 average. ^†^FLT0 is equivalent to Test_2 in *Metris and Metris 2023* and FLT1-FLT6 are equivalent to FLT1-FLT6 in the same publication. **^‡^**Total air volume filtered was calculated as the product of airflow × sampling time.

**Table S1.3.** Fungi sequence reads recovered in this study. Proportions of reads and percentages for core phyla Ascomycota (AMC) and Basidiomycota (BMC). †Non AMC and BMC “other reads” include Mucoromycota, Mortierellomycota, and undefined phyla. ‡These three altitudes were flown on the same day and location thus data for 9-May-22, 4-Jun-22, and 12-Nov-23 flights are comparable and combined by altitude. # = number. Data for FLT0 (300 m) (same location, but one altitude, 300 m, on 2-May-22) and FLT7 (500 m) (23-Sep-23) are provided separately (grey shading).

| **Altitude (AGL)** | **#reads AMC (%)** | **#reads BMC (%)** | **#other^†^ reads (%)** | **Total reads** |
| --- | --- | --- | --- | --- |
| 300 m^‡^ | 105,523 | 9,069 | 1,997 | 116,589 |
|  | (90.51) | (7.78) | (1.71) |  |
| 1200 m^‡^ |  |  |  |  |
|  | 57,181  (85.03) | 9,974  (14.83) | 96  (<1) | 67,251 |
|  |  |  |  |  |
| 2500 m^‡^ | 168,942  (93.80) | 9,867  (5.48) | 1,300  (<1) | 180,109 |
|  |  |  |  |  |
| FLT0 (300 m) | 27,466 | 12,341 | 175 | 39,982 |
|  | (68.69) | (30.87) | (<1) |  |
|  |  |  |  |  |
| FLT7 (500 m) | 22,217 | 4,596 | 360 | 27,173 |
|  | (81.76) | (16.91) | (<1) |  |
| All | 381,329 | 45,847 | 3,928 | 431,104 |
|  | (88.45) | (10.63) | (<1) |  |

**Table S1.4.** Negative control reads removed from the samples.

| NegCtrl for 2022 Sequencing run | NegCtrl for 2023 Sequencing run | Description |
| --- | --- | --- |
| 0 | 1 | Ascomycota |
| 1 | 0 | Botryosphaeria |
| 7 | 2 | Cladosporium cladosporioides |
| 1 | 8 | Cladosporium |
| 1 | 0 | Toxicocladosporium |
| 0 | 4 | Elsinoaceae |
| 0 | 5 | Coniothyrium |
| 15 | 13 | Didymellaceae |
| 5 | 0 | Neoascochyta desmazieri |
| 6 | 2 | Alternaria alternata |
| 0 | 1 | Penicillium |
| 0 | 2 | Penicillium coffeae |
| 0 | 1 | Penicillium |
| 1 | 0 | Golovinomyces |
| 2 | 0 | Lachnum |
| 3 | 0 | Helotiales |
| 1 | 0 | Fusarium |
| 1 | 0 | Fusarium oxysporum |
| 2 | 0 | Hypocreales |
| 2 | 0 | Trichocladium opacum |
| 1 | 0 | Xylariales |
| 0 | 1 | Ascomycota |
| 73 | 66 | Agaricus bisporus |
| 1 | 0 | Hymenochaetopsis |
| 0 | 1 | Sidera |
| 3 | 0 | Schizopora flavipora |
| 2 | 0 | Polyporales |
| 2 | 0 | Trametes cubensis |
| 1 | 0 | Gloeoporus dichrous |
| 1 | 0 | Polyporales |
| 0 | 8 | Agaricomycetes |
| 2 | 0 | Malasseziales |
| 1 | 0 | Fungi |

**Table S1.5.** Top 5 genera enriched at each altitude in this study.

|  | **2-May-22** | | **9-May-22** | | **4-Jun-22** | | **23-Sep-23** | | **12-Nov-23** | |
| --- | --- | --- | --- | --- | --- | --- | --- | --- | --- | --- |
| **Altitude (m)** | **Genus** | **Relative abundance (%)** | **Genus** | **Relative abundance (%)** | **Genus** | **Relative abundance (%)** | **Genus** | **Relative abundance (%)** | **Genus** | **Relative abundance (%)** |
| 300 | *Neoascochyta* | 55.49 | *Stereum* | 8.09 | *Cladosporium* | 35.66 |  |  | *Cladosporium* | 66.43 |
| 300 | *Hymenochaetopsis* | 17.05 | *Cladosporium* | 2.17 | *Neoascochyta* | 26.76 |  |  | *Penicillium* | 6.08 |
| 300 | *Fonsecazyma* | 13.52 | *Neoascochyta* | 1.86 | *Ramularia* | 4.34 |  |  | *Hannaella* | 2.35 |
| 300 | *Ramularia* | 5.85 | *Hymenochaetopsis* | 0.85 | *Colletogloeum* | 4.26 |  |  | *Neoascochyta* | 2.27 |
| 300 | *Mortierella* | 0.43 | *Russula* | 0.21 | *Alternaria* | 3.67 |  |  | *Cercospora* | 2.17 |
| 500 |  |  |  |  |  |  | *Cladosporium* | 38.66 |  |  |
| 500 |  |  |  |  |  |  | *Alternaria* | 9.52 |  |  |
| 500 |  |  |  |  |  |  | *Neoascochyta* | 4.63 |  |  |
| 500 |  |  |  |  |  |  | *Periconia* | 2.23 |  |  |
| 500 |  |  |  |  |  |  | *Curvularia* | 2.11 |  |  |
| 1200 |  |  | *Neoascochyta* | 86.61 | *Neoascochyta* | 43.54 |  |  | *Cladosporium* | 33.85 |
| 1200 |  |  | *Aspergillus* | 7.22 | *Lachnum* | 16.82 |  |  | *Coniothyrium* | 27.34 |
| 1200 |  |  | *Stereum* | 0.41 | *Cladosporium* | 7.42 |  |  | *Penicillium* | 11.43 |
| 1200 |  |  | *Fusarium* | 0.21 | *Phlebiopsis* | 4.14 |  |  | *Neoascochyta* | 3.20 |
| 1200 |  |  | *Cladosporium* | 0.19 | *Schizopora* | 3.64 |  |  | *Alternaria* | 3.08 |
| 2500 |  |  | *Neoascochyta* | 69.41 | *Cladosporium* | 11.43 |  |  | *Cladosporium* | 26.18 |
| 2500 |  |  | *Cladosporium* | 17.47 | *Toxicocladosporium* | 5.28 |  |  | *Neoascochyta* | 11.06 |
| 2500 |  |  | *Taphrina* | 0.67 | *Myrmecridium* | 4.74 |  |  | *Mycosphaerella* | 4.86 |
| 2500 |  |  | *Malassezia* | 0.35 | *Alternaria* | 3.36 |  |  | *Periconia* | 3.16 |
| 2500 |  |  | *Alutaceodontia* | 0.16 | *Golovinomyces* | 3.19 |  |  | *Coniothyrium* | 2.35 |

**Table S1.6. Taxon Richness All Flights**

(**a**) **Taxon richness overall model comparisons for all flights.**

Summary statistics for null, primary, and seasonal multiple regression models predicting taxon richness across all flights. Reported values include model R², adjusted R² (Adj. R²), Akaike Information Criterion (AIC), residual standard error (ResSE), F-statistic, p-value from ordinary least-squares linear models, and significance classification. Primary models included altitude, temperature, and windspeed predictors; seasonal models additionally included NDVI. The best statistically supported model is shown in **bold**. Significance notations: † p < 0.10; * p < 0.05; ** p < 0.01; *** p < 0.001; ns = not significant.

| **Model** | **Predictors** | **R^2^** | **Adj. R^2^** | **AIC** | **ResSE** | **F** | **p** | **Sig.** |
| --- | --- | --- | --- | --- | --- | --- | --- | --- |
| Null | Intercept Only | 0 | 0 | 16.88181 | 0.455779 |  |  |  |
| Primary | Altitude + Temperature + Windspeed | 0.539977 | 0.342824 | 14.34055 | 0.369484 | 2.738874 | 0.122957 | ns |
| **Seasonal** | Altitude + Temperature + Windspeed  + NDVI | 0.743426 | 0.572377 | 9.918076 | 0.298047 | 4.346273 | *0.05456* | † |

(**b**) **ANOVA comparisons for models of taxon richness for all flights.**
Analysis of variance (ANOVA) comparisons among null, primary, and seasonal multiple regression models predicting taxon richness across all flights. Comparisons include Null vs Primary, Primary vs Seasonal, and Null vs Seasonal models. Reported values include F-statistics, p-values, and effect sizes (η²). Larger η² values indicate greater explanatory improvement associated with the more complex model.

| **Comparison** | **F-stat** | **p** | **η²** |
| --- | --- | --- | --- |
| Null vs Primary | F(3, 7) = 2.7389 | 0.122957 | 0.539977 |
| Primary vs Seasonal | F(1, 6) = 4.7577 | 0.071941 | 0.442259 |
| Null vs Seasonal | F(4, 6) = 4.3463 | 0.05456 | 0.743426 |

(**c**) **Predictor coefficient estimates for seasonal taxon richness model for all flights.**

Estimated regression coefficients (β), standard errors (SE), t-statistics, and p-values for all fixed effect predictors included in the seasonal multiple regression models of taxon richness across all flights. Primary models included altitude, temperature, and windspeed, whereas seasonal models additionally incorporated NDVI as a proxy for seasonal vegetation dynamics. Positive coefficient values indicate positive associations between environmental predictors and taxon richness, whereas negative values indicate inverse relationships. Statistical significance of individual predictors is indicated by the following notation: † p < 0.10; * p < 0.05; ** p < 0.01; *** p < 0.001; ns = not significant. Only analyses whose overall models were statistically significant or marginally significant and were retained in the final analysis are shown.

| **Predictor** | **Estimate (β)** | **SE** | **t** | **p** | **Sig.** |
| --- | --- | --- | --- | --- | --- |
| NDVI | -3.06942 | 1.407211 | -2.18121 | 0.071941 | † |
| Altitude | 2.039330e-05 | 0.000108 | 0.188637 | 0.856596 | ns |
| Temp | 0.030413 | 0.022903 | 1.327903 | 0.232491 | ns |
| Windspeed | -0.01053 | 0.12259 | -0.08592 | 0.934323 | ns |

(**d**) **Fixed-effect comparison between least squares regression and mixed-model sensitivity analysis for taxon richness including date as a random effect for all flights.** No results from primary-mixed model date sensitivity analysis approached statistical significance. Thus, no conclusions in terms of significance changed from least squares regression primary models to mixed-model date sensitivity comparison.

| **Model** | **Random effect** | **Variance** | **SD** |
| --- | --- | --- | --- |
| Primary mixed | Date | 0.001313099 | 0.0362367 |
| Seasonal mixed | Date | 0.000000000 | 0.0000000 |

| **Model** | **Predictor** | **LS p** | **Mixed p** | **OLS Sig.** | **Mixed Sig.** |
| --- | --- | --- | --- | --- | --- |
| **Seasonal** | **NDVI** | 0.071941 | 0.013130 | † | * |
| Seasonal | Altitude | 0.856596 | 0.803115 | ns | ns |
| Seasonal | Temperature | 0.232491 | 0.099650 | ns | † |
| Seasonal | Windspeed | 0.934323 | 0.909479 | ns | ns |

(**e**) **All flights taxon richness model assumption diagnostics.**

| **Model** | **Predictor** | **Variance Inflation Factor (VIF)** |
| --- | --- | --- |
| Primary | Altitude | 1.1336 |
| Primary | Temperature | 1.1420 |
| Primary | Windspeed | 1.1792 |
| Seasonal | NDVI | 4.3178 |
| Seasonal | Altitude | 1.1559 |
| Seasonal | Temperature | 1.4013 |
| Seasonal | Windspeed | 3.8777 |

| **Model** | **Temporal Autocorrelation Test** | **Statistic** | **p-value** |
| --- | --- | --- | --- |
| Primary | Ljung–Box | 2.8698 | 0.0903 |
| Seasonal | Ljung–Box | 0.5783 | 0.4470 |

**Table S1.7. Taxon Richness Georgia Flights Only (Excluding FLT7)**

(**a**) **Taxon richness overall model comparisons excluding FLT7.**

Summary statistics for null, primary, and seasonal least squares regression models predicting taxon richness after exclusion of sample FLT7. Reported values include model R², adjusted R² (Adj. R²), Akaike Information Criterion (AIC), residual standard error (ResSE), F-statistic, model p-value, and significance classification. Primary models included altitude, temperature, and windspeed predictors; seasonal models additionally included NDVI. Neither model was statistically significant. Significance notations: † p < 0.10; * p < 0.05; ** p < 0.01; *** p < 0.001; ns = not significant.

| **Model** | **R^2^** | **Adj. R^2^** | **AIC** | **ResSE** | **F** | **p** | **Sig** |
| --- | --- | --- | --- | --- | --- | --- | --- |
| Null | 0 | 0 | 8.325404 | 0.316641 |  |  |  |
| Primary | 0.192171 | -0.21174 | 12.19136 | 0.348555 | 0.475771 | 0.710598 | ns |
| Seasonal | 0.562373 | 0.212272 | 8.061513 | 0.281031 | 1.606316 | 0.30482 | ns |

(**b**) **ANOVA comparisons for models of taxon richness excluding FLT7.**
Analysis of variance (ANOVA) comparisons among null, primary, and seasonal trophic guild richness models after excluding sample FLT7. Comparisons include Null vs Primary, Primary vs Seasonal, and Null vs Seasonal models. Reported values include F-statistics, p-values, and effect sizes (η²). Larger η² values indicate greater explanatory improvement associated with the more complex model. No model comparisons were statistically significant.

| **Comparison** | **F-stat** | **p** | **η²** |
| --- | --- | --- | --- |
| Null vs Primary | F(3, 6) = 0.4758 | 0.710598 | 0.192171 |
| Primary vs Seasonal | F(1, 5) = 4.2297 | 0.094841 | 0.458268 |
| Null vs Seasonal | F(4, 5) = 1.6063 | 0.30482 | 0.562373 |

(**c**) **Predictor coefficient estimates for seasonal taxon richness model excluding FLT7.**

Estimated regression coefficients (β), standard errors (SE), t-statistics, and p-values for all fixed effect predictors included in the seasonal multiple regression models of taxon richness after excluding FLT7. Primary models included altitude, temperature, and windspeed, whereas seasonal models additionally incorporated NDVI as a proxy for seasonal vegetation dynamics. Positive coefficient values indicate positive associations between environmental predictors and taxon richness, whereas negative values indicate inverse relationships. Statistical significance of individual predictors is indicated by the following notation: † p < 0.10; * p < 0.05; ** p < 0.01; *** p < 0.001; ns = not significant. Only analyses whose overall models were statistically significant or marginally significant and were retained in the final analysis are shown.

| **Predictor** | **Estimate (β)** | **SE** | **t** | **p** | **Sig.** |
| --- | --- | --- | --- | --- | --- |
| NDVI | -2.769 | 1.346 | -2.057 | 0.0948 | † |
| Altitude | -0.000005 | 0.000104 | -0.048 | 0.9634 | ns |
| Temp | -0.00368 | 0.03363 | -0.110 | 0.9170 | ns |
| Windspeed | 0.1809 | 0.1852 | 0.976 | 0.3737 | ns |

(**d**) **Comparison of fixed-effect significance between least squares and mixed-effects seasonal taxon richness models excluding FLT7. (a)** Mixed-effects models were included as sensitivity analyses to assess the potential influence of non-independence among samples collected on the same date and variance collapsed to zero in this mixed-model. **(b)** Estimated p-values for each predictor in the seasonal multiple linear regression model and the corresponding mixed-effects model including sampling date as a random intercept to evaluate whether predictor significance changed following inclusion of the random effect. In the seasonal analysis excluding FLT7, NDVI was marginally significant in the least squares regression model (p = 0.095) but significant in the mixed-effects model (p = 0.016). Statistical significance: † p < 0.10; * p < 0.05; ** p < 0.01; *** p < 0.001; ns = not significant. No results from primary-mixed model date sensitivity analysis approached statistical significance. Thus, no conclusions in terms of significance changed from least squares regression primary models to mixed-model date sensitivity comparisons.

**Mixed-Effects sensitivity analysis comparisons (excluding FLT7).**

| **Model** | **Random effect** | **Variance** | **SD** |
| --- | --- | --- | --- |
| Primary mixed | Date | 0.0113074 | 0.1063363 |
| Seasonal mixed | Date | 0.0000000 | 0.0000000 |

| **Model** | **Predictor** | **LS p** | **Mixed p** | **LS Sig.** | **Mixed Sig.** |
| --- | --- | --- | --- | --- | --- |
| Seasonal | NDVI | 0.094841 | *0.015605* | † | * |
| Seasonal | Altitude | 0.963387 | 0.946946 | ns | ns |
| Seasonal | Temperature | 0.917038 | 0.879977 | ns | ns |
| Seasonal | Windspeed | 0.373716 | 0.197413 | ns | ns |

LS = Least-squares; Mixed = Mixed-model.

(**e**) **Taxon richness model assumption diagnostics (excluding FLT7).**

| **Model** | **Predictor** | **Variance Inflation Factor (VIF)** |
| --- | --- | --- |
| Primary | Altitude | 1.118860 |
| Primary | Temperature | 2.732585 |
| Primary | Windspeed | 2.710250 |
| Seasonal | NDVI | 2.763978 |
| Seasonal | Altitude | 1.130926 |
| Seasonal | Temperature | 3.258609 |
| Seasonal | Windspeed | 3.170717 |

| **Model** | **Temporal Autocorrelation Test** | **Statistic** | **p-value** |
| --- | --- | --- | --- |
| Primary | Ljung–Box | 2.984430 | 0.084069 |
| Seasonal | Ljung–Box | 0.009576 | 0.922047 |

**Table S1.8. Guild Richness All Flights**

(**a**) **Guild richness overall model performance comparisons for all flights.**

Summary statistics for null, primary, and seasonal multiple regression models predicting trophic guild richness across all flights. Reported values include model R², adjusted R² (Adj. R²), Akaike Information Criterion (AIC), residual standard error (ResSE), F-statistic, p-value from ordinary least-squares linear models, and significance classification. Primary models included altitude, temperature, and windspeed predictors; seasonal models additionally included NDVI. The best statistically supported significant models are in **bold**. Significance notations: † p < 0.10; * p < 0.05; ** p < 0.01; *** p < 0.001; ns = not significant.

| **All Flights**  **Analysis** | **Guild** | **Model** | **R²** | **Adj. R²** | **AIC** | **ResSE** | **F** | **p** | **Sig.** |
| --- | --- | --- | --- | --- | --- | --- | --- | --- | --- |
| Dominant | Plant Pathogen | Null | 0 | 0 | -5.43445 | 0.165279 |  |  | — |
| Dominant | Plant Pathogen | Primary | 0.204199 | -0.13686 | -1.94692 | 0.176226 | 0.598723 | 0.63592 | ns |
| Dominant | Plant Pathogen | Seasonal | 0.207466 | -0.32089 | 0.007831 | 0.189955 | 0.392663 | 0.807628 | ns |
| Dominant | Undefined Saprotroph | Null | 0 | 0 | 6.912587 | 0.289705 |  |  | — |
| Dominant | Undefined Saprotroph | Primary | 0.25443 | -0.0651 | 9.682915 | 0.298986 | 0.796264 | 0.533851 | ns |
| Dominant | Undefined Saprotroph | Seasonal | 0.380871 | -0.03188 | 9.638732 | 0.294287 | 0.922758 | 0.508496 | ns |
| Dominant | Animal Pathogen | Null | 0 | 0 | -9.98824 | 0.134376 |  |  | — |
| Dominant | Animal Pathogen | Primary | 0.198548 | -0.14493 | -6.42288 | 0.143784 | 0.57805 | 0.647698 | ns |
| Dominant | Animal Pathogen | **Seasonal** | 0.764362 | 0.60727 | -17.8883 | 0.084211 | 4.865702 | 0.043086 | * |
| Dominant | Wood Saprotroph | Null | 0 | 0 | 7.5927 | 0.298801 |  |  | — |
| Dominant | Wood Saprotroph | Primary | 0.264287 | -0.05102 | 10.21664 | 0.306328 | 0.838192 | 0.514541 | ns |
| Dominant | Wood Saprotroph | Seasonal | 0.403807 | 0.006346 | 9.903592 | 0.297851 | 1.015966 | 0.468631 | ns |
| Dominant | Endophyte | Null | 0 | 0 | 17.61496 | 0.471224 |  |  | — |
| Dominant | Endophyte | Primary | 0.171777 | -0.18318 | 21.54176 | 0.512569 | 0.483943 | 0.703929 | ns |
| Dominant | Endophyte | Seasonal | 0.742456 | 0.57076 | 10.69275 | 0.308729 | 4.324249 | 0.055132 | † |
| Dominant | Dung Saprotroph | Null | 0 | 0 | 11.05392 | 0.34971 |  |  | — |
| Dominant | Dung Saprotroph | Primary | 0.769726 | 0.671037 | 0.90058 | 0.200577 | 7.799521 | 0.012356 | * |
| Dominant | Dung Saprotroph | **Seasonal** | 0.987848 | 0.979747 | -29.4592 | 0.049768 | 121.9394 | 7.11208E-06 | *** |
| Minor | Plant Pathogen | Null | 0 | 0 | 30.56568 | 0.848952 |  |  | — |
| Minor | Plant Pathogen | Primary | 0.658709 | 0.512442 | 24.74045 | 0.592784 | 4.503461 | 0.046303 | * |
| Minor | Plant Pathogen | **Seasonal** | 0.790019 | 0.650032 | 21.39755 | 0.502224 | 5.643509 | 0.031202 | * |
| Minor | Undefined Saprotroph | Null | 0 | 0 | 29.1414 | 0.795732 |  |  | — |
| Minor | Undefined Saprotroph | Primary | 0.698038 | 0.568625 | 21.96942 | 0.52263 | 5.393899 | 0.030794 | * |
| Minor | Undefined Saprotroph | **Seasonal** | 0.853587 | 0.755978 | 16.00683 | 0.39308 | 8.744995 | 0.011176 | * |
| Minor | Animal Pathogen | Null | 0 | 0 | 21.69375 | 0.567212 |  |  | — |
| Minor | Animal Pathogen | Primary | 0.396441 | 0.137773 | 22.13972 | 0.526691 | 1.532625 | 0.288295 | ns |
| Minor | Animal Pathogen | Seasonal | 0.466039 | 0.110065 | 22.79199 | 0.535087 | 1.309194 | 0.365089 | ns |
| Minor | Wood Saprotroph | Null | 0 | 0 | 30.12641 | 0.832169 |  |  | — |
| Minor | Wood Saprotroph | Primary | 0.594289 | 0.420413 | 26.20316 | 0.633536 | 3.417886 | 0.081823 | † |
| Minor | Wood Saprotroph | **Seasonal** | 0.844897 | 0.741495 | 17.62611 | 0.423103 | 8.170976 | 0.013189 | * |
| Minor | Endophyte | Null | 0 | 0 | 23.63429 | 0.619517 |  |  | — |
| Minor | Endophyte | Primary | 0.763817 | 0.662596 | 13.75966 | 0.359856 | 7.546007 | 0.013464 | * |
| Minor | Endophyte | **Seasonal** | 0.808501 | 0.680834 | 13.45271 | 0.349994 | 6.332925 | 0.024056 | * |
| Minor | Dung Saprotroph | Null | 0 | 0 | 18.47924 | 0.490105 |  |  | — |
| Minor | Dung Saprotroph | Primary | 0.460332 | 0.229046 | 17.69443 | 0.430332 | 1.990314 | 0.204117 | ns |
| Minor | Dung Saprotroph | Seasonal | 0.545255 | 0.242091 | 17.81105 | 0.426675 | 1.79855 | 0.247863 | ns |
| All | Plant Pathogen | Null | 0 | 0 | 14.98862 | 0.418198 |  |  | — |
| All | Plant Pathogen | Primary | 0.522489 | 0.317842 | 12.85776 | 0.345402 | 2.55312 | 0.138584 | ns |
| All | Plant Pathogen | Seasonal | 0.650901 | 0.418168 | 11.41223 | 0.318993 | 2.796774 | 0.125622 | ns |
| All | Undefined Saprotroph | Null | 0 | 0 | 20.13846 | 0.528498 |  |  | — |
| All | Undefined Saprotroph | Primary | 0.505331 | 0.29333 | 18.39593 | 0.444274 | 2.383626 | 0.155097 | ns |
| All | Undefined Saprotroph | **Seasonal** | 0.755301 | 0.592169 | 12.65345 | 0.337507 | 4.629991 | 0.047852 | * |
| All | Animal Pathogen | Null | 0 | 0 | 11.74382 | 0.36085 |  |  | — |
| All | Animal Pathogen | Primary | 0.375051 | 0.107216 | 12.57288 | 0.340958 | 1.400305 | 0.320306 | ns |
| All | Animal Pathogen | Seasonal | 0.518167 | 0.196946 | 11.71207 | 0.32337 | 1.613115 | 0.285756 | ns |
| All | Wood Saprotroph | Null | 0 | 0 | 26.39684 | 0.702405 |  |  | — |
| All | Wood Saprotroph | Primary | 0.567012 | 0.381445 | 23.18935 | 0.552429 | 3.055573 | 0.101117 | ns |
| All | Wood Saprotroph | **Seasonal** | 0.800041 | 0.666735 | 16.69075 | 0.405492 | 6.001547 | 0.027184 | * |
| All | Endophyte | Null | 0 | 0 | 24.13993 | 0.63392 |  |  | — |
| All | Endophyte | Primary | 0.578058 | 0.397226 | 20.64816 | 0.492166 | 3.196657 | 0.09298 | † |
| All | Endophyte | **Seasonal** | 0.759074 | 0.598457 | 16.484 | 0.401699 | 4.725985 | 0.045831 | * |
| All | Dung Saprotroph | Null | 0 | 0 | 18.09172 | 0.481547 |  |  | — |
| All | Dung Saprotroph | Primary | 0.437806 | 0.196866 | 17.75672 | 0.431552 | 1.817074 | 0.231831 | ns |
| All | Dung Saprotroph | Seasonal | 0.667027 | 0.445045 | 13.99509 | 0.35873 | 3.004869 | 0.110791 | ns |

(**b**) **ANOVA comparisons for models of guild richness for all flights.**
Analysis of variance (ANOVA) comparisons among null, primary, and seasonal trophic guild richness models across all flights. Comparisons include Null vs Primary, Primary vs Seasonal, and Null vs Seasonal models. Reported values include F-statistics, p-values, and effect sizes (η²). Larger η² values indicate greater explanatory improvement associated with the more complex model.

| **All Flights**  **Analysis** | **Guild** | **Comparison** | **F-Stat** | **p** | **η²** |
| --- | --- | --- | --- | --- | --- |
| Dominant | Plant Pathogen | Null vs Primary | F(3,7) = 0.5987 | 0.6359 | 0.2042 |
| Dominant | Plant Pathogen | Primary vs Seasonal | F(1,6) = 0.0247 | 0.8802 | 0.0041 |
| Dominant | Plant Pathogen | Null vs Seasonal | F(4,6) = 0.3927 | 0.8076 | 0.2075 |
| Dominant | Undefined Saprotroph | Null vs Primary | F(3,7) = 0.7963 | 0.5339 | 0.2544 |
| Dominant | Undefined Saprotroph | Primary vs Seasonal | F(1,6) = 1.2253 | 0.3107 | 0.1696 |
| Dominant | Undefined Saprotroph | Null vs Seasonal | F(4,6) = 0.9228 | 0.5085 | 0.3809 |
| Dominant | Animal Pathogen | Null vs Primary | F(3,7) = 0.5781 | 0.6477 | 0.1985 |
| Dominant | Animal Pathogen | Primary vs Seasonal | F(1,6) = 14.4072 | 0.009013 | 0.7060 |
| Dominant | Animal Pathogen | Null vs Seasonal | F(4,6) = 4.8657 | 0.04309 | 0.7644 |
| Dominant | Wood Saprotroph | Null vs Primary | F(3,7) = 0.8382 | 0.5145 | 0.2643 |
| Dominant | Wood Saprotroph | Primary vs Seasonal | F(1,6) = 1.4041 | 0.2808 | 0.1896 |
| Dominant | Wood Saprotroph | Null vs Seasonal | F(4,6) = 1.0160 | 0.4686 | 0.4038 |
| Dominant | Endophyte | Null vs Primary | F(3,7) = 0.4839 | 0.7039 | 0.1718 |
| Dominant | Endophyte | Primary vs Seasonal | F(1,6) = 13.2951 | 0.01075 | 0.6890 |
| Dominant | Endophyte | Null vs Seasonal | F(4,6) = 4.3242 | 0.05513 | 0.7425 |
| Dominant | Dung Saprotroph | Null vs Primary | F(3,7) = 7.7995 | 0.01236 | 0.7697 |
| Dominant | Dung Saprotroph | Primary vs Seasonal | F(1,6) = 107.6995 | 4.686e-05 | 0.9472 |
| Dominant | Dung Saprotroph | Null vs Seasonal | F(4,6) = 121.9394 | 7.112e-06 | 0.9878 |
| Minor | Plant Pathogen | Null vs Primary | F(3,7) = 4.5035 | 0.0463 | 0.6587 |
| Minor | Plant Pathogen | Primary vs Seasonal | F(1,6) = 3.7520 | 0.1009 | 0.3847 |
| Minor | Plant Pathogen | Null vs Seasonal | F(4,6) = 5.6435 | 0.0312 | 0.7900 |
| Minor | Undefined Saprotroph | Null vs Primary | F(3,7) = 5.3939 | 0.03079 | 0.6980 |
| Minor | Undefined Saprotroph | Primary vs Seasonal | F(1,6) = 6.3744 | 0.0450 | 0.5151 |
| Minor | Undefined Saprotroph | Null vs Seasonal | F(4,6) = 8.7450 | 0.01118 | 0.8536 |
| Minor | Animal Pathogen | Null vs Primary | F(3,7) = 1.5326 | 0.2883 | 0.3964 |
| Minor | Animal Pathogen | Primary vs Seasonal | F(1,6) = 0.7821 | 0.4105 | 0.1153 |
| Minor | Animal Pathogen | Null vs Seasonal | F(4,6) = 1.3092 | 0.3651 | 0.4660 |
| Minor | Wood Saprotroph | Null vs Primary | F(3,7) = 3.4179 | 0.08182 | 0.5943 |
| Minor | Wood Saprotroph | Primary vs Seasonal | F(1,6) = 9.6945 | 0.02075 | 0.6177 |
| Minor | Wood Saprotroph | Null vs Seasonal | F(4,6) = 8.1710 | 0.01319 | 0.8449 |
| Minor | Endophyte | Null vs Primary | F(3,7) = 7.5460 | 0.01346 | 0.7638 |
| Minor | Endophyte | Primary vs Seasonal | F(1,6) = 1.4000 | 0.2815 | 0.1892 |
| Minor | Endophyte | Null vs Seasonal | F(4,6) = 6.3329 | 0.02406 | 0.8085 |
| Minor | Dung Saprotroph | Null vs Primary | F(3,7) = 1.9903 | 0.2041 | 0.4603 |
| Minor | Dung Saprotroph | Primary vs Seasonal | F(1,6) = 1.1205 | 0.3306 | 0.1574 |
| Minor | Dung Saprotroph | Null vs Seasonal | F(4,6) = 1.7986 | 0.2479 | 0.5453 |
| All | Plant Pathogen | Null vs Primary | F(3,7) = 2.5531 | 0.1386 | 0.5225 |
| All | Plant Pathogen | Primary vs Seasonal | F(1,6) = 2.2070 | 0.1879 | 0.2689 |
| All | Plant Pathogen | Null vs Seasonal | F(4,6) = 2.7968 | 0.1256 | 0.6509 |
| All | Undefined Saprotroph | Null vs Primary | F(3,7) = 2.3836 | 0.1551 | 0.5053 |
| All | Undefined Saprotroph | Primary vs Seasonal | F(1,6) = 6.1293 | 0.04808 | 0.5053 |
| All | Undefined Saprotroph | Null vs Seasonal | F(4,6) = 4.6300 | 0.04785 | 0.7553 |
| All | Animal Pathogen | Null vs Primary | F(3,7) = 1.4003 | 0.3203 | 0.3751 |
| All | Animal Pathogen | Primary vs Seasonal | F(1,6) = 1.7822 | 0.2303 | 0.2290 |
| All | Animal Pathogen | Null vs Seasonal | F(4,6) = 1.6131 | 0.2858 | 0.5182 |
| All | Wood Saprotroph | Null vs Primary | F(3,7) = 3.0556 | 0.1011 | 0.5670 |
| All | Wood Saprotroph | Primary vs Seasonal | F(1,6) = 6.9923 | 0.03832 | 0.5382 |
| All | Wood Saprotroph | Null vs Seasonal | F(4,6) = 6.0015 | 0.02718 | 0.8000 |
| All | Endophyte | Null vs Primary | F(3,7) = 3.1967 | 0.09298 | 0.5781 |
| All | Endophyte | Primary vs Seasonal | F(1,6) = 4.5080 | 0.07794 | 0.4290 |
| All | Endophyte | Null vs Seasonal | F(4,6) = 4.7260 | 0.04583 | 0.7591 |
| All | Dung Saprotroph | Null vs Primary | F(3,7) = 1.8171 | 0.2318 | 0.4378 |
| All | Dung Saprotroph | Primary vs Seasonal | F(1,6) = 4.1304 | 0.08838 | 0.4077 |
| All | Dung Saprotroph | Null vs Seasonal | F(4,6) = 3.0049 | 0.1108 | 0.6670 |

(**c**) **Predictor coefficient estimates for guild richness models for all flights.**

Estimated regression coefficients (β), standard errors (SE), t-statistics, and p-values for all fixed effect predictors included in the primary and seasonal regression models of guild richness across all flights. Primary models included altitude, temperature, and windspeed, whereas seasonal models additionally incorporated NDVI as a proxy for seasonal vegetation dynamics. Results are presented for dominant, minor, and combined (“All”) trophic guild richness datasets. Positive coefficient values indicate positive associations between environmental predictors and guild richness, whereas negative values indicate inverse relationships. Statistical significance of individual predictors is indicated by the following notation: † p < 0.10; * p < 0.05; ** p < 0.01; *** p < 0.001; ns = not significant. Only analyses whose overall models were statistically significant or marginally significant and were retained in the final analysis are shown. Und. Saprotroph = Undefined Saprotroph.

| **All Flights**  **Analysis** | **Guild** | **Model** | **Predictor** | **β** | **SE** | **t** | **p-value** | **Sig.** |
| --- | --- | --- | --- | --- | --- | --- | --- | --- |
| Dominant | Animal Pathogen | Primary | Altitude | 6.540058e-05 | 5.164821e-05 | 1.26627 | 0.245925 | ns |
| Dominant | Animal Pathogen | Primary | Temperature | 0.001798 | 0.009974 | 0.180272 | 0.862047 | ns |
| Dominant | Animal Pathogen | Primary | Windspeed | -0.0214 | 0.032613 | -0.65623 | 0.532648 | ns |
| Dominant | Animal Pathogen | Seasonal | NDVI | -1.50915 | 0.397597 | -3.79568 | 0.009013 | ** |
| Dominant | Animal Pathogen | Seasonal | Altitude | 4.929173e-05 | 3.0545374-05 | 1.613722 | 0.157715 | ns |
| Dominant | Animal Pathogen | Seasonal | Temperature | 0.012365 | 0.006471 | 1.910791 | 0.104593 | ns |
| Dominant | Animal Pathogen | Seasonal | Windspeed | 0.088271 | 0.034637 | 2.548478 | 0.04358 | * |
| Dominant | Endophyte | Primary | Altitude | 0.000213 | 0.000184 | 1.158017 | 0.284836 | ns |
| Dominant | Endophyte | Primary | Temperature | -0.00118 | 0.035557 | -0.03305 | 0.97456 | ns |
| Dominant | Endophyte | Primary | Windspeed | -0.03445 | 0.116261 | -0.29629 | 0.775605 | ns |
| Dominant | Endophyte | Seasonal | NDVI | -5.31493 | 1.457644 | -3.64625 | 0.010754 | * |
| Dominant | Endophyte | Seasonal | Altitude | 0.000156 | 0.000112 | 1.397344 | 0.211799 | ns |
| Dominant | Endophyte | Seasonal | Temperature | 0.03604 | 0.023724 | 1.519112 | 0.179539 | ns |
| Dominant | Endophyte | Seasonal | Windspeed | 0.3518 | 0.126984 | 2.770432 | 0.032404 | * |
| Dominant | Dung Saprotroph | Primary | Altitude | 1.769775e-05 | 7.204854e-05 | 0.245637 | 0.81301 | ns |
| Dominant | Dung Saprotroph | Primary | Temperature | -0.03268 | 0.013914 | -2.34892 | 0.051169 | † |
| Dominant | Dung Saprotroph | Primary | Windspeed | -0.14903 | 0.045495 | -3.27577 | 0.013566 | * |
| Dominant | Dung Saprotroph | Seasonal | NDVI | -2.43855 | 0.234977 | -10.3778 | 4.686135e-05 | *** |
| Dominant | Dung Saprotroph | Seasonal | Altitude | -8.33E-06 | 1.805210e-05 | -0.46153 | 0.660676 | ns |
| Dominant | Dung Saprotroph | Seasonal | Temperature | -0.01561 | 0.003824 | -4.08124 | 0.006493 | ** |
| Dominant | Dung Saprotroph | Seasonal | Windspeed | 0.028183 | 0.02047 | 1.376792 | 0.217742 | ns |
| Minor | Plant Pathogen | Primary | Altitude | 0.000318 | 0.000213 | 1.492127 | 0.179297 | ns |
| Minor | Plant Pathogen | Primary | Temperature | -0.02426 | 0.041121 | -0.59007 | 0.57369 | ns |
| Minor | Plant Pathogen | Primary | Windspeed | -0.42525 | 0.134456 | -3.16272 | 0.015868 | * |
| Minor | Plant Pathogen | Seasonal | NDVI | -4.5931 | 2.371218 | -1.93702 | 0.100858 | ns |
| Minor | Plant Pathogen | Seasonal | Altitude | 0.000269 | 0.000182 | 1.474971 | 0.190667 | ns |
| Minor | Plant Pathogen | Seasonal | Temperature | 0.007896 | 0.038593 | 0.204596 | 0.844652 | ns |
| Minor | Plant Pathogen | Seasonal | Windspeed | -0.09146 | 0.20657 | -0.44274 | 0.673472 | ns |
| Minor | Und. Saprotroph | Primary | Altitude | 8.632273e-06 | 0.000188 | 0.045982 | 0.964609 | ns |
| Minor | Und. Saprotroph | Primary | Temperature | -0.02214 | 0.036255 | -0.61056 | 0.560782 | ns |
| Minor | Und. Saprotroph | Primary | Windspeed | -0.4164 | 0.118544 | -3.51261 | 0.009825 | ** |
| Minor | Und. Saprotroph | Seasonal | NDVI | -4.68571 | 1.855902 | -2.52476 | 0.044997 | * |
| Minor | Und. Saprotroph | Seasonal | Altitude | -4.14E-05 | 0.000143 | -0.29025 | 0.781397 | ns |
| Minor | Und. Saprotroph | Seasonal | Temperature | 0.010673 | 0.030206 | 0.353342 | 0.735915 | ns |
| Minor | Und. Saprotroph | Seasonal | Windspeed | -0.07588 | 0.161678 | -0.46931 | 0.655419 | ns |
| Minor | Wood Saprotroph | Primary | Altitude | 0.000164 | 0.000228 | 0.722462 | 0.493434 | ns |
| Minor | Wood Saprotroph | Primary | Temperature | -0.00254 | 0.043948 | -0.05787 | 0.95547 | ns |
| Minor | Wood Saprotroph | Primary | Windspeed | -0.43427 | 0.143699 | -3.02211 | 0.019329 | * |
| Minor | Wood Saprotroph | Seasonal | NDVI | -6.2199 | 1.997656 | -3.1136 | 0.020753 | * |
| Minor | Wood Saprotroph | Seasonal | Altitude | 9.801881e-05 | 0.000153 | 0.638684 | 0.546619 | ns |
| Minor | Wood Saprotroph | Seasonal | Temperature | 0.041008 | 0.032513 | 1.26127 | 0.254031 | ns |
| Minor | Wood Saprotroph | Seasonal | Windspeed | 0.017738 | 0.174027 | 0.101926 | 0.922136 | ns |
| Minor | Endophyte | Primary | Altitude | 0.00023 | 0.000129 | 1.776338 | 0.11893 | ns |
| Minor | Endophyte | Primary | Temperature | -0.03534 | 0.024963 | -1.41579 | 0.199755 | ns |
| Minor | Endophyte | Primary | Windspeed | -0.30419 | 0.081623 | -3.72674 | 0.00739 | ** |
| Minor | Endophyte | Seasonal | NDVI | -1.95525 | 1.652476 | -1.18322 | 0.281473 | ns |
| Minor | Endophyte | Seasonal | Altitude | 0.000209 | 0.000127 | 1.644276 | 0.151223 | ns |
| Minor | Endophyte | Seasonal | Temperature | -0.02165 | 0.026895 | -0.80506 | 0.4515 | ns |
| Minor | Endophyte | Seasonal | Windspeed | -0.1621 | 0.143957 | -1.12601 | 0.303176 | ns |
| All | Und. Saprotroph | Primary | Altitude | 2.547793e-05 | 0.00016 | 0.15965 | 0.877666 | ns |
| All | Und. Saprotroph | Primary | Temperature | 0.000882 | 0.030819 | 0.028614 | 0.977971 | ns |
| All | Und. Saprotroph | Primary | Windspeed | -0.25229 | 0.100771 | -2.50358 | 0.040778 | * |
| All | Und. Saprotroph | Seasonal | NDVI | -3.94513 | 1.593519 | -2.47574 | 0.048082 | * |
| All | Und. Saprotroph | Seasonal | Altitude | -1.66E-05 | 0.000122 | -0.13586 | 0.896372 | ns |
| All | Und. Saprotroph | Seasonal | Temperature | 0.028505 | 0.025935 | 1.099081 | 0.313866 | ns |
| All | Und. Saprotroph | Seasonal | Windspeed | 0.034413 | 0.13882 | 0.247893 | 0.812483 | ns |
| All | Wood Saprotroph | Primary | Altitude | 0.000171 | 0.000198 | 0.863589 | 0.41642 | ns |
| All | Wood Saprotroph | Primary | Temperature | 8.441357e-05 | 0.038322 | 0.002203 | 0.998304 | ns |
| All | Wood Saprotroph | Primary | Windspeed | -0.35986 | 0.125303 | -2.87192 | 0.023926 | * |
| All | Wood Saprotroph | Seasonal | NDVI | -5.06252 | 1.914504 | -2.6443 | 0.038319 | * |
| All | Wood Saprotroph | Seasonal | Altitude | 0.000117 | 0.000147 | 0.797714 | 0.455439 | ns |
| All | Wood Saprotroph | Seasonal | Temperature | 0.035532 | 0.03116 | 1.140306 | 0.297623 | ns |
| All | Wood Saprotroph | Seasonal | Windspeed | 0.008044 | 0.166783 | 0.048233 | 0.963096 | ns |
| All | Endophyte | Primary | Altitude | 0.000297 | 0.000177 | 1.677872 | 0.13727 | ns |
| All | Endophyte | Primary | Temperature | -0.0237 | 0.034141 | -0.69426 | 0.509896 | ns |
| All | Endophyte | Primary | Windspeed | -0.26532 | 0.111634 | -2.37673 | 0.049118 | * |
| All | Endophyte | Seasonal | NDVI | -4.02687 | 1.896596 | -2.12321 | 0.077937 | † |
| All | Endophyte | Seasonal | Altitude | 0.000254 | 0.000146 | 1.74081 | 0.132362 | ns |
| All | Endophyte | Seasonal | Temperature | 0.004493 | 0.030868 | 0.145541 | 0.88905 | ns |
| All | Endophyte | Seasonal | Windspeed | 0.027317 | 0.165223 | 0.165337 | 0.874109 | ns |

(**d**) **Comparison of fixed-effect significance between least squares and mixed-effects primary and seasonal guild richness models for all flights.** Mixed-effects models were included as sensitivity analyses to assess the potential influence of non-independence among samples collected on the same date and variance collapsed to zero in this mixed-model. **(b)** Estimated p-values for each predictor in the relevant primary or seasonal multiple linear regression model and the corresponding mixed-effects model including sampling date as a random intercept to evaluate whether predictor significance changed following inclusion of the random effect. Statistical significance: † p < 0.10; * p < 0.05; ** p < 0.01; *** p < 0.001; ns = not significant. Data are provided for all primary and seasonal mixed model date sensitivity analyses that approached statistical significance. For the majority, date variance collapsed to approximately zero. Und. Saprotroph = Undefined Saprotroph.

**Mixed-Effects sensitivity analysis comparisons (all flights).**

| **All Flights**  **Analysis** | **Guild** | **Model** | **Random effect** | **Variance** | **SD** |
| --- | --- | --- | --- | --- | --- |
| Dominant | Animal pathogen | Seasonal mixed | Date | 0 | 0 |
| Dominant | Endophyte | Seasonal mixed | Date | 0 | 0 |
| Dominant | Dung saprotroph | Primary mixed | Date | 0.009437653 | 0.097147585 |
| Dominant | Dung saprotroph | Seasonal mixed | Date | 0.000882664 | 0.029709666 |
| Minor | Plant pathogen | Primary mixed | Date | 0 | 0 |
| Minor | Plant pathogen | Seasonal mixed | Date | 0 | 0 |
| Minor | Und. saprotroph | Primary mixed | Date | 0.027559020 | 0.166009096 |
| Minor | Und. saprotroph | Seasonal mixed | Date | 0 | 0 |
| Minor | Wood saprotroph | Seasonal mixed | Date | 0 | 0 |
| Minor | Endophyte | Primary mixed | Date | 0 | 0 |
| Minor | Endophyte | Seasonal mixed | Date | 0 | 0 |
| All | Und. saprotroph | Seasonal mixed | Date | 0 | 0 |
| All | Wood saprotroph | Seasonal mixed | Date | 0 | 0 |
| All | Endophyte | Seasonal mixed | Date | 0 | 0 |

| **Analysis** | **Guild** | **Model** | **Predictor** | **LS p** | **Mixed p** | **LS Sig.** | **Mixed Sig.** |
| --- | --- | --- | --- | --- | --- | --- | --- |
| Dominant | Plant Pathogen | Primary | Altitude | 0.94567 | 0.931042 | ns | ns |
| Dominant | Plant Pathogen | Primary | Temperature | 0.549532 | 0.447313 | ns | ns |
| Dominant | Plant Pathogen | Primary | Windspeed | 0.392869 | 0.277992 | ns | ns |
| Dominant | Plant Pathogen | Seasonal | NDVI | 0.880195 | 0.566781 | ns | ns |
| Dominant | Plant Pathogen | Seasonal | Altitude | 0.967069 | 0.825652 | ns | ns |
| Dominant | Plant Pathogen | Seasonal | Temperature | 0.574119 | 0.584883 | ns | ns |
| Dominant | Plant Pathogen | Seasonal | Windspeed | 0.572353 | 0.399109 | ns | ns |
| Dominant | Und. Saprotroph | Primary | Altitude | 0.704572 | 0.630193 | ns | ns |
| Dominant | Und. Saprotroph | Primary | Temperature | 0.338986 | 0.224754 | ns | ns |
| Dominant | Und. Saprotroph | Primary | Windspeed | 0.505947 | 0.398344 | ns | ns |
| Dominant | Und. Saprotroph | Seasonal | NDVI | 0.310709 | 0.16206 | ns | ns |
| Dominant | Und. Saprotroph | Seasonal | Altitude | 0.815612 | 0.747661 | ns | ns |
| Dominant | Und. Saprotroph | Seasonal | Temperature | 0.206167 | 0.081284 | ns | † |
| Dominant | Und. Saprotroph | Seasonal | Windspeed | 0.236168 | 0.102327 | ns | ns |
| Dominant | Animal Pathogen | Primary | Altitude | 0.245925 | 0.118203 | ns | ns |
| Dominant | Animal Pathogen | Primary | Temperature | 0.862047 | 0.906478 | ns | ns |
| Dominant | Animal Pathogen | Primary | Windspeed | 0.532648 | 0.452557 | ns | ns |
| Dominant | Animal Pathogen | Seasonal | NDVI | 0.009013 | 0.000324 | ** | *** |
| Dominant | Animal Pathogen | Seasonal | Altitude | 0.157715 | 0.051416 | ns | † |
| Dominant | Animal Pathogen | Seasonal | Temperature | 0.104593 | 0.025263 | ns | * |
| Dominant | Animal Pathogen | Seasonal | Windspeed | 0.04358 | 0.005422 | * | ** |
| Dominant | Wood Saprotroph | Primary | Altitude | 0.271139 | 0.16239 | ns | ns |
| Dominant | Wood Saprotroph | Primary | Temperature | 0.914922 | 0.89209 | ns | ns |
| Dominant | Wood Saprotroph | Primary | Windspeed | 0.293196 | 0.182037 | ns | ns |
| Dominant | Wood Saprotroph | Seasonal | NDVI | 0.280838 | 0.136921 | ns | ns |
| Dominant | Wood Saprotroph | Seasonal | Altitude | 0.333285 | 0.182049 | ns | ns |
| Dominant | Wood Saprotroph | Seasonal | Temperature | 0.698163 | 0.592639 | ns | ns |
| Dominant | Wood Saprotroph | Seasonal | Windspeed | 0.742594 | 0.650477 | ns | ns |
| Dominant | Endophyte | Primary | Altitude | 0.284836 | 0.142668 | ns | ns |
| Dominant | Endophyte | Primary | Temperature | 0.97456 | 0.802471 | ns | ns |
| Dominant | Endophyte | Primary | Windspeed | 0.775605 | 0.743836 | ns | ns |
| Dominant | Endophyte | Seasonal | NDVI | 0.010754 | 0.000445 | * | *** |
| Dominant | Endophyte | Seasonal | Altitude | 0.211799 | 0.085092 | ns | † |
| Dominant | Endophyte | Seasonal | Temperature | 0.179539 | 0.064209 | ns | † |
| Dominant | Endophyte | Seasonal | Windspeed | 0.032404 | 0.003203 | * | ** |
| Dominant | Dung Saprotroph | Primary | Altitude | 0.81301 | 1 | ns | ns |
| Dominant | Dung Saprotroph | Primary | Temperature | 0.051169 | 0.996065 | † | ns |
| Dominant | Dung Saprotroph | Primary | Windspeed | 0.013566 | 0.995681 | * | ns |
| Dominant | Dung Saprotroph | Seasonal | NDVI | 4.686135e-05 | 0.683327 | *** | ns |
| Dominant | Dung Saprotroph | Seasonal | Altitude | 0.660676 | 0.999997 | ns | ns |
| Dominant | Dung Saprotroph | Seasonal | Temperature | 0.006493 | 0.733311 | ** | ns |
| Dominant | Dung Saprotroph | Seasonal | Windspeed | 0.217742 | 0.861083 | ns | ns |
| Minor | Plant Pathogen | Primary | Altitude | 0.179297 | 0.088242 | ns | † |
| Minor | Plant Pathogen | Primary | Temperature | 0.57369 | 0.474978 | ns | ns |
| Minor | Plant Pathogen | Primary | Windspeed | 0.015868 | 0.002216 | * | ** |
| Minor | Plant Pathogen | Seasonal | NDVI | 0.100858 | 0.023713 | ns | * |
| Minor | Plant Pathogen | Seasonal | Altitude | 0.190667 | 0.071154 | ns | † |
| Minor | Plant Pathogen | Seasonal | Temperature | 0.844652 | 0.786898 | ns | ns |
| Minor | Plant Pathogen | Seasonal | Windspeed | 0.673472 | 0.561006 | ns | ns |
| Minor | Und. Saprotroph | Primary | Altitude | 0.964609 | 0.977474 | ns | ns |
| Minor | Und. Saprotroph | Primary | Temperature | 0.560782 | 0.458091 | ns | ns |
| Minor | Und. Saprotroph | Primary | Windspeed | 0.009825 | 0.001795 | ** | ** |
| Minor | Und. Saprotroph | Seasonal | NDVI | 0.044997 | 0.005738 | * | ** |
| Minor | Und. Saprotroph | Seasonal | Altitude | 0.781397 | 0.70183 | ns | ns |
| Minor | Und. Saprotroph | Seasonal | Temperature | 0.735915 | 0.641713 | ns | ns |
| Minor | Und. Saprotroph | Seasonal | Windspeed | 0.655419 | 0.538133 | ns | ns |
| Minor | Animal Pathogen | Primary | Altitude | 0.369515 | 0.254551 | ns | ns |
| Minor | Animal Pathogen | Primary | Temperature | 0.432292 | 0.318734 | ns | ns |
| Minor | Animal Pathogen | Primary | Windspeed | 0.187482 | 0.094287 | ns | † |
| Minor | Animal Pathogen | Seasonal | NDVI | 0.410547 | 0.256317 | ns | ns |
| Minor | Animal Pathogen | Seasonal | Altitude | 0.447853 | 0.295107 | ns | ns |
| Minor | Animal Pathogen | Seasonal | Temperature | 0.731312 | 0.635686 | ns | ns |
| Minor | Animal Pathogen | Seasonal | Windspeed | 0.957807 | 0.941812 | ns | ns |
| Minor | Wood Saprotroph | Primary | Altitude | 0.493434 | 0.4195 | ns | ns |
| Minor | Wood Saprotroph | Primary | Temperature | 0.95547 | 0.789382 | ns | ns |
| Minor | Wood Saprotroph | Primary | Windspeed | 0.019329 | 0.007215 | * | ** |
| Minor | Wood Saprotroph | Seasonal | NDVI | 0.020753 | 0.001446 | * | ** |
| Minor | Wood Saprotroph | Seasonal | Altitude | 0.546619 | 0.405622 | ns | ns |
| Minor | Wood Saprotroph | Seasonal | Temperature | 0.254031 | 0.11571 | ns | ns |
| Minor | Wood Saprotroph | Seasonal | Windspeed | 0.922136 | 0.892728 | ns | ns |
| Minor | Endophyte | Primary | Altitude | 0.11893 | 0.047797 | ns | * |
| Minor | Endophyte | Primary | Temperature | 0.199755 | 0.103574 | ns | ns |
| Minor | Endophyte | Primary | Windspeed | 0.00739 | 0.000681 | ** | *** |
| Minor | Endophyte | Seasonal | NDVI | 0.281473 | 0.137439 | ns | ns |
| Minor | Endophyte | Seasonal | Altitude | 0.151223 | 0.04783 | ns | * |
| Minor | Endophyte | Seasonal | Temperature | 0.4515 | 0.298992 | ns | ns |
| Minor | Endophyte | Seasonal | Windspeed | 0.303176 | 0.155576 | ns | ns |
| Minor | Dung Saprotroph | Primary | Altitude | 0.450877 | 0.338414 | ns | ns |
| Minor | Dung Saprotroph | Primary | Temperature | 0.156011 | 0.071741 | ns | † |
| Minor | Dung Saprotroph | Primary | Windspeed | 0.179789 | 0.088602 | ns | † |
| Minor | Dung Saprotroph | Seasonal | NDVI | 0.330557 | 0.17959 | ns | ns |
| Minor | Dung Saprotroph | Seasonal | Altitude | 0.38138 | 0.22727 | ns | ns |
| Minor | Dung Saprotroph | Seasonal | Temperature | 0.105815 | 0.025779 | ns | * |
| Minor | Dung Saprotroph | Seasonal | Windspeed | 0.958535 | 0.942815 | ns | ns |
| All | Plant Pathogen | Primary | Altitude | 0.359559 | 0.244725 | ns | ns |
| All | Plant Pathogen | Primary | Temperature | 0.91234 | 0.888824 | ns | ns |
| All | Plant Pathogen | Primary | Windspeed | 0.0373 | 0.008233 | * | ** |
| All | Plant Pathogen | Seasonal | NDVI | 0.187929 | 0.06942 | ns | † |
| All | Plant Pathogen | Seasonal | Altitude | 0.430595 | 0.276942 | ns | ns |
| All | Plant Pathogen | Seasonal | Temperature | 0.616735 | 0.48991 | ns | ns |
| All | Plant Pathogen | Seasonal | Windspeed | 0.780066 | 0.700058 | ns | ns |
| All | Und. Saprotroph | Primary | Altitude | 0.877666 | 0.986573 | ns | ns |
| All | Und. Saprotroph | Primary | Temperature | 0.977971 | 0.860764 | ns | ns |
| All | Und. Saprotroph | Primary | Windspeed | 0.040778 | 0.020946 | * | * |
| All | Und. Saprotroph | Seasonal | NDVI | 0.048082 | 0.006453 | * | ** |
| All | Und. Saprotroph | Seasonal | Altitude | 0.896372 | 0.857391 | ns | ns |
| All | Und. Saprotroph | Seasonal | Temperature | 0.313866 | 0.164805 | ns | ns |
| All | Und. Saprotroph | Seasonal | Windspeed | 0.812483 | 0.743451 | ns | ns |
| All | Animal Pathogen | Primary | Altitude | 0.29606 | 0.184635 | ns | ns |
| All | Animal Pathogen | Primary | Temperature | 0.624861 | 0.534671 | ns | ns |
| All | Animal Pathogen | Primary | Windspeed | 0.17133 | 0.082479 | ns | † |
| All | Animal Pathogen | Seasonal | NDVI | 0.230304 | 0.09807 | ns | † |
| All | Animal Pathogen | Seasonal | Altitude | 0.358838 | 0.205644 | ns | ns |
| All | Animal Pathogen | Seasonal | Temperature | 0.93302 | 0.907688 | ns | ns |
| All | Animal Pathogen | Seasonal | Windspeed | 0.82759 | 0.76381 | ns | ns |
| All | Wood Saprotroph | Primary | Altitude | 0.41642 | 0.321582 | ns | ns |
| All | Wood Saprotroph | Primary | Temperature | 0.998304 | 0.898287 | ns | ns |
| All | Wood Saprotroph | Primary | Windspeed | 0.023926 | 0.006826 | * | ** |
| All | Wood Saprotroph | Seasonal | NDVI | 0.038319 | 0.004316 | * | ** |
| All | Wood Saprotroph | Seasonal | Altitude | 0.455439 | 0.303206 | ns | ns |
| All | Wood Saprotroph | Seasonal | Temperature | 0.297623 | 0.150858 | ns | ns |
| All | Wood Saprotroph | Seasonal | Windspeed | 0.963096 | 0.949101 | ns | ns |
| All | Endophyte | Primary | Altitude | 0.13727 | 0.059258 | ns | † |
| All | Endophyte | Primary | Temperature | 0.509896 | 0.402728 | ns | ns |
| All | Endophyte | Primary | Windspeed | 0.049118 | 0.012533 | * | * |
| All | Endophyte | Seasonal | NDVI | 0.077937 | 0.01511 | † | * |
| All | Endophyte | Seasonal | Altitude | 0.132362 | 0.03801 | ns | * |
| All | Endophyte | Seasonal | Temperature | 0.88905 | 0.847372 | ns | ns |
| All | Endophyte | Seasonal | Windspeed | 0.874109 | 0.826964 | ns | ns |
| All | Dung Saprotroph | Primary | Altitude | 0.720354 | 0.596347 | ns | ns |
| All | Dung Saprotroph | Primary | Temperature | 0.458148 | 0.422796 | ns | ns |
| All | Dung Saprotroph | Primary | Windspeed | 0.078191 | 0.031935 | † | * |
| All | Dung Saprotroph | Seasonal | NDVI | 0.088376 | 0.01883 | † | * |
| All | Dung Saprotroph | Seasonal | Altitude | 0.494909 | 0.346436 | ns | ns |
| All | Dung Saprotroph | Seasonal | Temperature | 0.134929 | 0.039293 | ns | * |
| All | Dung Saprotroph | Seasonal | Windspeed | 0.754218 | 0.665785 | ns | ns |

LS = Least-squares; Mixed = Mixed-model.

(**e**) **All flights guild richness model assumption diagnostics for guilds containing total (all), dominant, and minor OTUs.** Statistical significance: † p < 0.10; * p < 0.05; ** p < 0.01; *** p < 0.001; ns = not significant. Data are provided for all primary and seasonal models that approached statistical significance.

| **Model** | **Predictor** | | **Variance Inflation Factor (VIF)** | | | | |  |  |
| --- | --- | --- | --- | --- | --- | --- | --- | --- | --- |
| Primary | Altitude | | 1.133578 | | | | |  |  |
| Primary | Temperature | | 1.141962 | | | | |  |  |
| Primary | Windspeed | | 1.179243 | | | | |  |  |
| Seasonal | NDVI | | 4.317768 | | | | |  |  |
| Seasonal | Altitude | | 1.155892 | | | | |  |  |
| Seasonal | Temperature | | 1.401315 | | | | |  |  |
| Seasonal | Windspeed | | 3.877723 | | | | |  |  |
| **All Flights**  **Analysis** | | **Guild** | | **Model** | **Temporal Autocorrelation Test** | **Statistic** | **p-value** | | **Sig.** |
| Dominant | | Animal Pathogen | | Seasonal | Ljung–Box | 0.000674589 | 0.979278994 | | ns |
| Dominant | | Endophyte | | Seasonal | Ljung–Box | 0.747799618 | 0.387173780 | | ns |
| Dominant | | Dung Saprotroph | | Primary | Ljung–Box | 4.697878331 | 0.030199876 | | * |
| Dominant | | Dung Saprotroph | | Seasonal | Ljung–Box | 0.064962377 | 0.798817962 | | ns |
| Minor | | Plant Pathogen | | Primary | Ljung–Box | 0.810720198 | 0.367907410 | | ns |
| Minor | | Plant Pathogen | | Seasonal | Ljung–Box | 0.000401765 | 0.984008214 | | ns |
| Minor | | Undefined Saprotroph | | Primary | Ljung–Box | 2.572754636 | 0.108718269 | | ns |
| Minor | | Undefined Saprotroph | | Seasonal | Ljung–Box | 0.092599327 | 0.760898297 | | ns |
| Minor | | Wood Saprotroph | | Seasonal | Ljung–Box | 0.068852214 | 0.793015344 | | ns |
| Minor | | Endophyte | | Primary | Ljung–Box | 0.316055663 | 0.573987745 | | ns |
| Minor | | Endophyte | | Seasonal | Ljung–Box | 0.000314996 | 0.985839782 | | ns |
| All | | Undefined Saprotroph | | Seasonal | Ljung–Box | 0.275477779 | 0.599680677 | | ns |
| All | | Wood Saprotroph | | Seasonal | Ljung–Box | 0.370284586 | 0.542849185 | | ns |
| All | | Endophyte | | Seasonal | Ljung–Box | 0.001722569 | 0.966894228 | | ns |

**Table 1.9 Guild Richness Georgia Flights Only (Excluding FLT7)**

(**a**) **Guild richness overall model comparisons excluding FLT7.**

Summary statistics for null, primary, and seasonal least squares regression models predicting trophic guild richness after exclusion of sample FLT7. Reported values include model R², adjusted R² (Adj. R²), Akaike Information Criterion (AIC), residual standard error (ResSE), F-statistic, model p-value, and significance classification. Primary models included altitude, temperature, and windspeed predictors; seasonal models additionally included NDVI. The best statistically supported significant models are in **bold**. Significance notations: † p < 0.10; * p < 0.05; ** p < 0.01; *** p < 0.001; ns = not significant. Und. Saprotroph = Undefined Saprotroph.

| **Excluding FLT7**  **Analysis** | **Guild** | **Model** | **R²** | **Adj. R²** | **AIC** | **ResSE** | **F** | **p** | **Sig.** |
| --- | --- | --- | --- | --- | --- | --- | --- | --- | --- |
| Dominant | Plant Pathogen | Null | 0 | 0 | -3.62951 | 0.174168 |  |  | — |
| Dominant | Plant Pathogen | **Primary** | 0.732831 | 0.599247 | -10.8283 | 0.110257 | 5.485909 | 0.037284 | * |
| Dominant | Plant Pathogen | Seasonal | 0.737278 | 0.527100 | -8.99608 | 0.119772 | 3.507873 | 0.100589 | ns |
| Dominant | Und. Saprotroph | Null | 0 | 0 | 7.549802 | 0.304596 |  |  | — |
| Dominant | Und. Saprotroph | Primary | 0.690881 | 0.536322 | 1.80951 | 0.207412 | 4.470002 | 0.056589 | † |
| Dominant | Und. Saprotroph | Seasonal | 0.752363 | 0.554253 | 1.591903 | 0.203362 | 3.797704 | 0.087916 | † |
| Dominant | Animal Pathogen | Null | 0 | 0 | -8.14569 | 0.138964 |  |  | — |
| Dominant | Animal Pathogen | Primary | 0.231274 | -0.153090 | -4.77591 | 0.149222 | 0.601709 | 0.637293 | ns |
| Dominant | Animal Pathogen | Seasonal | 0.770843 | 0.587518 | -14.8792 | 0.089249 | 4.204781 | 0.073582 | † |
| Dominant | Wood Saprotroph | Null | 0 | 0 | 7.501837 | 0.303867 |  |  | — |
| Dominant | Wood Saprotroph | Primary | 0.221425 | -0.167860 | 10.99893 | 0.328382 | 0.568796 | 0.655698 | ns |
| Dominant | Wood Saprotroph | Seasonal | 0.361413 | -0.149460 | 11.01687 | 0.325784 | 0.707446 | 0.620313 | ns |
| Dominant | Endophyte | Null | 0 | 0 | 17.31678 | 0.496377 |  |  | — |
| Dominant | Endophyte | Primary | 0.188432 | -0.217350 | 21.22891 | 0.547671 | 0.464365 | 0.717616 | ns |
| Dominant | Endophyte | Seasonal | 0.742139 | 0.535850 | 11.76343 | 0.338174 | 3.597574 | 0.096410 | † |
| Dominant | Dung Saprotroph | Null | 0 | 0 | 9.442035 | 0.334822 |  |  | — |
| Dominant | Dung Saprotroph | Primary | 0.721952 | 0.582928 | 2.64241 | 0.216232 | 5.193010 | 0.041810 | * |
| Dominant | Dung Saprotroph | **Seasonal** | 1.000000 | 1.000000 | -748.631 | 1.04083E-17 | 2.328344E+33 | <0.0001 | *** |
| Minor | Plant Pathogen | Null | 0 | 0 | 24.70898 | 0.718342 |  |  | — |
| Minor | Plant Pathogen | Primary | 0.550365 | 0.325548 | 22.71578 | 0.589939 | 2.448054 | 0.161552 | ns |
| Minor | Plant Pathogen | Seasonal | 0.718068 | 0.492522 | 20.04809 | 0.511729 | 3.183691 | 0.117969 | ns |
| Minor | Und. Saprotroph | Null | 0 | 0 | 21.96973 | 0.626396 |  |  | — |
| Minor | Und. Saprotroph | Primary | 0.482213 | 0.223319 | 21.38782 | 0.552040 | 1.862591 | 0.236737 | ns |
| Minor | Und. Saprotroph | Seasonal | 0.741763 | 0.535174 | 16.43095 | 0.427066 | 3.590516 | 0.096730 | † |
| Minor | Animal Pathogen | Null | 0 | 0 | 18.65209 | 0.530649 |  |  | — |
| Minor | Animal Pathogen | Primary | 0.632440 | 0.448661 | 14.64339 | 0.394019 | 3.441295 | 0.092341 | † |
| Minor | Animal Pathogen | Seasonal | 0.669824 | 0.405684 | 15.57078 | 0.409088 | 2.535864 | 0.167539 | ns |
| Minor | Wood Saprotroph | Null | 0 | 0 | 22.73806 | 0.650928 |  |  | — |
| Minor | Wood Saprotroph | Primary | 0.322806 | -0.015790 | 24.84008 | 0.656048 | 0.953364 | 0.472590 | ns |
| Minor | Wood Saprotroph | Seasonal | 0.735758 | 0.524364 | 17.42917 | 0.448922 | 3.480504 | 0.101914 | ns |
| Minor | Endophyte | Null | 0 | 0 | 19.28459 | 0.547699 |  |  | — |
| Minor | Endophyte | **Primary** | 0.717437 | 0.576156 | 12.64604 | 0.356570 | 5.078078 | 0.043785 | * |
| Minor | Endophyte | Seasonal | 0.764170 | 0.575506 | 12.83816 | 0.356844 | 4.050427 | 0.078606 | † |
| Minor | Dung Saprotroph | Null | 0 | 0 | 14.90225 | 0.439927 |  |  | — |
| Minor | Dung Saprotroph | Primary | 0.255825 | -0.116260 | 17.94745 | 0.464798 | 0.687541 | 0.591809 | ns |
| Minor | Dung Saprotroph | Seasonal | 0.375596 | -0.123930 | 18.19268 | 0.466391 | 0.751908 | 0.597366 | ns |
| All | Plant Pathogen | Null | 0 | 0 | 8.421809 | 0.318171 |  |  | — |
| All | Plant Pathogen | Primary | 0.480314 | 0.220471 | 7.876501 | 0.280916 | 1.848479 | 0.239064 | ns |
| All | Plant Pathogen | Seasonal | 0.636914 | 0.346445 | 6.290654 | 0.257218 | 2.192710 | 0.205924 | ns |
| All | Und. Saprotroph | Null | 0 | 0 | 12.55233 | 0.391159 |  |  | — |
| All | Und. Saprotroph | Primary | 0.243317 | -0.135020 | 15.76423 | 0.416731 | 0.643115 | 0.614900 | ns |
| All | Und. Saprotroph | Seasonal | 0.649475 | 0.369054 | 10.06911 | 0.310706 | 2.316075 | 0.190858 | ns |
| All | Animal Pathogen | Null | 0 | 0 | 9.531198 | 0.336318 |  |  | — |
| All | Animal Pathogen | Primary | 0.513909 | 0.270864 | 8.317601 | 0.287180 | 2.114457 | 0.199755 | ns |
| All | Animal Pathogen | Seasonal | 0.628257 | 0.330862 | 7.635680 | 0.275111 | 2.112536 | 0.216595 | ns |
| All | Wood Saprotroph | Null | 0 | 0 | 19.75613 | 0.560766 |  |  | — |
| All | Wood Saprotroph | Primary | 0.305286 | -0.042070 | 22.11357 | 0.572440 | 0.878884 | 0.502903 | ns |
| All | Wood Saprotroph | Seasonal | 0.670873 | 0.407571 | 16.64302 | 0.431618 | 2.547925 | 0.166375 | ns |
| All | Endophyte | Null | 0 | 0 | 20.78407 | 0.590341 |  |  | — |
| All | Endophyte | Primary | 0.532910 | 0.299366 | 19.17173 | 0.494139 | 2.281834 | 0.179264 | ns |
| All | Endophyte | Seasonal | 0.728389 | 0.511101 | 15.75022 | 0.412774 | 3.352174 | 0.108459 | ns |
| All | Dung Saprotroph | Null | 0 | 0 | 13.86323 | 0.417656 |  |  | — |
| All | Dung Saprotroph | Primary | 0.180213 | -0.229680 | 17.87612 | 0.463143 | 0.439657 | 0.733022 | ns |
| All | Dung Saprotroph | Seasonal | 0.549889 | 0.189800 | 13.88062 | 0.375937 | 1.527091 | 0.322784 | ns |

(**b**)**. ANOVA comparisons for models of guild richness excluding FLT7.**
Analysis of variance (ANOVA) comparisons among null, primary, and seasonal trophic guild richness models after excluding sample FLT7. Comparisons include Null vs Primary, Primary vs Seasonal, and Null vs Seasonal models. Reported values include F-statistics, p-values, and effect sizes (η²). Larger η² values indicate greater explanatory improvement associated with the more complex model. Und. Saprotroph = Undefined Saprotroph.

| **Excluding FLT7**  **Analysis** | **Guild** | **Comparison** | **F-stat** | **p** | **η²** |
| --- | --- | --- | --- | --- | --- |
| Dominant | Plant Pathogen | Null vs Primary | F(3,6) = 5.4859 | 0.03728 | 0.7328 |
| Dominant | Plant Pathogen | Primary vs Seasonal | F(1,5) = 0.0846 | 0.7828 | 0.0166 |
| Dominant | Plant Pathogen | Null vs Seasonal | F(4,5) = 3.5079 | 0.1006 | 0.7373 |
| Dominant | Und. Saprotroph | Null vs Primary | F(3,6) = 4.47 | 0.05659 | 0.6909 |
| Dominant | Und. Saprotroph | Primary vs Seasonal | F(1,5) = 1.2414 | 0.3159 | 0.1989 |
| Dominant | Und. Saprotroph | Null vs Seasonal | F(4,5) = 3.7977 | 0.08792 | 0.7524 |
| Dominant | Animal Pathogen | Null vs Primary | F(3,6) = 0.6017 | 0.6373 | 0.2313 |
| Dominant | Animal Pathogen | Primary vs Seasonal | F(1,5) = 11.7729 | 0.01861 | 0.7019 |
| Dominant | Animal Pathogen | Null vs Seasonal | F(4,5) = 4.2048 | 0.07358 | 0.7708 |
| Dominant | Wood Saprotroph | Null vs Primary | F(3,6) = 0.5688 | 0.6557 | 0.2214 |
| Dominant | Wood Saprotroph | Primary vs Seasonal | F(1,5) = 1.0961 | 0.3431 | 0.1798 |
| Dominant | Wood Saprotroph | Null vs Seasonal | F(4,5) = 0.7074 | 0.6203 | 0.3614 |
| Dominant | Endophyte | Null vs Primary | F(3,6) = 0.4644 | 0.7176 | 0.1884 |
| Dominant | Endophyte | Primary vs Seasonal | F(1,5) = 10.7365 | 0.02204 | 0.6823 |
| Dominant | Endophyte | Null vs Seasonal | F(4,5) = 3.5976 | 0.09641 | 0.7421 |
| Dominant | Dung Saprotroph | Null vs Primary | F(3,6) = 5.193 | 0.04181 | 0.7220 |
| Dominant | Dung Saprotroph | Primary vs Seasonal | F(1,5) = 2.589e+33 | 5.562e-83 | 1 |
| Dominant | Dung Saprotroph | Null vs Seasonal | F(4,5) = 2.328e+33 | 2.337e-83 | 1 |
| Minor | Plant Pathogen | Null vs Primary | F(3,6) = 2.4481 | 0.1616 | 0.5504 |
| Minor | Plant Pathogen | Primary vs Seasonal | F(1,5) = 2.9742 | 0.1452 | 0.3730 |
| Minor | Plant Pathogen | Null vs Seasonal | F(4,5) = 3.1837 | 0.1180 | 0.7181 |
| Minor | Und. Saprotroph | Null vs Primary | F(3,6) = 1.8626 | 0.2367 | 0.4822 |
| Minor | Und. Saprotroph | Primary vs Seasonal | F(1,5) = 5.0254 | 0.07505 | 0.5013 |
| Minor | Und. Saprotroph | Null vs Seasonal | F(4,5) = 3.5905 | 0.09673 | 0.7418 |
| Minor | Animal Pathogen | Null vs Primary | F(3,6) = 3.4413 | 0.09234 | 0.6324 |
| Minor | Animal Pathogen | Primary vs Seasonal | F(1,5) = 0.5661 | 0.4857 | 0.1017 |
| Minor | Animal Pathogen | Null vs Seasonal | F(4,5) = 2.5359 | 0.1675 | 0.6698 |
| Minor | Wood Saprotroph | Null vs Primary | F(3,6) = 0.9534 | 0.4726 | 0.3228 |
| Minor | Wood Saprotroph | Primary vs Seasonal | F(1,5) = 7.8139 | 0.0382 | 0.6098 |
| Minor | Wood Saprotroph | Null vs Seasonal | F(4,5) = 3.4805 | 0.1019 | 0.7358 |
| Minor | Endophyte | Null vs Primary | F(3,6) = 5.0781 | 0.04378 | 0.7174 |
| Minor | Endophyte | Primary vs Seasonal | F(1,5) = 0.9908 | 0.3652 | 0.1654 |
| Minor | Endophyte | Null vs Seasonal | F(4,5) = 4.0504 | 0.07861 | 0.7642 |
| Minor | Dung Saprotroph | Null vs Primary | F(3,6) = 0.6875 | 0.5918 | 0.2558 |
| Minor | Dung Saprotroph | Primary vs Seasonal | F(1,5) = 0.9591 | 0.3724 | 0.1609 |
| Minor | Dung Saprotroph | Null vs Seasonal | F(4,5) = 0.7519 | 0.5974 | 0.3756 |
| All | Plant Pathogen | Null vs Primary | F(3,6) = 1.8485 | 0.2391 | 0.4803 |
| All | Plant Pathogen | Primary vs Seasonal | F(1,5) = 2.1565 | 0.2019 | 0.3013 |
| All | Plant Pathogen | Null vs Seasonal | F(4,5) = 2.1927 | 0.2059 | 0.6369 |
| All | Und. Saprotroph | Null vs Primary | F(3,6) = 0.6431 | 0.6149 | 0.2433 |
| All | Und. Saprotroph | Primary vs Seasonal | F(1,5) = 5.7936 | 0.06109 | 0.5368 |
| All | Und. Saprotroph | Null vs Seasonal | F(4,5) = 2.3161 | 0.1909 | 0.6495 |
| All | Animal Pathogen | Null vs Primary | F(3,6) = 2.1145 | 0.1998 | 0.5139 |
| All | Animal Pathogen | Primary vs Seasonal | F(1,5) = 1.538 | 0.2699 | 0.2352 |
| All | Animal Pathogen | Null vs Seasonal | F(4,5) = 2.1125 | 0.2166 | 0.6283 |
| All | Wood Saprotroph | Null vs Primary | F(3,6) = 0.8789 | 0.5029 | 0.3053 |
| All | Wood Saprotroph | Primary vs Seasonal | F(1,5) = 5.5539 | 0.06502 | 0.5262 |
| All | Wood Saprotroph | Null vs Seasonal | F(4,5) = 2.5479 | 0.1664 | 0.6709 |
| All | Endophyte | Null vs Primary | F(3,6) = 2.2818 | 0.1793 | 0.5329 |
| All | Endophyte | Primary vs Seasonal | F(1,5) = 3.5985 | 0.1163 | 0.4185 |
| All | Endophyte | Null vs Seasonal | F(4,5) = 3.3522 | 0.1085 | 0.7284 |
| All | Dung Saprotroph | Null vs Primary | F(3,6) = 0.4397 | 0.7330 | 0.1802 |
| All | Dung Saprotroph | Primary vs Seasonal | F(1,5) = 4.1065 | 0.09856 | 0.4509 |
| All | Dung Saprotroph | Null vs Seasonal | F(4,5) = 1.5271 | 0.3228 | 0.5499 |

(**c**) **Predictor coefficient estimates for guild richness models excluding FLT7.**

Estimated regression coefficients (β), standard errors (SE), t-statistics, and p-values for all fixed effect predictors included in the primary and seasonal regression models of guild richness after exclusion of sample FLT7. Primary models included altitude, temperature, and windspeed, whereas seasonal models additionally incorporated NDVI as a proxy for seasonal vegetation dynamics. Results are presented for dominant, minor, and combined (“All”) trophic guild richness datasets. Positive coefficient values indicate positive associations between environmental predictors and guild richness, whereas negative values indicate inverse relationships. Statistical significance of individual predictors is indicated by the following notation: † p < 0.10; * p < 0.05; ** p < 0.01; *** p < 0.001; ns = not significant. Only analyses whose overall models were statistically significant or marginally significant and were retained in the final analysis excluding FLT7 are shown. Und. Saprotroph = Undefined Saprotroph.

| **Excluding**  **FLT7**  **Analysis** | **Guild** | **Model** | **Predictor** | **β** | **SE** | **t** | **p value** | **Sig.** | |
| --- | --- | --- | --- | --- | --- | --- | --- | --- | --- |
| Dominant | Plant Pathogen | Primary | Altitude | -2.44E-05 | 4.047901e-05 | -0.60197 | 0.569226 | ns |  |
| Dominant | Plant Pathogen | Primary | Temperature | -0.02456 | 0.012084 | -2.03272 | 0.08833 | † |  |
| Dominant | Plant Pathogen | Primary | Windspeed | 0.251347 | 0.067187 | 3.741016 | 0.009611 | ** |  |
| Dominant | Plant Pathogen | Seasonal | NDVI | 0.166896 | 0.573742 | 0.290891 | 0.782815 | ns |  |
| Dominant | Plant Pathogen | Seasonal | Altitude | -2.30E-05 | 4.420849e-05 | -0.52114 | 0.624517 | ns |  |
| Dominant | Plant Pathogen | Seasonal | Temperature | -0.02624 | 0.014335 | -1.83045 | 0.126695 | ns |  |
| Dominant | Plant Pathogen | Seasonal | Windspeed | 0.242596 | 0.078941 | 3.073117 | 0.027685 | * |  |
| Dominant | Und. Saprotroph | Primary | Altitude | -3.58E-06 | 7.614750e-05 | -0.047 | 0.964036 | ns |  |
| Dominant | Und. Saprotroph | Primary | Temperature | -0.03016 | 0.022732 | -1.32697 | 0.232781 | ns |  |
| Dominant | Und. Saprotroph | Primary | Windspeed | 0.390461 | 0.126389 | 3.089353 | 0.021406 | * |  |
| Dominant | Und. Saprotroph | Seasonal | NDVI | -1.08538 | 0.974163 | -1.11416 | 0.31588 | ns |  |
| Dominant | Und. Saprotroph | Seasonal | Altitude | -1.22E-05 | 7.506212e-05 | -0.16277 | 0.877076 | ns |  |
| Dominant | Und. Saprotroph | Seasonal | Temperature | -0.01927 | 0.024339 | -0.79171 | 0.464415 | ns |  |
| Dominant | Und. Saprotroph | Seasonal | Windspeed | 0.447371 | 0.134035 | 3.337705 | 0.020604 | * |  |
| Dominant | Animal Pathogen | Primary | Altitude | 5.740121e-05 | 5.478418e-05 | 1.04777 | 0.335105 | ns |  |
| Dominant | Animal Pathogen | Primary | Temperature | -0.00715 | 0.016354 | -0.43703 | 0.677382 | ns |  |
| Dominant | Animal Pathogen | Primary | Windspeed | 0.038225 | 0.09093 | 0.420374 | 0.688852 | ns |  |
| Dominant | Animal Pathogen | Seasonal | NDVI | -1.46693 | 0.42753 | -3.43117 | 0.018612 | * |  |
| Dominant | Animal Pathogen | Seasonal | Altitude | 4.572603e-05 | 3.294243e-05 | 1.388059 | 0.223789 | ns |  |
| Dominant | Animal Pathogen | Seasonal | Temperature | 0.007578 | 0.010682 | 0.709443 | 0.509752 | ns |  |
| Dominant | Animal Pathogen | Seasonal | Windspeed | 0.115141 | 0.058824 | 1.957378 | 0.107649 | ns |  |
| Dominant | Endophyte | Primary | Altitude | 0.000198 | 0.000201 | 0.985468 | 0.362451 | ns |  |
| Dominant | Endophyte | Primary | Temperature | -0.01802 | 0.060023 | -0.30026 | 0.774112 | ns |  |
| Dominant | Endophyte | Primary | Windspeed | 0.077852 | 0.333731 | 0.233276 | 0.823302 | ns |  |
| Dominant | Endophyte | Seasonal | NDVI | -5.30806 | 1.619957 | -3.27667 | 0.022036 | * |  |
| Dominant | Endophyte | Seasonal | Altitude | 0.000156 | 0.000125 | 1.248968 | 0.266963 | ns |  |
| Dominant | Endophyte | Seasonal | Temperature | 0.035261 | 0.040473 | 0.871203 | 0.423492 | ns |  |
| Dominant | Endophyte | Seasonal | Windspeed | 0.356172 | 0.222891 | 1.597966 | 0.170942 | ns |  |
| Dominant | Dung Saprotroph | Primary | Altitude | 2.019354e-05 | 7.938562e-05 | 0.254373 | 0.807701 | ns |  |
| Dominant | Dung Saprotroph | Primary | Temperature | -0.02989 | 0.023698 | -1.26134 | 0.254007 | ns |  |
| Dominant | Dung Saprotroph | Primary | Windspeed | -0.16763 | 0.131764 | -1.27224 | 0.250368 | ns |  |
| Dominant | Dung Saprotroph | Seasonal | NDVI | -2.53722 | 4.985907e-17 | -5.1E+16 | 5.562083e-83 | *** |  |
| Dominant | Dung Saprotroph | Seasonal | Altitude | -2.42E-21 | 3.841788e-21 | -0.62915 | 0.556872 | ns |  |
| Dominant | Dung Saprotroph | Seasonal | Temperature | -0.00442 | 1.245692e-18 | -3.6E+15 | 3.363907e-77 | *** |  |
| Dominant | Dung Saprotroph | Seasonal | Windspeed | -0.0346 | 6.860130e-18 | -5E+15 | 5.815581e-78 | *** |  |
| Minor | Und. Saprotroph | Primary | Altitude | -1.33E-05 | 0.000203 | -0.06559 | 0.949832 | ns |  |
| Minor | Und. Saprotroph | Primary | Temperature | -0.04666 | 0.060502 | -0.77113 | 0.469898 | ns |  |
| Minor | Und. Saprotroph | Primary | Windspeed | -0.25296 | 0.336393 | -0.75198 | 0.480513 | ns |  |
| Minor | Und. Saprotroph | Seasonal | NDVI | -4.5861 | 2.045773 | -2.24175 | 0.07505 | † |  |
| Minor | Und. Saprotroph | Seasonal | Altitude | -4.98E-05 | 0.000158 | -0.31589 | 0.764847 | ns |  |
| Minor | Und. Saprotroph | Seasonal | Temperature | -0.00062 | 0.051112 | -0.01211 | 0.990805 | ns |  |
| Minor | Und. Saprotroph | Seasonal | Windspeed | -0.01249 | 0.281479 | -0.04439 | 0.966312 | ns |  |
| Minor | Animal Pathogen | Primary | Altitude | 0.000105 | 0.000145 | 0.726972 | 0.494618 | ns |  |
| Minor | Animal Pathogen | Primary | Temperature | -0.11572 | 0.043183 | -2.67984 | 0.036544 | * |  |
| Minor | Animal Pathogen | Primary | Windspeed | 0.393987 | 0.240101 | 1.64092 | 0.151923 | ns |  |
| Minor | Animal Pathogen | Seasonal | NDVI | -1.47446 | 1.959653 | -0.75241 | 0.485696 | ns |  |
| Minor | Animal Pathogen | Seasonal | Altitude | 9.342658e-05 | 0.000151 | 0.618731 | 0.56319 | ns |  |
| Minor | Animal Pathogen | Seasonal | Temperature | -0.10092 | 0.048961 | -2.06133 | 0.094273 | † |  |
| Minor | Animal Pathogen | Seasonal | Windspeed | 0.471298 | 0.269629 | 1.747946 | 0.140895 | ns |  |
| Minor | Endophyte | Primary | Altitude | 0.000201 | 0.000131 | 1.534344 | 0.175843 | ns |  |
| Minor | Endophyte | Primary | Temperature | -0.0675 | 0.039079 | -1.72721 | 0.134874 | ns |  |
| Minor | Endophyte | Primary | Windspeed | -0.08985 | 0.217281 | -0.41353 | 0.693595 | ns |  |
| Minor | Endophyte | Seasonal | NDVI | -1.70152 | 1.709389 | -0.99539 | 0.365246 | ns |  |
| Minor | Endophyte | Seasonal | Altitude | 0.000187 | 0.000132 | 1.422151 | 0.214252 | ns |  |
| Minor | Endophyte | Seasonal | Temperature | -0.05042 | 0.042708 | -1.18053 | 0.290885 | ns |  |
| Minor | Endophyte | Seasonal | Windspeed | -0.00064 | 0.235196 | -0.0027 | 0.99795 | ns |  |

(**d**) **Comparison of fixed-effect significance between least squares and mixed-effects primary and seasonal guild richness models excluding FLT7.** Mixed-effects models were included as sensitivity analyses to assess the potential influence of non-independence among samples collected on the same date and variance collapsed to zero in this mixed-model. **(b)** Estimated p-values for each predictor in the relevant primary or seasonal multiple linear regression model and the corresponding mixed-effects model including sampling date as a random intercept to evaluate whether predictor significance changed following inclusion of the random effect. Statistical significance: † p < 0.10; * p < 0.05; ** p < 0.01; *** p < 0.001; ns = not significant. Data are provided for all primary and seasonal mixed model date sensitivity analyses that approached statistical significance. For the majority, date variance collapsed to approximately zero. Und. Saprotroph = Undefined Saprotroph.

**Mixed-Effects sensitivity analysis comparisons (excluding FLT7).**

| **Excluding**  **FLT7**  **Analysis** | **Guild** | **Model** | **Random effect** | **Variance** | **SD** |
| --- | --- | --- | --- | --- | --- |
| Dominant | Plant pathogen | Primary mixed | Date | 0 | 0 |
| Dominant | Undefined saprotroph | Primary mixed | Date | 0 | 0 |
| Dominant | Dung saprotroph | Primary mixed | Date | 0.009975629 | 0.099878068 |
| Dominant | Dung saprotroph | Seasonal mixed | Date | 0 | 0 |
| Minor | Endophyte | Primary mixed | Date | 0 | 0 |

| **Excluding**  **FLT7**  **Analysis** | **Guild** | **Model** | **Predictor** | **LS p** | **Mixed p** | **LS Sig.** | **Mixed Sig.** | |
| --- | --- | --- | --- | --- | --- | --- | --- | --- |
| Dominant | Plant Pathogen | Primary | Altitude | 0.569226 | 0.455066 | ns | ns |  |
| Dominant | Plant Pathogen | Primary | Temperature | 0.08833 | 0.025412 | † | * |  |
| Dominant | Plant Pathogen | Primary | Windspeed | 0.009611 | 0.000692 | ** | *** |  |
| Dominant | Plant Pathogen | Seasonal | NDVI | 0.782815 | 0.689471 | ns | ns |  |
| Dominant | Plant Pathogen | Seasonal | Altitude | 0.624517 | 0.478053 | ns | ns |  |
| Dominant | Plant Pathogen | Seasonal | Temperature | 0.126695 | 0.027013 | ns | * |  |
| Dominant | Plant Pathogen | Seasonal | Windspeed | 0.027685 | 0.001453 | * | ** |  |
| Dominant | Und. Saprotroph | Primary | Altitude | 0.964036 | 0.952809 | ns | ns |  |
| Dominant | Und. Saprotroph | Primary | Temperature | 0.232781 | 0.117468 | ns | ns |  |
| Dominant | Und. Saprotroph | Primary | Windspeed | 0.021406 | 0.002566 | * | ** |  |
| Dominant | Und. Saprotroph | Seasonal | NDVI | 0.31588 | 0.14618 | ns | ns |  |
| Dominant | Und. Saprotroph | Seasonal | Altitude | 0.877076 | 0.822587 | ns | ns |  |
| Dominant | Und. Saprotroph | Seasonal | Temperature | 0.464415 | 0.289036 | ns | ns |  |
| Dominant | Und. Saprotroph | Seasonal | Windspeed | 0.020604 | 0.000816 | * | *** |  |
| Dominant | Animal Pathogen | Primary | Altitude | 0.335105 | 0.145064 | ns | ns |  |
| Dominant | Animal Pathogen | Primary | Temperature | 0.677382 | 0.404946 | ns | ns |  |
| Dominant | Animal Pathogen | Primary | Windspeed | 0.688852 | 0.436303 | ns | ns |  |
| Dominant | Animal Pathogen | Seasonal | NDVI | 0.018612 | 0.000669 | * | *** |  |
| Dominant | Animal Pathogen | Seasonal | Altitude | 0.223789 | 0.078045 | ns | † |  |
| Dominant | Animal Pathogen | Seasonal | Temperature | 0.509752 | 0.339373 | ns | ns |  |
| Dominant | Animal Pathogen | Seasonal | Windspeed | 0.107649 | 0.01985 | ns | * |  |
| Dominant | Wood Saprotroph | Primary | Altitude | 0.343656 | 0.214022 | ns | ns |  |
| Dominant | Wood Saprotroph | Primary | Temperature | 0.774741 | 0.707214 | ns | ns |  |
| Dominant | Wood Saprotroph | Primary | Windspeed | 0.912914 | 0.885864 | ns | ns |  |
| Dominant | Wood Saprotroph | Seasonal | NDVI | 0.343078 | 0.16952 | ns | ns |  |
| Dominant | Wood Saprotroph | Seasonal | Altitude | 0.398656 | 0.221307 | ns | ns |  |
| Dominant | Wood Saprotroph | Seasonal | Temperature | 0.890911 | 0.842407 | ns | ns |  |
| Dominant | Wood Saprotroph | Seasonal | Windspeed | 0.781525 | 0.687677 | ns | ns |  |
| Dominant | Endophyte | Primary | Altitude | 0.362451 | 0.178914 | ns | ns |  |
| Dominant | Endophyte | Primary | Temperature | 0.774112 | 0.502086 | ns | ns |  |
| Dominant | Endophyte | Primary | Windspeed | 0.823302 | 0.570579 | ns | ns |  |
| Dominant | Endophyte | Seasonal | NDVI | 0.022036 | 0.000931 | * | *** |  |
| Dominant | Endophyte | Seasonal | Altitude | 0.266963 | 0.107796 | ns | ns |  |
| Dominant | Endophyte | Seasonal | Temperature | 0.423492 | 0.246108 | ns | ns |  |
| Dominant | Endophyte | Seasonal | Windspeed | 0.170942 | 0.047379 | ns | * |  |
| Dominant | Dung Saprotroph | Primary | Altitude | 0.807701 | 1 | ns | ns |  |
| Dominant | Dung Saprotroph | Primary | Temperature | 0.254007 | 0.990353 | ns | ns |  |
| Dominant | Dung Saprotroph | Primary | Windspeed | 0.250368 | 0.991741 | ns | ns |  |
| Dominant | Dung Saprotroph | Seasonal | NDVI | 5.562083e-83 | 1 | *** | ns |  |
| Dominant | Dung Saprotroph | Seasonal | Altitude | 0.556872 | 1 | ns | ns |  |
| Dominant | Dung Saprotroph | Seasonal | Temperature | 3.363907e-77 | 1 | *** | ns |  |
| Dominant | Dung Saprotroph | Seasonal | Windspeed | 5.815581e-78 | 1 | *** | ns |  |
| Minor | Plant Pathogen | Primary | Altitude | 0.256688 | 0.150493 | ns | ns |  |
| Minor | Plant Pathogen | Primary | Temperature | 0.284416 | 0.157845 | ns | ns |  |
| Minor | Plant Pathogen | Primary | Windspeed | 0.830274 | 0.800671 | ns | ns |  |
| Minor | Plant Pathogen | Seasonal | NDVI | 0.145203 | 0.034913 | ns | * |  |
| Minor | Plant Pathogen | Seasonal | Altitude | 0.263577 | 0.105318 | ns | ns |  |
| Minor | Plant Pathogen | Seasonal | Temperature | 0.607383 | 0.456437 | ns | ns |  |
| Minor | Plant Pathogen | Seasonal | Windspeed | 0.692895 | 0.567018 | ns | ns |  |
| Minor | Und. Saprotroph | Primary | Altitude | 0.949832 | 0.838861 | ns | ns |  |
| Minor | Und. Saprotroph | Primary | Temperature | 0.469898 | 0.284084 | ns | ns |  |
| Minor | Und. Saprotroph | Primary | Windspeed | 0.480513 | 0.473453 | ns | ns |  |
| Minor | Und. Saprotroph | Seasonal | NDVI | 0.07505 | 0.009982 | † | ** |  |
| Minor | Und. Saprotroph | Seasonal | Altitude | 0.764847 | 0.664585 | ns | ns |  |
| Minor | Und. Saprotroph | Seasonal | Temperature | 0.990805 | 0.986671 | ns | ns |  |
| Minor | Und. Saprotroph | Seasonal | Windspeed | 0.966312 | 0.951182 | ns | ns |  |
| Minor | Animal Pathogen | Primary | Altitude | 0.494618 | 0.370093 | ns | ns |  |
| Minor | Animal Pathogen | Primary | Temperature | 0.036544 | 0.006126 | * | ** |  |
| Minor | Animal Pathogen | Primary | Windspeed | 0.151923 | 0.060184 | ns | † |  |
| Minor | Animal Pathogen | Seasonal | NDVI | 0.485696 | 0.312316 | ns | ns |  |
| Minor | Animal Pathogen | Seasonal | Altitude | 0.56319 | 0.402091 | ns | ns |  |
| Minor | Animal Pathogen | Seasonal | Temperature | 0.094273 | 0.015428 | † | * |  |
| Minor | Animal Pathogen | Seasonal | Windspeed | 0.140895 | 0.032993 | ns | * |  |
| Minor | Wood Saprotroph | Primary | Altitude | 0.613543 | 0.527081 | ns | ns |  |
| Minor | Wood Saprotroph | Primary | Temperature | 0.571804 | 0.340982 | ns | ns |  |
| Minor | Wood Saprotroph | Primary | Windspeed | 0.694663 | 0.825645 | ns | ns |  |
| Minor | Wood Saprotroph | Seasonal | NDVI | 0.038204 | 0.002716 | * | ** |  |
| Minor | Wood Saprotroph | Seasonal | Altitude | 0.64803 | 0.508177 | ns | ns |  |
| Minor | Wood Saprotroph | Seasonal | Temperature | 0.759735 | 0.657541 | ns | ns |  |
| Minor | Wood Saprotroph | Seasonal | Windspeed | 0.632662 | 0.48843 | ns | ns |  |
| Minor | Endophyte | Primary | Altitude | 0.175843 | 0.075768 | ns | † |  |
| Minor | Endophyte | Primary | Temperature | 0.134874 | 0.049858 | ns | * |  |
| Minor | Endophyte | Primary | Windspeed | 0.693595 | 0.605102 | ns | ns |  |
| Minor | Endophyte | Seasonal | NDVI | 0.365246 | 0.189537 | ns | ns |  |
| Minor | Endophyte | Seasonal | Altitude | 0.214252 | 0.072027 | ns | † |  |
| Minor | Endophyte | Seasonal | Temperature | 0.290885 | 0.125972 | ns | ns |  |
| Minor | Endophyte | Seasonal | Windspeed | 0.99795 | 0.997029 | ns | ns |  |
| Minor | Dung Saprotroph | Primary | Altitude | 0.494552 | 0.370021 | ns | ns |  |
| Minor | Dung Saprotroph | Primary | Temperature | 0.394549 | 0.263895 | ns | ns |  |
| Minor | Dung Saprotroph | Primary | Windspeed | 0.637384 | 0.536166 | ns | ns |  |
| Minor | Dung Saprotroph | Seasonal | NDVI | 0.372395 | 0.196177 | ns | ns |  |
| Minor | Dung Saprotroph | Seasonal | Altitude | 0.448546 | 0.272094 | ns | ns |  |
| Minor | Dung Saprotroph | Seasonal | Temperature | 0.273322 | 0.112513 | ns | ns |  |
| Minor | Dung Saprotroph | Seasonal | Windspeed | 0.936306 | 0.907777 | ns | ns |  |
| All | Plant Pathogen | Primary | Altitude | 0.48894 | 0.363814 | ns | ns |  |
| All | Plant Pathogen | Primary | Temperature | 0.131389 | 0.047832 | ns | * |  |
| All | Plant Pathogen | Primary | Windspeed | 0.4472 | 0.318542 | ns | ns |  |
| All | Plant Pathogen | Seasonal | NDVI | 0.201902 | 0.064545 | ns | † |  |
| All | Plant Pathogen | Seasonal | Altitude | 0.545033 | 0.380395 | ns | ns |  |
| All | Plant Pathogen | Seasonal | Temperature | 0.299795 | 0.133036 | ns | ns |  |
| All | Plant Pathogen | Seasonal | Windspeed | 0.225912 | 0.079412 | ns | † |  |
| All | Und. Saprotroph | Primary | Altitude | 0.906494 | 0.752421 | ns | ns |  |
| All | Und. Saprotroph | Primary | Temperature | 0.328503 | 0.16313 | ns | ns |  |
| All | Und. Saprotroph | Primary | Windspeed | 0.770998 | 0.579939 | ns | ns |  |
| All | Und. Saprotroph | Seasonal | NDVI | 0.061092 | 0.006726 | † | ** |  |
| All | Und. Saprotroph | Seasonal | Altitude | 0.697365 | 0.572972 | ns | ns |  |
| All | Und. Saprotroph | Seasonal | Temperature | 0.748345 | 0.6419 | ns | ns |  |
| All | Und. Saprotroph | Seasonal | Windspeed | 0.251919 | 0.096973 | ns | † |  |
| All | Animal Pathogen | Primary | Altitude | 0.400262 | 0.269681 | ns | ns |  |
| All | Animal Pathogen | Primary | Temperature | 0.10519 | 0.033589 | ns | * |  |
| All | Animal Pathogen | Primary | Windspeed | 0.293266 | 0.167896 | ns | ns |  |
| All | Animal Pathogen | Seasonal | NDVI | 0.26994 | 0.109994 | ns | ns |  |
| All | Animal Pathogen | Seasonal | Altitude | 0.453843 | 0.277708 | ns | ns |  |
| All | Animal Pathogen | Seasonal | Temperature | 0.24269 | 0.090572 | ns | † |  |
| All | Animal Pathogen | Seasonal | Windspeed | 0.174024 | 0.048983 | ns | * |  |
| All | Wood Saprotroph | Primary | Altitude | 0.529874 | 0.419379 | ns | ns |  |
| All | Wood Saprotroph | Primary | Temperature | 0.598011 | 0.394335 | ns | ns |  |
| All | Wood Saprotroph | Primary | Windspeed | 0.729129 | 0.810831 | ns | ns |  |
| All | Wood Saprotroph | Seasonal | NDVI | 0.06502 | 0.007582 | † | ** |  |
| All | Wood Saprotroph | Seasonal | Altitude | 0.552868 | 0.38971 | ns | ns |  |
| All | Wood Saprotroph | Seasonal | Temperature | 0.797216 | 0.709544 | ns | ns |  |
| All | Wood Saprotroph | Seasonal | Windspeed | 0.669449 | 0.536057 | ns | ns |  |
| All | Endophyte | Primary | Altitude | 0.201492 | 0.102339 | ns | ns |  |
| All | Endophyte | Primary | Temperature | 0.279002 | 0.144444 | ns | ns |  |
| All | Endophyte | Primary | Windspeed | 0.984125 | 0.91378 | ns | ns |  |
| All | Endophyte | Seasonal | NDVI | 0.116314 | 0.022985 | ns | * |  |
| All | Endophyte | Seasonal | Altitude | 0.190973 | 0.058222 | ns | † |  |
| All | Endophyte | Seasonal | Temperature | 0.610873 | 0.460816 | ns | ns |  |
| All | Endophyte | Seasonal | Windspeed | 0.48931 | 0.316331 | ns | ns |  |
| All | Dung Saprotroph | Primary | Altitude | 0.787229 | 0.653131 | ns | ns |  |
| All | Dung Saprotroph | Primary | Temperature | 0.522617 | 0.486023 | ns | ns |  |
| All | Dung Saprotroph | Primary | Windspeed | 0.367857 | 0.305145 | ns | ns |  |
| All | Dung Saprotroph | Seasonal | NDVI | 0.098556 | 0.016788 | † | * |  |
| All | Dung Saprotroph | Seasonal | Altitude | 0.602687 | 0.450564 | ns | ns |  |
| All | Dung Saprotroph | Seasonal | Temperature | 0.174969 | 0.04948 | ns | * |  |
| All | Dung Saprotroph | Seasonal | Windspeed | 0.750025 | 0.644202 | ns | ns |  |

LS = Least-squares; Mixed = Mixed-model.

(**e**) **Guild richness model assumption diagnostics** **for guilds containing total (all), dominant, and minor OTUs (excluding FLT7).** Statistical significance: † p < 0.10; * p < 0.05; ** p < 0.01; *** p < 0.001; ns = not significant. Data are provided for all primary and seasonal models excluding FLT7 that approached statistical significance. Und. Saprotroph = Undefined Saprotroph.

| **Guild Richness Model** | **Predictor** | **Variance Inflation Factor (VIF)** |
| --- | --- | --- |
| Primary | Altitude | 1.11886 |
| Primary | Temperature | 2.732585 |
| Primary | Windspeed | 2.71025 |
| Seasonal | NDVI | 2.763978 |
| Seasonal | Altitude | 1.130926 |
| Seasonal | Temperature | 3.258609 |
| Seasonal | Windspeed | 3.170717 |

| **Excluding**  **FLT7**  **Analysis** | **Guild** | **Model** | **Temporal Autocorrelation Test** | **Statistic** | **p-value** | **Sig.** |
| --- | --- | --- | --- | --- | --- | --- |
| Dominant | Plant Pathogen | Primary | Ljung–Box | 1.959377788 | 0.161579879 | ns |
| Dominant | Und. Saprotroph | Primary | Ljung–Box | 0.078196846 | 0.779756025 | ns |
| Dominant | Dung Saprotroph | Primary | Ljung–Box | 5.450653463 | 0.019561009 | * |
| Dominant | Dung Saprotroph | Seasonal | Ljung–Box | 1.819977914 | 0.177315906 | ns |
| Minor | Endophyte | Primary | Ljung–Box | 0.171972189 | 0.678364976 | ns |
